# Bioactive α-Pyrone Derivatives from the Endophytic Fungus *Diaporthe* sp. CB10100 as Inducible Nitric Oxide Synthase Inhibitors

**DOI:** 10.3389/fchem.2021.679592

**Published:** 2021-05-18

**Authors:** Hong Pu, Jianxin Liu, Yeji Wang, Yuhui Peng, Wanying Zheng, Yang Tang, Boping Hui, Chunmei Nie, Xueshuang Huang, Yanwen Duan, Yong Huang

**Affiliations:** ^1^ Xiangya International Academy of Translational Medicine, Central South University, Changsha, China; ^2^ School of Pharmaceutical Sciences, Hunan University of Medicine, Huaihua, China; ^3^ Hunan Provincial Key Laboratory for Synthetic Biology of Traditional Chinese Medicine, Hunan University of Medicine, Huaihua, China; ^4^ Hunan Engineering Research Center of Combinatorial Biosynthesis and Natural Product Drug Discover, Changsha, China; ^5^ National Engineering Research Center of Combinatorial Biosynthesis for Drug Discovery, Changsha, China

**Keywords:** endophytic fungus, *Diaporthe* sp., α-pyrone, anti-inflammation, inducible nitric oxide synthase, NO

## Abstract

Inducible nitric oxide synthase (iNOS) produces NO from l-arginine and plays critical roles in inflammation and immune activation. Selective and potent iNOS inhibitors may be potentially used in many indications, such as rheumatoid arthritis, pain, and neurodegeration. In the current study, five new compounds, including a dibenzo-α- pyrone derivative ellagic acid B (**5**) and four α*-*pyrones diaporpyrone A–D (**9–12**), together with three known compounds (**6**–**8**), were isolated from the endophytic fungus *Diaporthe* sp. CB10100. The structures of these new natural products were unambiguously elucidated using NMR, HRESIMS or electronic circular dichroism calculations. Ellagic acid B (**5**) features a tetracyclic 6/6/6/6 ring system with a fused 2*H*-chromene, which is different from ellagic acid (**4**) with a fused 2*H*-chromen-2-one. Both 2-hydroxy-alternariol (**6**) and alternariol (**7**) reduced the expression of iNOS at protein levels in a dose-dependent manner, using a lipopolysaccharide (LPS)-induced RAW264.7 cell models. Also, they decreased the protein expression levels of pro-inflammatory cytokines, such as tumor necrosis factor-α, interleukin-6 and monocyte chemotactic protein 1. Importantly, **6** and **7** significantly reduced the production of NO as low as 10 μM in LPS-induced RAW264.7 cells. Molecular docking of **6** and **7** to iNOS further suggests that both of them may interact with iNOS. Our study suggests that **6** and **7**, as well as the alternariol scaffold may be further developed as potential iNOS inhibitors.

## Introduction

Inflammation plays important roles in the occurring and development of many diseases, including rheumatoid arthritis (RA) (Choy and Panayi, 2001), osteoarthritis (OA) (Ahmad et al., 2020), diabetes (Purkayastha and Cai, 2015), and cancers (Zhong et al., 2016). When inflammation occurs, excessive inflammatory mediators are produced by inducible nitric oxide synthase (iNOS) or cyclooxygenase-2 (COX-2), such as NO and prostaglandin E2 (PGE_2_) (Yu et al., 2019). Non-steroidal anti-inflammatory drugs (NSAIDs) and steroid hormone glucocorticoids are both used for the treatment of inflammation, despite serious side effects (Buchman, 2001; Salvo et al., 2011). iNOS is a mammalian protein composed of a C-terminal reductase and an N-terminal oxygenase domain, which produces micromolar NO by oxidizing l-arginine to l-citrulline in the presence of bacterial lipopolysaccharide (LPS) and/or proinflammatory cytokines. A considerable number of iNOS inhibitors, such as arginine derivatives, pyrimidines and aminopyrimidines, as well as aliphatic, aromatic, and cyclic amidines, have been developed (Cinelli et al., 2020). Although many of them showed promise in the treatment of arthritis or inflammatory and neuropathic pain in animal models, there are no iNOS inhibitors on the market. Therefore, there is strong need to discover new iNOS inhibitors as anti-inflammation agents.

**FIGURE 1:**
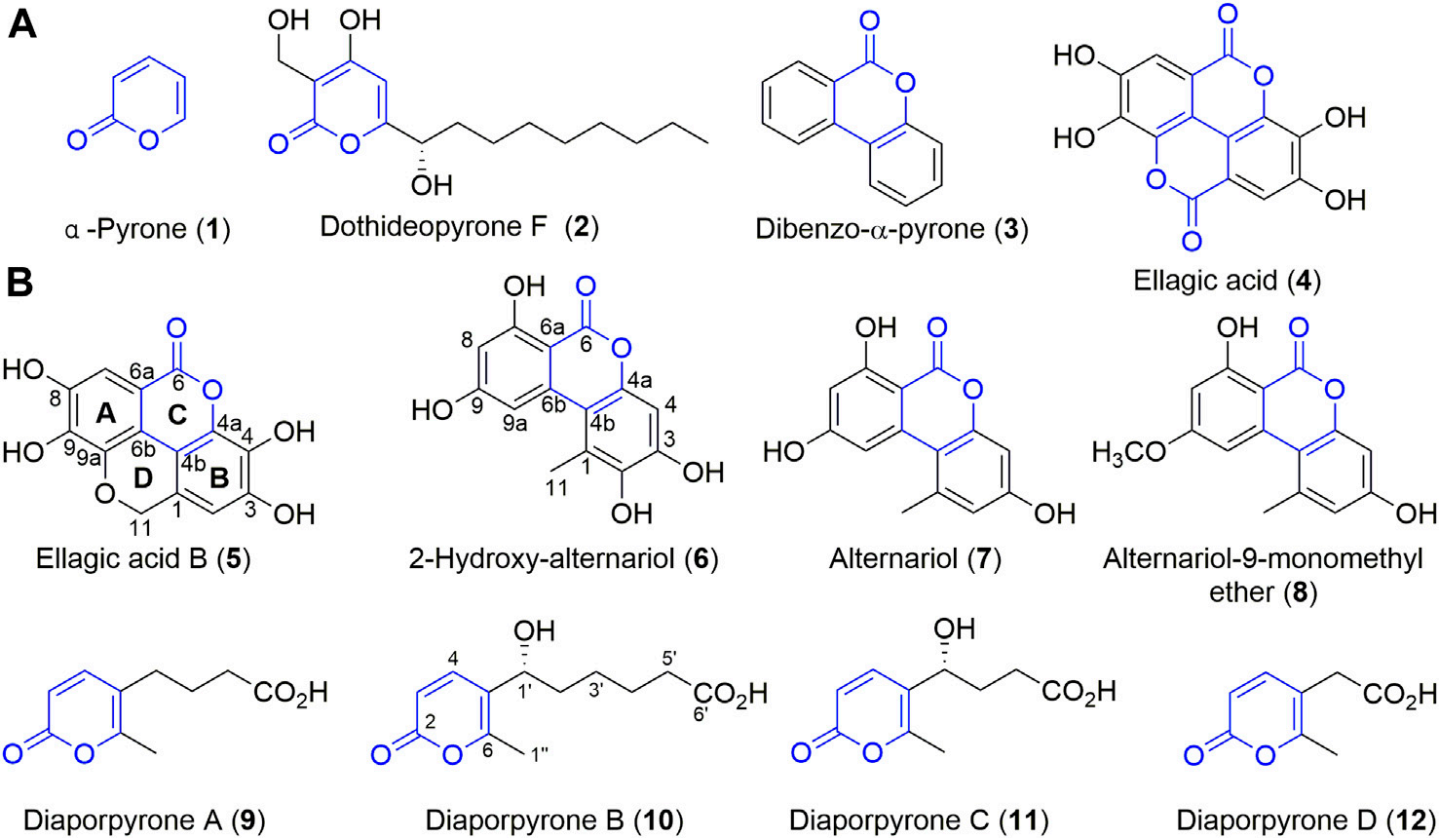
Structures of compounds **1–12. (A)** The structures of dibenzo-α-pyrones, α-pyrone, and their derivatives. **(B)** Compounds isolated from endophilic fungus strain *Diaporthe* sp. CB10100.

α-Pyrone (**1**, Figure 1A) is an aromatic unsaturated lactone and an important sub-structure of various natural products with interesting biological activities (Mcglacken and Fairlamb, 2005). For example, dothideopyrone F (**2**) inhibited NO production in LPS-induced BV2 cells and diminished the protein expression levels of iNOS and COX-2 (Kim et al., 2018). In addition, natural products with α-pyrone sub-structure have antibacterial (Zhang et al., 2013), anti-influenza A virus (Hou et al., 2018), and anti-HIV (Appendino et al., 2007) activities. Some of them are signal molecules (Brachmann et al., 2013), while some are also cytotoxic agents against tumor cells (Rivera-Chávez et al., 2019; Zhu et al., 2019). In particular, dibenzo-α-pyrone (**3**) belongs to a significant group of heptaketide coumarin metabolites that have a fused tricyclic core (Figure 1A), commonly identified from fungi, bacteria, and plants (Lai et al., 2016). Many of them also display diverse biological activities including cytotoxicity and anti-inflammatory activity (Mao et al., 2014). For example, ellagic acid **(4)**, found in medicinal plants, vegetables and fruits, exhibits impressive anti-inflammatory and anti-diabetic activities (Ríos et al., 2018). It is widely used in skin care products because of its anti-oxidation effect and skin protection effects.

Sinomenine (Supplementary Figure S1), a tetrahydroisoquinoline-type alkaloid isolated from the medicinal plant of *Sinomenium acutum* (Thunb.) (“青风藤” in Chinese), has strong anti-inflammatory effects and is used in China to treat RA for many years (Zhao et al., 2012; Wang et al., 2019). To our knowledge, there are few reports of endophytes and their natural products from *S. acutum*. Since endophytes from medical plants are attractive sources of bioactive natural products, we initiated an endeavor to isolate natural products from endophytes colonized in the stem and root of *S. acutum*, in our continuous search for new bioactive metabolites of microorganisms from un- or underexplored niches (Jiang et al., 2018; Jiang et al., 2021a). In this study, we report that these accumulating efforts have resulted in the isolation of multiple endophytes from *S. acutum*, and bioactivity-guided natural product isolation have yielded eight compounds, including a new dibenzo-α-pyrone derivative, ellagic acid B (**5**) and four new α-pyrone diaporpyrone A–D (**9**–**12**), together with three known compounds (**6**–**8**). Further biological evaluation of these compounds revealed that 2-hydroxy-alternariol (**6**) and alternariol (**7**) are potential iNOS inhibitors.

## Materials and Methods

### General Methods

The instruments (including those applied for optical rotations, MS, ORD, NMR, and ECD) and routine reagents for chemical isolation and biological evaluation were the same as those reported previously (Jiang et al., 2021a). The details were listed in the Supporting Information.

### Isolation of Endophytes and Fermentation

The separation methods of endophytic fungi are detailed in the Supporting Information. These endophytic fungi CB10098-CB10104 were isolated from the stems and roots of *S. acutum*, which were collected from Huaihua (Hunan, China) in October 2017. They were grown in PDA (0.3% potato extract, 2% glucose, 1.5% agar power) medium at 28°C for 5 day, and then inoculated to seed medium [glucose 2%, sucrose 1%, soybean powder 0.2%, peptone 1%, K_2_HPO_4_ 0.03%, poly (ethylene glycol) 0.25%, NaNO_3_ 0.3%, and (NH_4_)_2_SO_4_ 0.3%]. The final pH of the medium was adjusted to 6.0 before sterilization with an autoclave at 121°C for 20 min. Cultures were incubated in flasks at 28°C on a rotary shaker at 220 rpm for 3 day to prepare the seed culture. Next, they were transferred to 2 × 500 ml Erlenmeyer flasks contained rice (50 g) and H_2_O (50 ml). After sterilization in an autoclave at 121°C for 30 min, each flask was inoculated with 10 ml of seed culture and incubated at 28°C for 40 day. Finally, the solid rice culture containing the respective fungus were extracted by ethyl acetate to obtain extracts C1–C6.

### Fungal Strain

The strain CB10100 was identified as *Diaporthe* sp. according to molecular identification and its ITS sequence has 99% sequence identity to that of *Diapo* *rthe* sp. The sequence data have been deposited to GenBank with the accession number MW037206. The strain was deposited in the Xiangya International Academy of Translational Medicine, Central South University, Changsha, Hunan, China.

### Large-Scale Fermentation of *Diaporthe* sp. CB10100

For large-scale fermentation of *Diaporthe* sp. CB10100, 185 × 1 L Erlenmeyer flasks containing rice (100 g) and H_2_O (100 ml) were used. The fermented solid rice culture was supersonically extracted with EtOAc (3 × 60 L), and after recovering the organic solvent, the crude extract was suspended in water and successively treated with petroleum ether, ethyl acetate, and n-butanol.

### Isolation of Compounds 5–12

The EtOAc-soluble fraction (192.8 g) was subjected to silica gel column chromatography using petroleum/EtOAc (v/v, 20:1 → 10:1 → 5:1 → 1:1 → 1:5 → 1:20), EtOAc, EtOAc/MeOH (v/v 1:0 → 1:5 → 0:1)to yield nine combined fractions (Fr. A to J). Fr. B (27.13 g) was chromatographed on ODS column (MeOH/H_2_O v/v 3:7 → 4:6 → 5:5 → 6:4 → 7:3) to yield six combined fractions (Fr. A1 to A6). Fr. A3 was separated by Sephadex LH-20 by employing MeOH/CH_2_Cl_2_ (7:3, v/v) as mobile phase, followed by purification using semi-preparative HPLC with isocratic MeCN/H_2_O [containing 0.2% formic acid, MeCN/H_2_O, v/v, 10: 90 → 70:30 (0–11 min); 70:30 (11–12 min); 70:30 → 10:90 (12–20 min); 10:90 (20–21 min)] as mobile phase to yield **6** (10.6 mg), **7** (30.6 mg), along with known **8** (5.3 mg). Following the same procedure, **5** (4.6 mg) was obtained from Fr. A5.

The petroleum ether fraction (447.6 g) was subjected to silica gel column chromatography using a step gradient elution of petroleum/EtOAc (v/v, 20:1 → 5:1 → 1:1 → 1:10), EtOAc, EtOAc/MeOH (v/v 1:0 → 10:1 → 1:1 → 0:1) to yield ten combined fractions (Fr. 2A to 2H). Fr. 2 J (57.86 g) was chromatographed on ODS column (MeOH: H_2_O v/v 3:7 → 4:6 → 5:5 → 6:4 → 7:3) to yield ten combined fractions (Fr. 2J-1 to 2J-10). Fr. 2J-2 (1.32 g) was chromatographed on ODS column (MeOH/H_2_O v/v 3:7 → 4:6 → 5:5 → 6:4 → 7:3), Sephadex LH-20 (MeOH) column and repeated purification by semipreparative HPLC [containing 0.2% formic acid, MeCN/H_2_O, v/v, 5: 95 → 25:75 (0–11 min); 25:75 (11–12 min); 25:75 → 5:95 (12–20 min); 5:95 (20–21 min)], which resulted in **9** (4.3 mg) and **10** (5.6 mg). In the same manner, **11** (2.7 mg) and **12** (1.0 mg) were obtained from Fr. 2J - 1.

#### Ellagic acid B (**5**)

Dark-grey powder; UV (MeOH) λmax 200.9, 255.2, 287.2, 363.7 (see Supplementary Figure S4A); ^1^H, ^13^C and 2D NMR spectroscopic data, see Table 1 and Supplementary Figures S7–S13; HRESIMS *m/z* 287.0197 [M - H]^−^ (calcd for C_14_H_7_O_7_, 287.0197).

**TABLE 1:** ^1^H NMR (500 MHz) and ^13^C NMR (125 MHz) data of **5** and **6** in DMSO-*d*_6_.

Position	**5**		**6**	
	δ_C,_ type	δ_H_, mult. (J in Hz)	δ_C_, type	δ_H_, mult. (J in Hz)
1	120.4, C		122.0, C	
2	101.5, CH	6.69, s	141.7, C	
3	149.4, C		147.7, C	
4	142.1, C		101.0, CH	6.7, s
4a	102.8, C		144.9, C^*^	
4b	138.2, C		109.2, C	
6	163.9, C		165.2, C^*^	
6a	129.8, C		97.8, C	
6b	94.1, C		138.7, C^*^	
7	102.4, CH	6.31, s	164.1, C	
7-OH				11.87, s
8	154.2, C		100.8, C	6.34, br s
9	156.5, C		165.0, C	
9a	114.4, C			
10			104.4, CH	7.26, br s
11	63.3, CH_2_	5.30, s	16.0, CH_3_	2.58, s

* Not detected (Chapla et al., 2014).

#### Diaporpyrone A (9)

White powder; UV (MeOH) λmax 202.1, 220.9, 308.7 (see Supplementary Figure S4E); ^1^H, ^13^C and 2D NMR spectroscopic data, see Table 2; Supplementary Figures S35–S48; HRESIMS *m/z* 195.0655 [M - H]^−^ (calcd for C_10_H_11_O_4_,195.0663).

**TABLE 2:** ^1^H NMR (^a^500 MHz) or (^b^400 MHz) and ^13^C NMR (^a^125 MHz) or (^b^100 MHz) data of **9–12** in CD_3_OD.

Position	9^b^		10^a^		11^b^		12^a^	
	δ_C_, type	δ_H_, mult. (J in Hz)	δ_C_, type	δ_H_, mult. (J in Hz)	δ_C_, type	δ_H_, mult. (J in Hz)	δ_C_, type	δ_H_, mult. (J in Hz)
2	165.2, C		164.8, C		165.3, C		165.2, C	
3	113.7, CH	6.17, d (9.6)	114.1, CH	6.23, d (9.5)	113.7, CH	6.20, d (9.6)	112.8, CH	6.18, d (9.5)
4	149.6, CH	7.44, d (9.6)	145.9, CH	7.62, d (9.5)	149.7, CH	7.48, d (9.6)	150.2, CH	7.45, d (9.5)
5	116.9, C		120.6, C		116.8, C		113.2, C	
6	160.4, C		160.0, C		160.6, C		161.7, C	
1'	29.5, CH_2_	2.41, t (7.2)	68.6, CH	4.61, t (7.0)	70.9, CH	4.08, m	35.4, CH_2_ ^*^	3.36, s
2'	26.2, CH_2_	1.79, m	37.7, CH_2_	1.76, m 1.57, m	35.2, CH_2_	1.96, m 1.86, m	175.7, C^*^	
3'	34.4, CH_2_	2.33, t (7.2)	26.2, CH_2_	1.36, m	25.9, CH_2_	2.52, m		
4'	176.8, C		26.0, CH_2_	1.63, m	176.7, C			
5'			35.2, CH_2_	2.29, t (4.5)				
6'			178.1, C					
1''	17.2, CH_3_	2.27, s	17.0, CH_3_	2.27, s	17.2, CH_3_	2.32, s	17.6, CH_3_	2.26, s

* Not detected.

#### Diaporpyrone B (10)

White powder; UV (MeOH) λmax 196.2, 301.5 (see Supplementary Figure S4F) [α] ^23.6^_D_ +1.92 (c 0.12, MeOH); ECD (MeOH) λ (ε) 303 (−1.22), 233 (2.02), 208(−5.97); ^1^H, ^13^C and 2D NMR spectroscopic data, see Table 2; Supplementary Figures S49–S56; HRESIMS *m/z* 239.0922 [M - H]^-^ (calcd for C_12_H_15_O_5_, 239.0925).

#### Diaporpyrone C (11)

White powder; UV (MeOH) λmax 197.4, 223.3, 307.5 (see Supplementary Figure S4G) [α] ^23.6^_D_ 170+14.72 (c 0.072, MeOH); ^1^H, ^13^C and 2D NMR spectroscopic data, see Table 2; Supplementary Figures S57–S64; HRESIMS *m/z* 211.0607 [M - H]^−^ (calcd for C_10_H_11_O_5_, 211.0612).

#### Diaporpyrone D (12)

White powder; UV (MeOH) λmax 199.7, 220.9, 303.9 (see Supplementary Figure S4H); ^1^H, ^13^C and 2D NMR spectroscopic data, see Table 2; Supplementary Figures S65–S71; HRESIMS *m/z* 167.0340 [M - H]^−^ (calcd for C_8_H_7_O_4_, 167.0350).

### ECD Calculation Methods

The ECD spectrum of diaporpyrone B **(10)** was calculated by using of the Gaussian 09 package (Frisch et al., 2010). Those configurations were optimized at the B3LYP/6-31G (d) level. The ECD spectrum were calculated by the TDDFT method at the B3LYP/6–311+ +G (2 day, *p*) level with the CPCM model in methanol solution (Bruhn et al., 2013). The details were listed in the Supporting Information.

### Cell Culture and Cell Viability Assay

Cell culture and cell viability assay (Liu et al., 2016) was utilized to investigate the maximum concentration of every compound that was not toxic to cells. The method for cell culture and cell viability assay was in the Supplementary Material.

### Western Blotting for the Detection of iNOS and COX-2

The iNOS and COX-2 protein levels affected by compounds **5**–**12** were detected using Western blotting according to the reported procedures, and dexamethasone was used as the positive control (Jiang et al., 2021a). The procedure is detailed in the Supplementary Material.

### Detection of NO and Inflammatory Cytokines Production

RAW264.7 cells were seeded in 12-well plates in a density of 2 × 10^5^ cells/mL and incubated for 24 h. Three different concentration compounds were added in the each well for 1 h before LPS stimulation. The culture medium was collected and stored at −80°C. The levels of nitric oxide in the culture medium were detected using the Nitric oxide detection kit (Invitrogen) and the levels of TNF-α, IL-6 and MCP-1 were analyzed using the ELISA kits (eBioscience).

### Molecular Docking Analysis

Molecular docking studies were conducted by the software Molecular Operating Environment (MOE 2010.06; Chemical Computing Group, Canada) as the computational platform. The procedure is detailed in the Supplementary Material.

### Statistical Analysis

The results are expressed as mean ± SD of at least three independent experiments. Statistical significance of differences between groups was determined by ANOVA, and a level of *p < 0.05 or **p < 0.01 was considered statistically significant.

## Results and Discussion

### Isolation of Endophylic Fungi From *Sinomenium acutum*

Six endophytic fungi CB10098-CB10104 were isolated from the stems and roots of *S. acutum*, collected in Huaihua city in central China (Supplementary Figure S1). These endophytic microorganisms were fermented and the anti-inflammatory activity of their crude extracts C1–C6 was evaluated in a lipopolysaccharide (LPS)-stimulated RAW264.7 cells. Several crude extracts, e.g., C3 (from CB10100) and C5 (from CB10102), showed the specific inhibition against iNOS at a concentration of 20 μg/ml (Supplementary Figure S1C). Next, the fungus CB10100 was identified as a *Diaporthe* species based on DNA sequencing of its internal transcribed spacer 4 (ITS4) and the phylogenetic analysis of its ITS4 with selected *Diaporthe* strains in GenBank (Supplementary Figure S2). *Diaporthe,* the sexual morph of *Phomopsis* and a known plant pathogen (Huang et al., 2013), lives in a diversity of plants (Guarnaccia and Crous, 2017). Some *Diaporthe* or *Phomopsis* species can also produce bioactive small molecules and protect host plants from fungi infection (Tan and Zou, 2001). For example, several bioactive metabolites were discovered, including anthraquinones (*i.e.,* cytoskyrin A), terpenes (*i.e.,* diaporthein A), alkaloids (*i.e.,* diaporisoindoles A) (Chepkirui and Stadler, 2017) (Supplementary Figure S3). Therefore, a scale-up solid fermentation of *Diaporthe* sp. CB10100 (185 × 1 L flasks containing 100 g boiled rice) was next used to isolate natural products with anti-inflammatory activity.

### Structure Elucidation

The obtained *Diaporthe* sp. CB10100 crude extract was further fractioned based on silico gel and Sephadex LH-20 chromatography and semi-preparative HPLC, which yielded compounds **5–12**. Alternariol (**7**) and alternariol-9-monomethyl ether (**8**) are known compounds, and their structures were established based on the comparison of their 1D and 2D NMR spectra, HRMS, and UV spectra with the literature (Supplementary Figures S4C,D, S6C,D, S21–S34) (Hildebrand et al., 2015). Although 2-hydroxy- alternariol **(6)** was reported in 2007 and 2014 (Pfeiffer et al., 2007; Chapla et al., 2014), three carbons signals of **6** were not identified. Therefore, a complete set of NMR assignment of **6** is listed in Table 1, based on our full set of NMR spectra (Supplementary Figures S14–S20).

**FIGURE 2:**
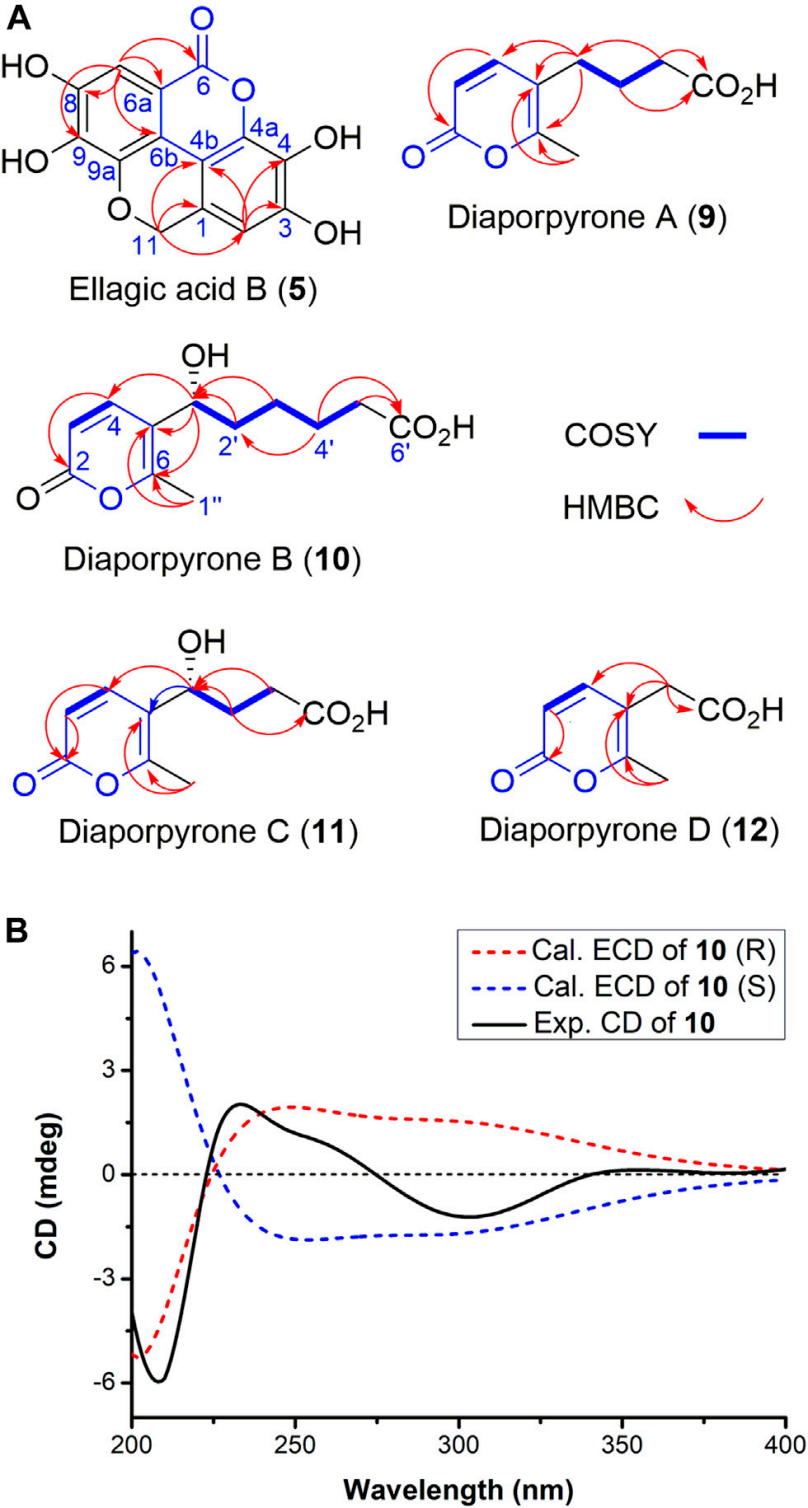
**(A)** Key ^1^H–^1^H COSY and HMBC correlations of compounds **5** and **9–12**. **(B)** Experimental and calculated ECD spectra of compounds **10** in MeOH.

Ellagic acid B **(5)** was isolated as colorless powder. Its molecular formula is established as C_14_H_8_O_7_ based on the (-) -HRESIMS analysis at *m/z* 287.0197 [M - H]^−^ (calcd for C_14_H_7_O_7_, 287.0197), suggesting 11 degrees of unsaturation. Its ^13^C NMR spectrum (Table 1) shows a total of 14 signals that could be classified as an ester carbonyl (*δ*_C_, 163.9), 12 sp^2^-hybridized carbons (including two methine and six quaternary carbons), and one oxygenated aliphatic carbon (*δ*_C_ 63.3), assisted by DEPT-135 and HSQC spectra (Supplementary Figures S10, S11). The presence of two aromatic singlet protons at *δ*_H_ 6.31 (s) and 6.69 (s) suggests the presence of a likely biphenyl structure. Based on these analyses and the similar UV spectra with **6**–**8**, compound **5** may contain a new dibenzo-α-pyrone ring system with an additional fused α-pyran moiety. This was corroborated by its HMBC spectrum, showing correlations from the aromatic proton H-2 (*δ*_H_ 6.69, s) to C-1, C-3, C-4, C-4b, and from H-7 (*δ*_H_ 6.31, s) to C-6, C-6a, C-6b, C-8, and C-9, as well as from one methylene (*δ*_H_ 5.29, s) to C-1, C-2, C-4b, C-9a (Figure 2). To our knowledge, this represents a new tetracyclic 6/6/6/6 ring system derived from dibenzo-α-pyrones.

Diaporpyrone A **(9)** was isolated as a colorless powder. Its molecular formula was determined as C_10_H_12_O_4_ on the basis of HRESIMS analysis, affording an [M - H]^−^ ion at *m/z* 195.0655 [M - H]^−^ (calculated for [M - H]^−^ ion at *m/z* 195.0663). The ^1^H and ^13^C NMR data of **9** are similar to a previously isolated pyrone with a six-carbon aliphatic chain from an endophytic fungus *Pericinia* sp. F-31, with the replacement of the terminal methyl group by a carboxylic acid (*δ*_C_ 178.1) (Table 2; Supplementary Figure S36; Zhang et al., 2015). This was confirmed by the HMBC correlations from 1″ -CH_3_ to C-5 (*δ*_C_ 116.9) and C-6 (*δ*_C_ 160.4), and from C-3 ^ʹ^ (*δ*_C_ 34.4) to C-4 ^ʹ^ (*δ*_C_ 176.8) and C-2^ʹ^ (*δ*_C_ 26.2). We named the new α-pyrone **9** as diaporpyrone A, since it is from the fungus *Diaporthe* sp. CB10100.

Diaporpyrone B **(10)** was isolated as colorless powder. Its molecular formula was established as C_12_H_16_O_5_ based on the (-)-HRESIMS analysis at m/z 239.0922 [M - H]^−^ (calcd for C_12_H_15_O_5_, 239.0925). The ^13^C NMR and DEPT spectra of 10 shows a total of 12 signals containing two carbonyl carbon (*δ*_C_ 178.1 and 164.8), two nonprotonated carbons (*δ*_C_ 160.0, 120.6), two olefinic methine carbon (*δ*_C_ 145.9, 114.1), an oxymethine carbons (*δ*_C_ 68.6), four methylene carbons (*δ*_C_ 37.7, 35.2, 26.2, and 26.0), and one methyl carbon (*δ*_C_ 17.0) (Table 2). The UV spectra of **10** resembled that of **9**, and most of the ^1^H and ^13^C NMR data of **10** are similar to those of **9**. In the ^1^H NMR, ^13^C NMR, and HSQC spectra of **10**, additional signals attributed to the presence of a hydroxy group on C-1′ (*δ*_C_ 68.6). This was confirmed by the HMBC correlations from C-1′ to C-4 (*δ*_C_ 145.9), C-5 (*δ*_C_ 120.6), C-6 (*δ*_C_ 160.0), and the correlations of H-1′/H_2_-2′/H_2_-3′/H_2_-4′/H_2_-5′ in the COSY spectrum of **10** (Figure 2A). Electronic circular dichroism (ECD) calculations were next employed to determine the absolute configuration of **10** by comparing the ECD spectra of (1ʹ*S*)**-10** and (1ʹ*R*)**-10** with the experimental result, which suggests that a (1ʹ*R*)**-10** configuration (Figure 2B; Bruhn et al., 2013). In addition, the specific rotation value [α]^23.6^_D_ +1.92 (c 0.12, MeOH) was in opposite with the published specific rotation value of similar α-pyrones dothideopyrone A ([α]^25^_D_ -77 (c 0.22, CHCl_3_)) (Chomcheon et al., 2009) and dothideopyrone F ([α]^25^_D_ -118.72 (c 0.05, MeOH)) (Kim et al., 2018), with an *S*-configuration at C-1′.

Diaporpyrone C **(11)** was isolated as colorless powder, and its molecular formula was determined as C_10_H_12_O_5_ by the (-)-HRESIMS *m/z* 211.0607 [M - H]^−^ (calcd for C_10_H_11_O_5_, 211.0612), differing from the molecular formula of **10** by two methylenes. The UV spectrum and 1D and 2D NMR data of **11** are similar to those of **10** (Supplementary Figures S4F,G; Table 2), suggesting that **10** and **11** are similar α-pyrones with a fatty acid side chain. The complete structure of **11** was fully assigned based on ^1^H−^1^H COSY, HSQC, and HMBC data (Figure 2A; Table 2). Since diaporpyrones B **(10)** and C **(11)** likely share the same biosynthetic route, diaporpyrone C **(11)** may have the same absolute configuration at C-1′ position, which is further supported by its specific rotation value [α]^23.6^_D_ +14.72 (c 0.072, MeOH), similar to that of diaporpyrone B **(10)**.

Diaporpyrone D **(12)** was isolated as colorless powder, and its molecular formula was determined as C_8_H_8_O_4_ by the (-)-HRESIMS *m/z* 167.0340 [M - H]^−^ (calcd for C_8_H_7_O_4_, 167.0350), differing from the molecular formula of **9** by two methylenes. The UV maximum absorption and 1D NMR data were highly similar to those of **9** (Table 2; Supplementary Figures S4E,H), suggesting the presence of a similar carbon skeleton. The signals for 35.4 and 175.7 ppm are not present in the ^13^C NMR spectrum of **12**, while they are present in both HSQC and HMBC spectra (Supplementary Figures S69, S70). The complete structure of **12** was fully assigned based on these data. The complete structure of **12** was fully assigned based on ^1^H−^1^H COSY, HSQC, and HMBC data (Figure 2A; Table 2).

### Biosynthetic Pathway Speculation for Compounds 5–12

**FIGURE 3:**
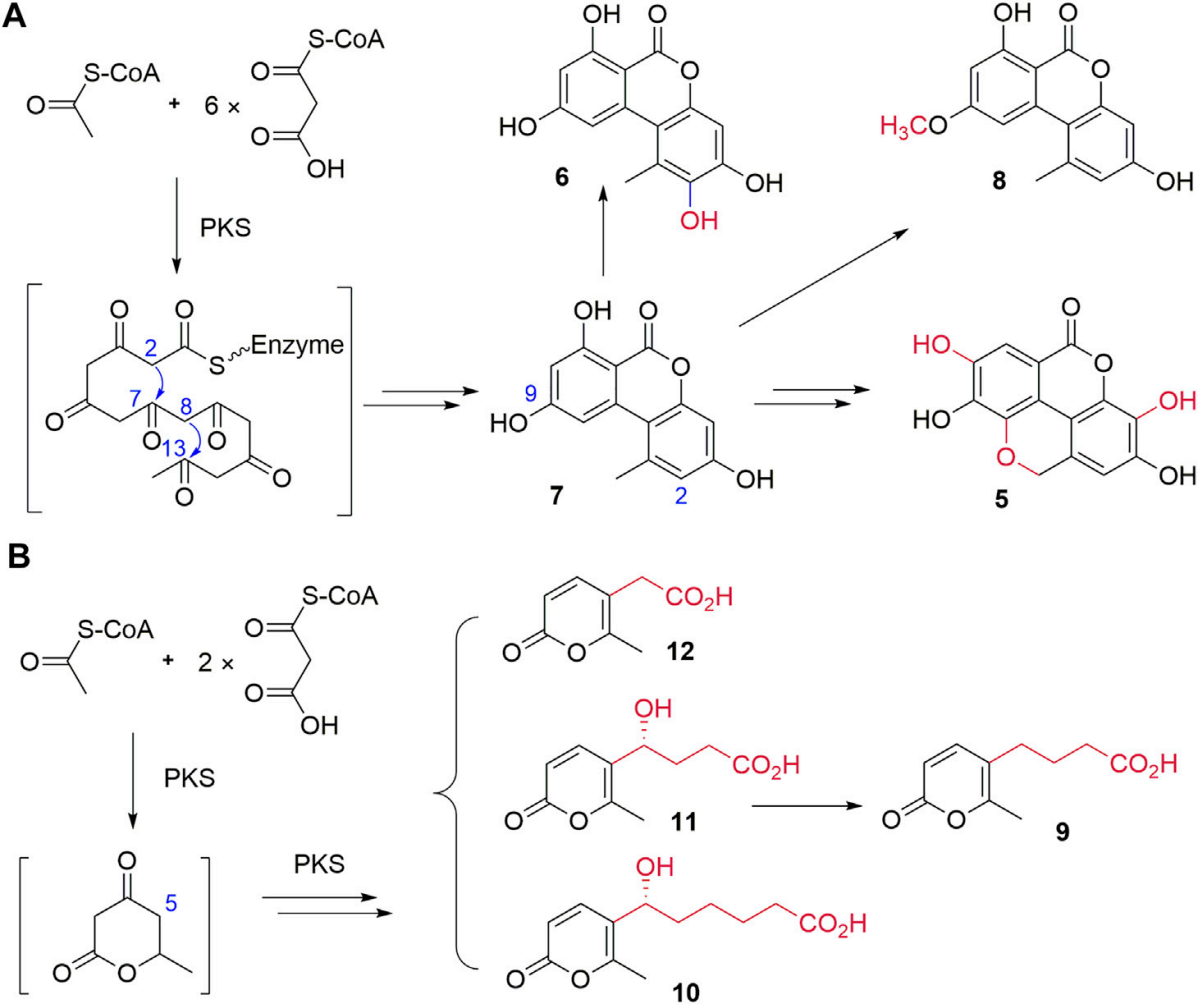
Proposed biosynthetic pathways of **5**–**8** **(A)** and **9**–**12** **(B)**.

The biosynthesis of **7** and **8** was proposed in 2012, while their biosynthetic enzyme SnPKS19 was identified from a wheat pathogen *Parastagonospora nodorum* (Kim et al., 2012; Chooi et al., 2015). Therefore, the biosynthesis of dibenzo-α-pyrone **5**–**8** in *Diaporthe* sp. CB10100 may follow the similar biosynthetic logic, which involves the condensation of seven molecules of malonyl-CoA, followed by aldol-type cyclization between C-2 and C-7, and C-8 and C-13, and the subsequent lactonization leads to the key intermediate **7** (Figure 3A). Subsequent methylation of the C-5 hydroxyl group of **7** by a methyltransferase would lead to **8**, and hydroxylation of C-2 of **7** may produce **6**. Further multi-step modifications of **7** may be envisioned to generate the unique conjugated four ring system of **5**, despite an alternative precursor of **5** may also be possible.

The biosynthesis of pyrone **9–12** may involve the condensation of three molecules of malonyl-CoA, followed by aldol-type cyclization and the subsequent lactonization to generate the key six-membered lactone intermediate by a PKS (Figure 3B). Next, the additional fatty acid side chains with various lengths may be biosynthesized by a PKS and finally attached to the lactone to generate **9–12**. The detailed biosynthetic mechanism of **9–12** remains to be elucidated.

### Effects of Compounds 5–12 on LPS-Induced iNOS and COX-2 Expression in RAW264.7 Cells

**FIGURE 4:**
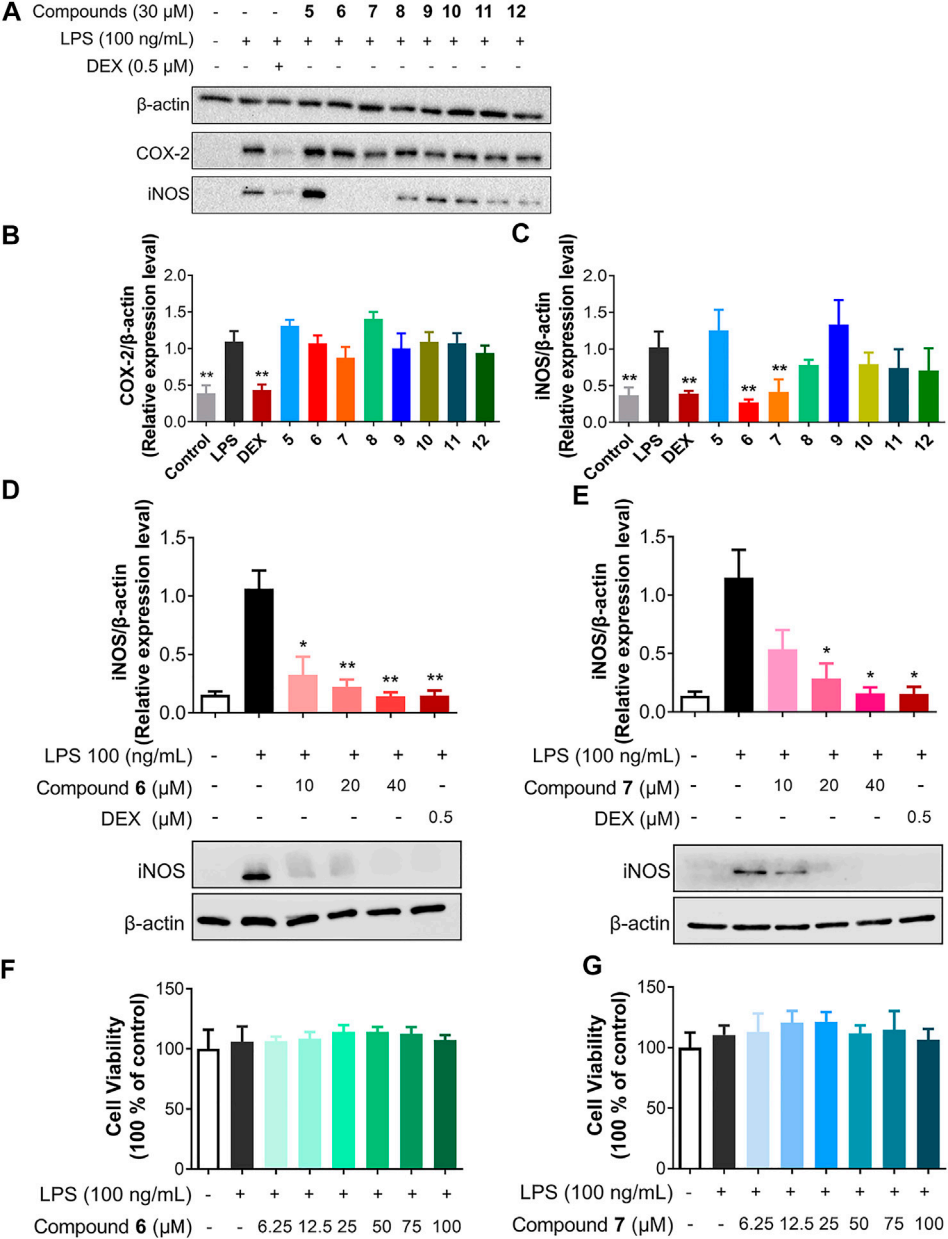
Compounds **6** and **7** specifically inhibit iNOS. **(A)** Inhibitory effects of **5**–**12** on LPS-induced iNOS and COX-2 protein expression in RAW264.7 cells. The cells were pretreated with **5**–**12** (30 μM) for 1 h and then stimulated by LPS (100 ng/ml) for 18 h. **(B and C)** The protein levels of iNOS **(B)** and COX-2 **(C)** were quantitated using ChemiDoc^TM^ XRS^+^ with image Lab^TM^ software (Biorad) and the density ratio of iNOS or COX-2 to β-actin were shown. The density ratio of LPS only group was set to 1. **(D and E)** Effects of **6** and **7** with concentrations of 10, 20, and 40 μM on the protein expression of iNOS. The results were the mean ± SD, n = 4. *p < 0.05, **p < 0.01, vs. LPS alone. **(F,G)** The cytotoxicity of **6** and **7** on RAW264.7 cells.

iNOS and COX-2 are key proteins in the inflammatory signaling pathway. When inflammation occurs, iNOS and COX-2 are usually highly expressed, resulting the formation of inflammatory mediators, including NO and PGE_2_ (Kyoung et al., 2019). Many phenolic compounds exhibit anti-inflammatory activities through inhibition of the iNOS (Ku et al., 2008; Laavola et al., 2015). No anti-inflammatory activity of compound **6** was reported (Pfeiffer et al., 2007; Chapla et al., 2014), while **7** was discovered to modify macrophage phenotype and to inhibit production of NO and inflammatory responses (Solhaug et al., 2015; Chen et al., 2020). In addition, dothideopyrone F (**2**) was able to diminish the expression of iNOS and COX-2 in lipopolysaccharide (LPS)-induced BV2 cells (Kim et al., 2018). Therefore, we hypothesize that the newly isolated natural products, e.g., **5–6** and **9–12**, may have certain anti-inflammatory activity. In lipopolysaccharide (LPS)-stimulated RAW264.7 cell model, we next evaluated their inhibitory activity of iNOS and COX-2 protein expression, along with **7** and **8**, using dexamethasone (DEX) as a positive control (Figures 4A–C).

Among the tested compounds, only **6** and **7** strongly inhibited iNOS protein expression, while they had no effects on COX-2 expression in a concentration of 30 μM (Figure 4A). To compare the anti-inflammatory activity of **6** and **7**, the anti-inflammatory activity of **6** and **7** in concentrations ranging from 10 to 40 μM was determined in LPS-stimulated RAW264.7 cells. Both compounds inhibit the expression of iNOS in a dose-dependent manner (Figures 4D,E). For example, **6** was able to inhibit about 90% of iNOS expression when applied at 20 μM, while **7** showed slightly less inhibitory activity against iNOS expression. Therefore, the presence of multiple hydroxyl groups in the dibenzo-α-pyrone scaffold would be important for the observed inhibitory activity against iNOS expression, since the methylation of C-9 hydroxyl group abolishes the activity in **8**, while the additional C-2 hydroxyl group increases the activity of **6**. The fused α-pyran moiety into dibenzo-α-pyrone scaffold abolishes the inhibitory activity against iNOS expression activity in **5**.

No significant cytotoxicity of both compounds to RAW264.7 cells was observed under the test conditions, even up to 100 μM of **6** and **7** were used (Figures 4F,G). Solhaug et al. reported the cytotoxic activity of **7** in cells and we ascribe this difference as the shorter treatment period (18 h) in our study, compared to the previously used 48 h (Solhaug et al., 2015). These results suggest that the inhibitory activities of **6** and **7** against iNOS expression in LPS-stimulated RAW264.7 cells did not involve general cytotoxicity.

### 2-Hydroxy-alternariol (6) and Alternariol (7) Decreased the Protein Levels of TNF-α, IL-6 and MCP-1 in LPS-Stimulated RAW264.7 Cells

**FIGURE 5:**
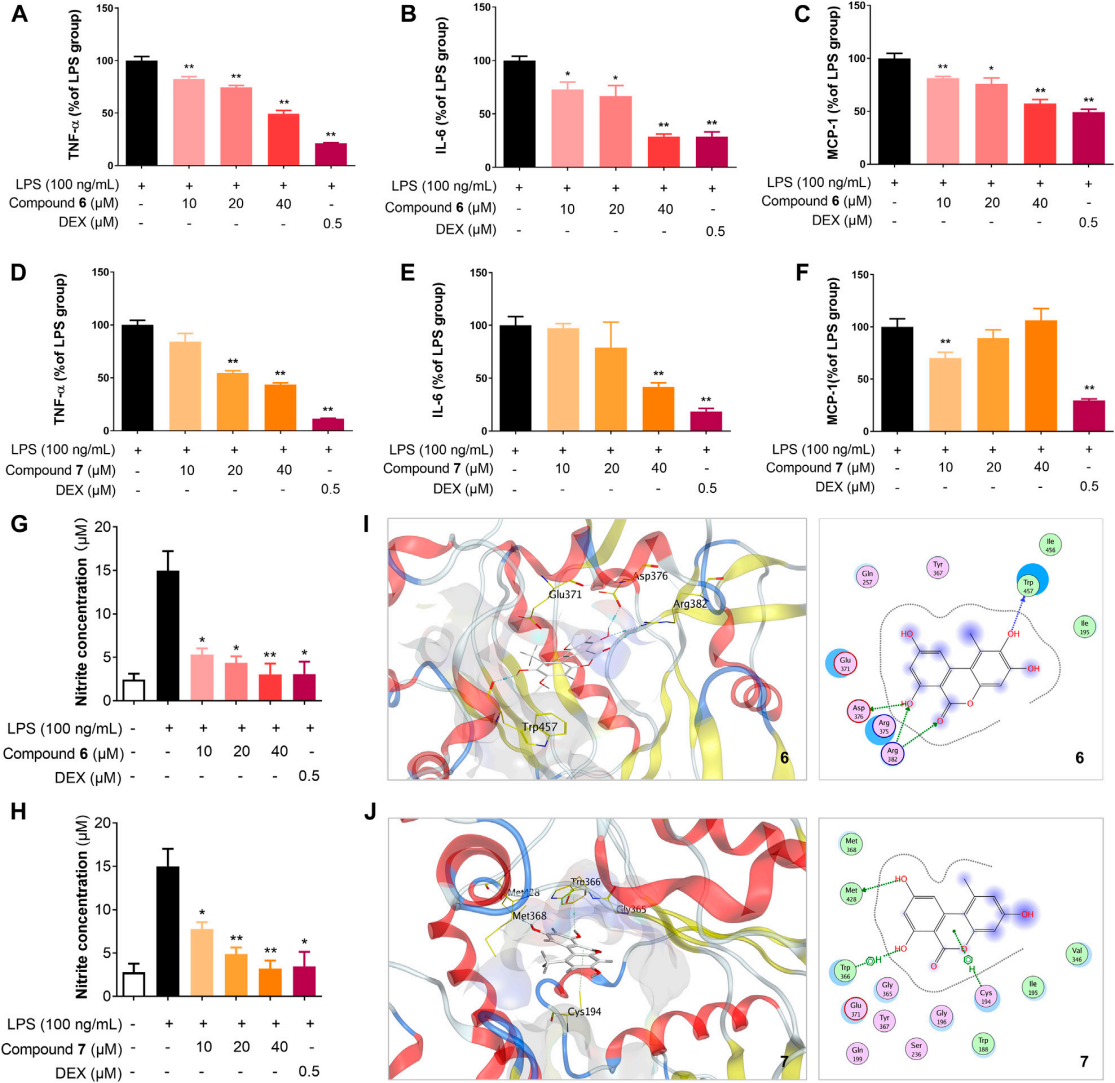
Compounds **6** and **7** inhibit the production of inflammatory cytokines and NO, probably through iNOS. Effects of **6 (A**–**C)** and **7 (D**–**F)** on the protein expression levels of TNF-α, IL-6, and MCP-1 in RAW264.7 cells. The cells were seeded in 12-well plates in a density of 2 × 10^5^ cells/mL, incubated for 18 h and their protein expression were analyzed using the ELISA kit (eBioscience). Effects of **6 (G)** and **7 (H)** on the production of NO in RAW264.7 cells. The cells were pretreated for 1 h with the indicated concentrations of **6** or **7** and then stimulated with LPS (100 ng/ml) for 24 h. NO production in the culture medium was detected using NO detection kit (Invitrogen). The data shown represent the mean values of three independent experiments. *p < 0.05, **p < 0.01. Molecular docking of **6 (I)** and **7 (J)** with iNOS (PDB ID: 3NW2) using MOE. For clarity, only interacting residues are labeled and hydrogen bonding interactions are shown by dashed arrows.

Macrophages are central effectors in inflammation, and activated macrophages act through the release of cytokines, such as tumor necrosis factor α (TNF-α) and interleukin-6 (IL-6), as well as chemokines, such as monocyte chemoattractant protein-1 (MCP-1) (Fujiwara and Kobayashi, 2005). Since TNF-α, IL-6, and MCP-1 are major mediators of inflammation, we examined the effects of **6** and **7** on LPS-induced TNF-α, IL-6, and MCP-1 production in RAW264.7 cells, using DEX as a control (Figures 5A–F). After RAW264.7 cells were stimulated with LPS (100 ng/ml) for 18 h in the presence or absence of **6** and **7**, the levels of TNF-α, IL-6, and MCP-1 in the culture media were measured by the ELISA. As shown in Figures 5A–C, compound **6** is able to inhibit the production of the tested cytokines and chemokines in a dose-dependent manner in RAW264.7 cells stimulated with LPS (*p* < 0.05). Significantly, the protein level of IL-6 is reduced to ∼25% by **6** (40 μM), which is comparable to the treatment by DEX (0.5 μM). Similar inhibitory effects of **7** could be observed for TNF-α and IL-6, while intriguingly, higher concentrations of **7**, e.g., 20 and 40 μM, have no obvious effects on MCP-1 production.

### Anti-Inflammatory Effects of 2-Hydroxy-Alternariol (6) and Alternariol (7) on LPS-Induced NO Production in LPS-Stimulated RAW264.7 Cells

NO, an important cellular messenger, may mediate various biological processes, including inflammation, induction of tumor cell death, and killing alien microorganisms (Jiang et al., 2021b). We next investigated the release of NO by the treatment of **6** and **7** in LPS-stimulated RAW264.7 cells (Figures 5G,H). The level of nitrite, a stable oxidized product of NO in the culture medium of RAW264.7 cells were measured using a nitric oxide detection kit. The treatment with **6** and **7** reduced LPS-induced NO production in a dose-dependent manner in the range of 10–40 μM. Compound **6** showed stronger inhibition of NO production than **7** in 10 μM, consistent with the more potent inhibition of iNOS by 6.

### Molecular Docking Simulations of 6 and 7 With iNOS

Due to the reduction of NO level and the reduced expression of iNOS by the treatment of **6** and **7** in LPS-treated RAW264.7 cells, compounds **6** and **7** may interact with iNOS and block the biosynthesis of NO. To our knowledge, the dibenzo-α-pyrone scaffold has not been used as iNOS inhibitors. Therefore, the interactions of **6** and **7** with iNOS were investigated using molecular docking by Molecular Operating Environment (MOE) (Hu et al., 2017; Jiang et al., 2021b). Using the crystal structure of mouse inducible nitric oxide synthase (miNOS, 3NW2) as a template, compound **6** form four hydrogen-bonding interactions with the amino acid residue Trp457, Asp376, and Arg382 of iNOS, while **7** only has one hydrogen-bonding interaction and two π-π interactions with Met428 (Figures 5I,J). Taken together, **6** and **7** may interact with iNOS and could be further developed as specific inhibitors against iNOS.

## Conclusion

In conclusion, five new compounds, including a new dibenzo-α-pyrone derivative, ellagic acid B (**5**) and four new α-pyrone diaporpyrone A–D (**9–12**), together with three known compounds (**6**–**8**), were isolated from the endophytic fungus *Diaporthe* sp. CB10100*.* Their structures were determined by the analyses of their NMR and HRESIMS spectra, while the absolute configuration of **10** and **11** was based on ECD spectra and their specific optical rotations. Ellagic acid B (**5**) features a tetracyclic 6/6/6/6 ring system with a fused 2*H*-chromene, while diaporpyrones consist of a pyrone and a short fatty acid side chain. The biosynthesis of **5** and **8** may involve a fungus PKS similar to SnPKS19 from *P. nodorum*, while the biosynthesis of **9**–**12** would likely be catalyzed by distinct fungi PKSs. All of the isolated compounds **5**–**12** were evaluated for their anti-inflammatory activities in LPS-stimulated RAW 264.7 macrophages. Among them, **6** and **7** display strong inhibitory activities against iNOS and inhibit nitric oxide production. In addition, both compounds significantly reduced the production of pro-inflammatory mediators and cytokines, including NO, TNF-α, IL-6, and MCP-1. Molecular docking of **6** and **7** to iNOS further suggests that they are potential iNOS inhibitors. Considering the importance of iNOS in inflammation and signal transduction, the discovery of **6** and **7** the alternariol scaffold may be further developed as potential iNOS inhibitors.

## Data Availability

The datasets presented in this study can be found in online repositories. The names of the repository/repositories and accession number(s) can be found in the article/Supplementary Material.
