# Supplementary material for: Bioactive α-Pyrone Derivatives from the Endophytic Fungus Diaporthe sp. CB10100 as Inducible Nitric Oxide Synthase Inhibitors
Source: Front Chem. 2021 May 18;9:679592. doi: 10.3389/fchem.2021.679592 (PMC8167431; doi:10.3389/fchem.2021.679592)
Supplement: Supplementary file 1 [file DataSheet1.PDF]

Supporting Information

Bioactive α-pyrone derivatives from the endophytic fungus *Diaporthe* sp. CB10100 as inducible nitric oxide synthase inhibitors

Hong Pu[1, 2, 3], Jianxin Liu[2,], Yeji Wang[1], Yuhui Peng[2], Wanying Zheng[2], Yang Tang[2], Boping Hui[2], Chunmei Nie[2], Xueshuang Huang[3], Yanwen Duan[1, 4, 5, *] and Yong Huang[1, 5, *]

[1]Xiangya International Academy of Translational Medicine, Central South University, Changsha, Hunan, 410013, China

[2]School of Pharmaceutical Sciences, Hunan University of Medicine, Huaihua, Hunan, 418000, China

[3]Hunan Provincial Key Laboratory for Synthetic Biology of Traditional Chinese Medicine，Hunan University of Medicine, Huaihua, Hunan, 418000, China

[4]Hunan Engineering Research Center of Combinatorial Biosynthesis and Natural Product Drug Discover, Changsha, Hunan, 410011, China

[5]National Engineering Research Center of Combinatorial Biosynthesis for Drug Discovery, Changsha, Hunan, 410011, China

***Corresponding author.**

E-mail address: jonghuang@csu.edu.cn (Y. Huang); ywduan66@sina.com (Y. Duan)

Table of Contents

Experimental Procedures 4
General experimental procedures 4
Isolation of endophytes 4
ECD calculations methods 5
Cell Culture and Cell viability assay 5
Western blotting 6
Molecular docking 6
Figure S1 Source of endophytes and inhibitory activity of iNOS and COX-2 protein produced by fermentation. **7**
Figure S2 Species identification of CB10100 (A) The ITS sequence data of *Diaporthe* sp. CB10100 (B) Phylogenetic tree analysis of *Diaporthe* sp. CB10100. 8
Figure S3 Some of the different types of bioactive metabolites were discovered from *Diaporthe* or *Phomopsis* species 9
Figure S4 UV spectrum of compounds **5** – **12** (A – H) 10
Figure S5 Optical Rotatory Dispersion (ORD) spectrum of compounds **10** – **11** (A – B) 14
Figure S6 HRESIMS spectrum of Compounds **5** – **12** (A – H) 15
Figure S7 ^{1}H NMR spectrum of **5** in DMSO-d_6 (500 MHz) 19
Figure S8 ^{13}C NMR spectrum of **5** in DMSO-d_6 (125 MHz) 20
Figure S9 DEPT-90 spectrum of **5** 21
Figure S10 DEPT-135 spectrum of **5** 22
Figure S11 HSQC spectrum of **5** 23
Figure S12 HMBC spectrum of **5** 24
Figure S13 ^{1}H-^{1}H COSY spectrum of **5** 25
Figure S14 ^{1}H NMR spectrum of **6** in DMSO-d_6 (500 MHz) 26
Figure S15 ^{13}C NMR spectrum of **6** in DMSO-d_6 (125MHz) 27
Figure S16 DEPT-90 spectrum of **6** 28
Figure S17 DEPT-135 spectrum of **6** 29
Figure S18 HSQC spectrum of **6** 30
Figure S19 HMBC spectrum of **6** 31
Figure S20 ^{1}H-^{1}H COSY spectrum of **6** 32
Figure S21 ^{1}H NMR spectrum of **7** in DMSO (400 MHz) 33
Figure S22 ^{13}C NMR spectrum of **7** in DMSO (100 MHz) 34
Figure S23 DEPT-90 spectrum of **7** 35
Figure S24 DEPT-135 spectrum of **7** 36
Figure S25 HSQC spectrum of **7** 37
Figure S26 HMBC spectrum of **7** 38
Figure S27 ^{1}H-^{1}H COSY spectrum of **7** 39
Figure S28 ^{1}H NMR spectrum of **8** in DMSO-d_6 (500 MHz) 40
Figure S29 ^{13}C NMR spectrum of **8** in DMSO-d_6 (125 MHz) 41
Figure S30 DEPT-90 spectrum of **8** 42
Figure S31 DEPT-135 spectrum of **8** 43
Figure S32 HSQC spectrum of **8** 44
Figure S33 HMBC spectrum of **8** 45

Figure S34 ^{1}H-^{1}H COSY spectrum of **8** 46
Figure S35 ^{1}H NMR spectrum of **9** in CD_3OD (400 MHz) 47
Figure S36 ^{13}C NMR spectrum of **9** in CD_3OD (100 MHz) 48
Figure S37 DEPT-90 spectrum of **9** 49
Figure S38 DEPT-135 spectrum of **9** 50
Figure S39 HSQC spectrum of **9** 51
Figure S40 HMBC spectrum of **9** 52
Figure S41 ^{1}H-^{1}H COSY spectrum of **9** 53
Figure S42 ^{1}H NMR spectrum of **9** in DMSO-d_6 (400 MHz) 54
Figure S43 ^{13}C NMR spectrum of **9** in DMSO-d_6 (100 MHz) 55
Figure S44 DEPT-90 spectrum of **9** 56
Figure S45 DEPT-135 spectrum of **9** 57
Figure S46 HSQC spectrum of **9** 58
Figure S47 HMBC spectrum of **9** 59
Figure S48 ^{1}H-^{1}H COSY spectrum of **9** 60
Figure S49 ^{1}H NMR spectrum of **10** in CD_3OD (500 MHz) 61
Figure S50 ^{13}C NMR spectrum of **10** in CD_3OD (125 MHz) 62
Figure S51 DEPT-90 spectrum of **10** 63
Figure S52 DEPT-135 spectrum of **10** 64
Figure S53 HSQC spectrum of **10** 65
Figure S54 HMBC spectrum of **10** 66
Figure S55 ^{1}H-^{1}H COSY spectrum of **10** 67
Figure S56 NOESY spectrum of **10** 68
Figure S57 ^{1}H NMR spectrum of **11** in CD_3OD (400 MHz) 69
Figure S58 ^{13}C NMR spectrum of **11** in CD_3OD (100 MHz) 70
Figure S59 DEPT-90 spectrum of **11** 71
Figure S60 DEPT-135 spectrum of **11** 72
Figure S61 HSQC spectrum of **11** 73
Figure S62 HMBC spectrum of **11** 74
Figure S63 ^{1}H-^{1}H COSY spectrum of **11** 75
Figure S64 NOESY spectrum of **11** 76
Figure S65 ^{1}H NMR spectrum of **12** in CD_3OD (500 MHz) 77
Figure S66 ^{13}C NMR spectrum of **12** in CD_3OD (125 MHz) 78
Figure S67 DEPT-90 spectrum of **12** 79
Figure S68 DEPT-135 spectrum of **12** 80
Figure S69 HSQC spectrum of **12** 81
Figure S70 HMBC spectrum of **12** 82
Figure S71 ^{1}H-^{1}H COSY spectrum of **12** 83
Figure S72 Energy lowest conformers and populations of Diaporpyrone B (**10**) R and optimized coordinates (method: B3LYP/6-31+G(d)) 84
Figure S73 Energy lowest conformers and populations of Diaporpyrone B (**10**) S and optimized coordinates (method: B3LYP/6-31+G(d)) 88

Experimental Procedures

General experimental procedures

CD spectra were recorded on J-815 from JASCO. UV spectra were measured on a Waters 2998 PDA Detector. Optical rotations were determined on a JASCO P-1020 digital polarimeter (Horiba, Tokyo, Japan). Semi-preparative reversed-phase high performance liquid chromatography (RP-HPLC) was performed using a Waters 1525 Binary HPLC pump equipped with a Waters 2489 UV/visible detector and using a Welch Ultimate AQ-C18 column (250 × 10 mm, 5 μm). HRESIMS spectra were recorded on a Q-Exactive Focus Orbitrap MS (Thermo Electron, Bremen, Germany) connected to the Thermo Scientific Dionex Ultimate 3000 RS (Thermo Fisher Scientific, California, USA). NMR spectra were acquired using a Bruker 400 MHz or 500 MHz spectrometer. The Chemical shifts in ^{1}H NMR and ^{13}C NMR spectra were referenced to the solvents for methanol-d_4 (δ_H 3.31 and δ_C 49.0), and DMSO-d_6 (δ_H 2.50 and δ_C 39.6). Column chromatography (CC) was carried out on silica gel (200–300 mesh, Yantai Jiangyou Silica Gel Development Co., Ltd., Yantai, China), RP-C18 (AAG12S50, YMC Co., Ltd.), and Sephadex LH-20 (GE Healthcare).

Dulbecco's modified Eagle's medium (DMEM), fetal bovine serum (FBS), and other tissue culture reagents were purchased from Gibco BRL Co. (Grand Island, NY, USA). Lipopolysaccharide (LPS, from *Escherichia* coli 055: B5) and DEX were obtained from Sigma Chemical Co. (St. Louis, MO, USA). iNOS and COX-2 antibodies were purchased from Cell Signaling Technology (Boston, MA, USA), and β-actin antibody was obtained from Santa Cruz Biotechnology (Santa Cruz, CA, USA). Goat anti-mouse IgG and goat anti-rabbit IgG secondary antibodies were purchased from Santa Cruz Biotechnology (Santa Cruz, CA, USA).

Isolation of endophytes

Disinfection of plant materials Fresh roots and stems of *Sinomenium acutum* were first rinsed with tap water after necrotic rhizomes were removed. Next, they were soaked in detergent (3 min) and rinsed with distilled water (20 min). After washed with sterile water (3 ×), the prepared stems or roots were cut into 5-8 mm sections in a biosafety hood. These plant sections were next soaked in 75% ethanol (1 min), washed with sterile water (3 ×), soaked in 15% sodium hypochlorite solution (15 min), washed with sterile water (4 ×).

Isolation of endophytic fungi The sterilized roots and stems of *Sinomenium acutum* were placed

on PDA medium and Madin's medium containing streptomycin (25 mg/L) in an incubator (28 ℃). The endophytes grown under these conditions would be picked and further analyzed.

Medium formulation

（1）PDA medium：PDB 24 g，Agar 20 g，Tap water 1000 mL，natural pH.

（2）Madin's medium：Peptone 5 g, Glucose 10 g, KH_2PO_4 1 g, $MgSO_4.7H_2O$ 0.5 g, Agar 20 g, Bengal red 0.03 g, Tap water 1000 mL, natural pH

（3）Fungal seed medium: Glucose 20 g, Sucrose 10 g, Soybean powder 2 g, Peptone 10 g, K_2HPO_4 0.3 g, PEG 2.5 g, $NaNO_3$ 3 g, $(NH_4)_2SO_4$ 3 g, Tap water 1000 mL, natural pH.

ECD calculations methods

A random conformational search was performed for diaporpyrone B (**10**) S and diaporpyrone B (**10**) R using the MMFF94 molecular mechanics force field after the energy minimization by SYBYL 2.0 software package, which yielded 20 conformers for diaporpyrone B (**10**) R and 25 for diaporpyrone B (**10**) S respectively, within a 10 kcal/mol energy window. The following geometry optimizations at the B3LYP/6-31+G(d) level afforded 4 conformers for diaporpyrone B (**10**) R (Fig. S72) and 4 for diaporpyrone B (**10**) S (Fig. S73) respectively, within a 10 kcal/mol energy window. Subsequently, the conformers were collected and submitted to ECD calculations by time-dependent density functional theory (TDDFT) calculations at the B3LYP/6-31+G(d) level, considering the lowest 100 excited states with the CPCM solvent model for methanol. The resulting ECD spectra of all conformers were energetically weighted according to the respective conformational distribution by the Boltzmann statistics. The comparison of the experimental ECD spectrum of diaporpyrone B (**10**) with the calculated ones revealed that the Cotton effects (CEs) of diaporpyrone B (**10**) R were in good accordance with the experimental CEs of diaporpyrone B (**10**) in the region 200~400 nm.

Cell Culture and Cell viability assay

RAW 264.7 cells were cultivated in 100 mm dishes (5×10^6 cells/dish) in RPMI 1640 media with fetal bovine serum, penicillin G (100 U/mL), streptomycin (100 μg/mL), and L-glutamine (2 mM) and then incubated at 37 °C and 5% CO_2 in a humid environment. The 2-hydroxy-alternariol (**6**) and alternariol (**7**) was added to the cells and incubated for 1 h and then cells were treated with or without LPS for 18 h at 37 ℃ with 5% CO_2. MTT solution (5 g/L) was added to each well and incubated for 4 h at 37 ℃. The formazan dyes in the cells were dissolved in 100 μL 10%

SDS-HCl solution. The optical density was read at 570 nm (reference, 650 nm) using a microplate UV/VIS spectrophotometer (Tecan, Mannedorf, Switzerland). The cell viability in the control group (cells were not treated by compounds and LPS) was set as 100%.

Western blotting

The proteins of iNOS and COX-2 were measured by Western blot analysis. The details of the procedures for the Western blot analysis were described previously. The cells were lysed with 20 mM Tris HCl buffer (pH 7.4) containing a protease inhibitor mixture (0.1 mM phenylmethylsulfonyl fluoride, 5 mg/mL aprotinin, 5 mg/mL pepstatin A, and 1 mg/mL chymostatin). The protein concentration was determined using a Lowry protein assay kit (P5626; Sigma-Aldrich, St. Louis, MO, USA). An equal amount of protein for each sample was resolved by performing 7.5% and 12% sodium dodecyl sulfatepolyacrylamide gel electrophoresis. The proteins were electrophoretically transferred onto Hybond enhanced chemiluminescence (ECL) nitrocellulose membranes (Bio-Rad), which were blocked with 5% skim milk and sequentially incubated with particular primary antibodies (Santa Cruz Biotechnology) and horseradish peroxidase-conjugated secondary antibodies, followed by ECL detection (Amersham Pharmacia Biotech, Piscataway, NJ, USA).

Molecular docking

The molecular modeling studies were carried out on an Intel core i5 2.5 GHz processor, 4 GB memory with the Windows 10 operating system using Molecular Operating Environment (MOE 2010.06; Chemical Computing Group, Canada) as the computational platform. All the energy minimizations were performed with MOE until a root-mean-square deviation gradient of 0.05 kcal mol−1 Å−1 with MMFF94X force-field and the partial charges were automatically calculated. The X-ray crystallographic structure of iNOS (PDB: 3NW2) was obtained from the RCSB Protein Data Bank.

Figure S1 Source of endophytes and inhibitory activity of iNOS and COX-2 protein produced by fermentation. (**A-1**) Map of Hunan province (**A-2**) The whole plant of *Sinomenium acutum* (**A-3**) Sinomenine, an alkaloid natural product, from *Sinomenium acutum*; (B) Endophytes isolated from *Sinomenium acutum* (**B-1**) CB10098 (**B-2**) CB10099 (**B-3**) CB10100 (**B-4**) CB10101 (**B-5**) CB10102 (**B-6**) CB10103. (**C**, **D**) Inhibitory effects of fermented crude extract of each strain C1-C6 on LPS-induced iNOS and COX-2 protein expression in RAW264.7 macrophages.

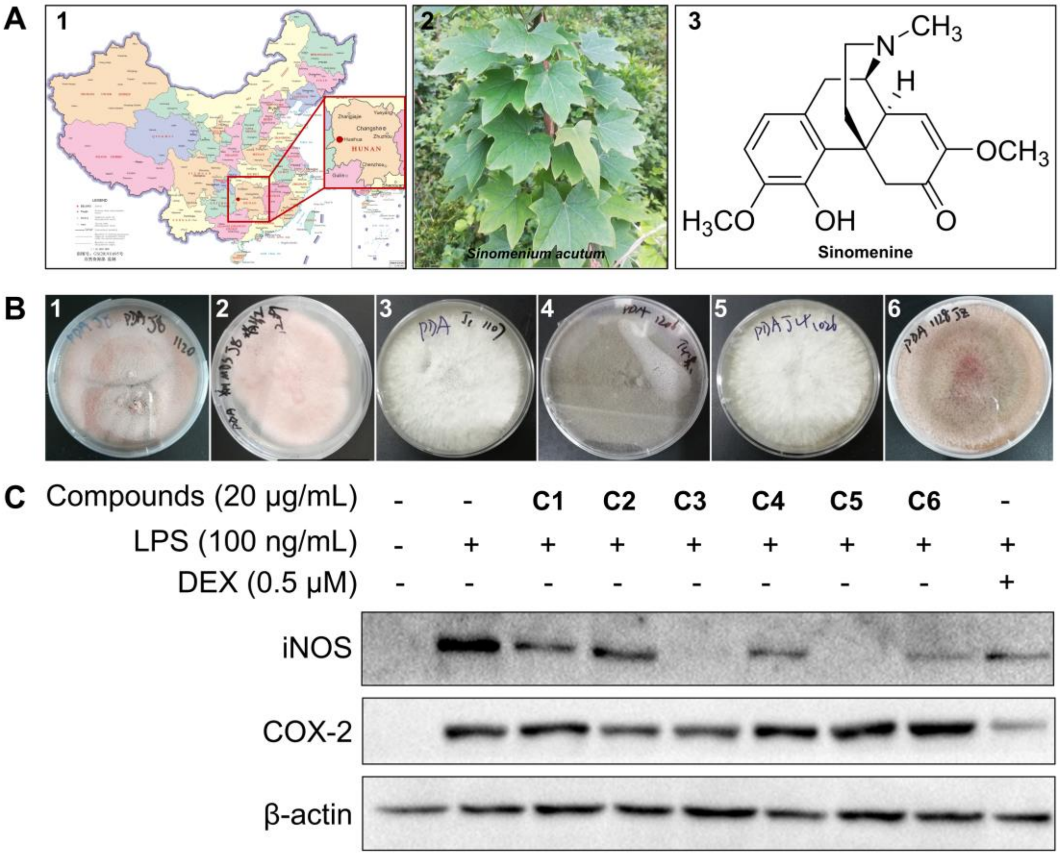

Figure S2 Species identification of CB10100 (A) The ITS sequence data of *Diaporthe* sp. CB10100 (B) Phylogenetic tree analysis of *Diaporthe* sp. CB10100.

A

TGCGGAGGGATCATTGCTGGAACGCGCCCCAGGCGCACCCAGAAACCCTTTGTGAACT

TATACCTTACTGTTGCCTCGGCGCTAGCTGGTCCTCCGGGGCCCCTCTCTCCGGAGGAG

CAGGCACGCCGGCGGCCAAGTTAACTCTTGTTTTTACACTGAAACTCTGAGGAATAAA

ACATAAATGAATCAAAACTTTCAACAACGGATCTCTTGGTTCTGGCATCGATGAAGAAC

GCAGCGAAATGCGATAAGTAATGTGAATTGCAGAATTCAGTGAATCATCGAATCTTTGA

ACGCACATTGCGCCCTCTGGTATTCCGGAGGGCATGCCTGTTCGAGCGTCATTTCAACC

CTCAAGCCTGGCTTGGTGATGGGGCACTGCCTGTAAAAGGGCAGGCCCTGAAATTCAG

TGGCGAGCTCGCCAGGACCCCGAGCGCAGTAGTTAAACCCTCGCTCTGGAAGGCCCT

GGCGGTGCCCTGCCGTTAAACCCCCAACTTCTGAAAATTTGACCTCGGATCAGGTAGG

AATACCCGCTGAACTTAAGCATAT

B

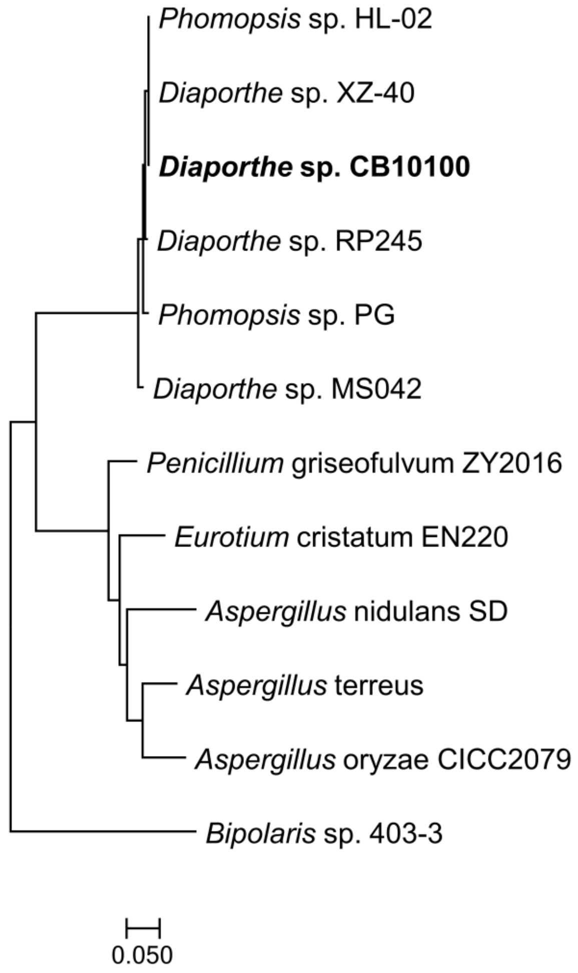

Figure S3 Some of the different types of bioactive metabolites were discovered from *Diaporthe* or *Phomopsis* species

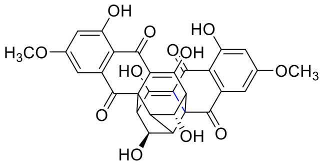

Cytoskyrin A (**13**)

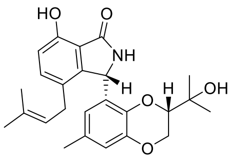

Diaporisoindoles A (**14**)

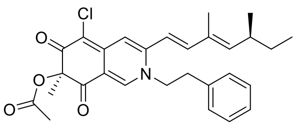

Isochromophilones X (**15**)

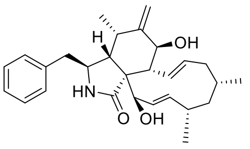

Diaporthichalasins A (**16**)

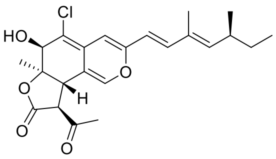

Isochromophilones A (**17**)

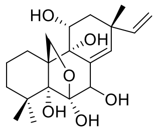

Diaporthein A (**18**)

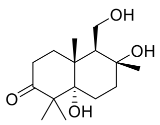

Diaporols B (**19**)

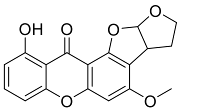

Asperxanthone D (**20**)

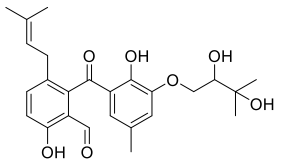

Tenellones A (**21**)

Figure S4 UV spectrum of compounds **5** – **12** (A – H)

A (**5**)

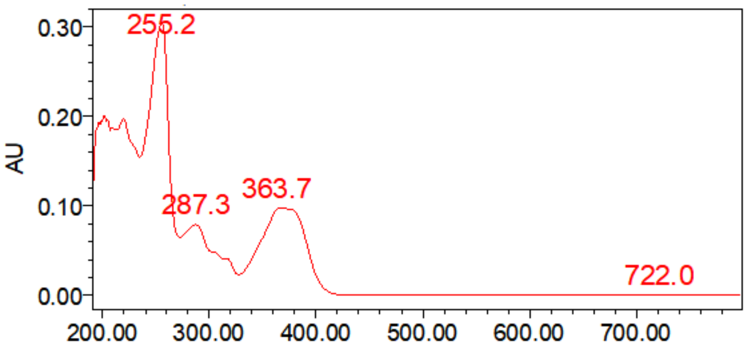

No.	Wavelength (nm)	Abs
1	200.9	0.202
2	255.2	0.305
3	287.2	0.079
4	363.7	0.097

B (**6**)

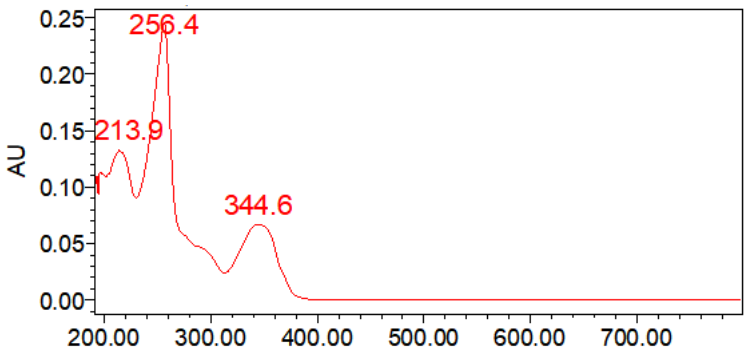

No.	Wavelength (nm)	Abs
1	213.9	0.133
2	256.4	0.246
3	344.6	0.067

C (**7**)

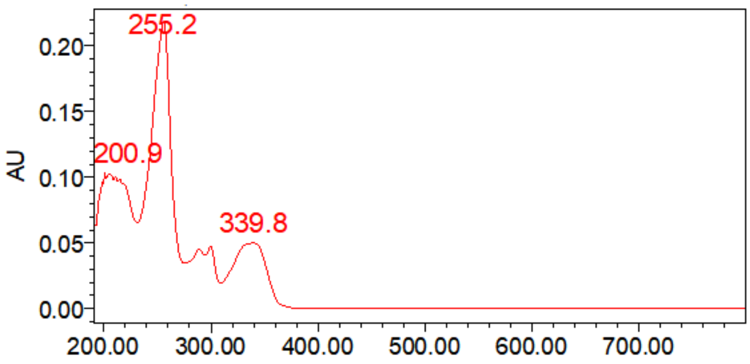

No.	Wavelength (nm)	Abs
1	200.9	0.103
2	255.2	0.217
3	299.2	0.048
4	339.8	0.05

D (**8**)

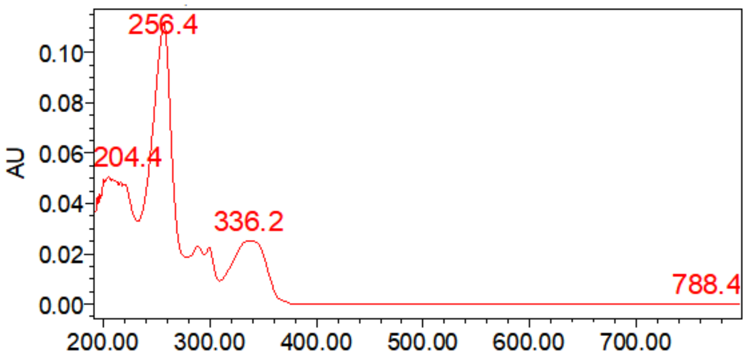

No.	Wavelength (nm)	Abs
1	204.5	0.051
2	256.4	0.111
3	288.4	0.023
4	336.2	0.0251

E (**9**)

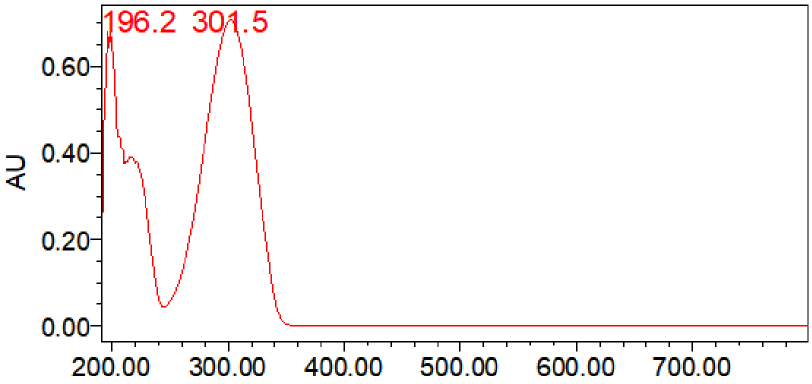

No.	Wavelength (nm)	Abs
1	196.2	0.682
2	301.5	0.707

F (**10**)

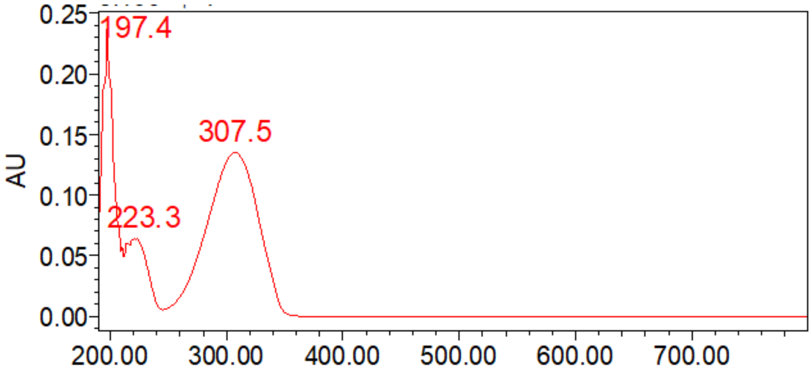

No.	Wavelength (nm)	Abs
1	197.4	0.239
2	223.3	0.065
3	307.5	0.135

G (**11**)

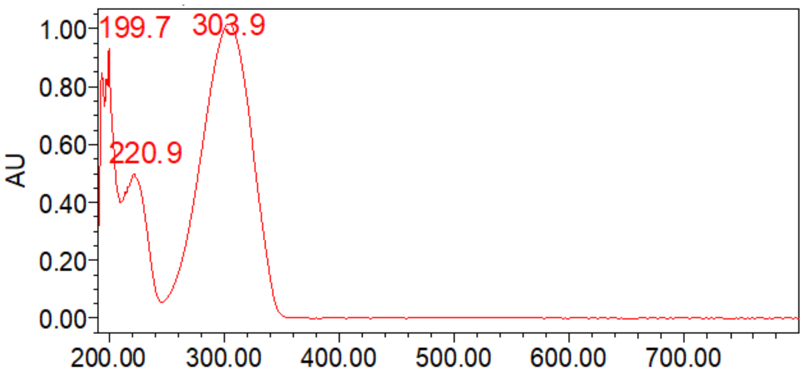

No.	Wavelength (nm)	Abs
1	199.7	0.932
2	220.9	0.5
3	303.9	1.017

H (**12**)

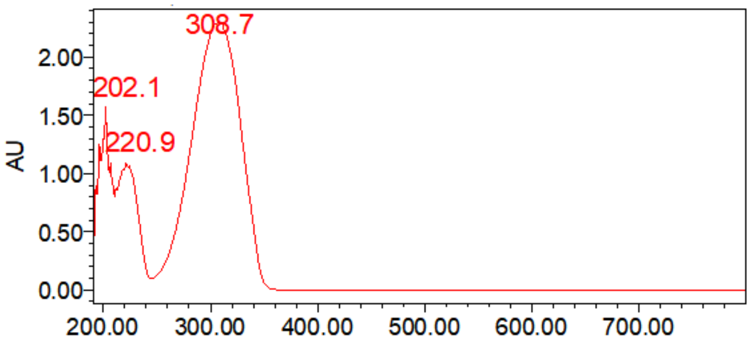

No.	Wavelength (nm)	Abs
1	202.1	1.57
2	220.9	1.097
3	308.7	2.3

Figure S5 Optical Rotatory Dispersion (ORD) spectrum of compounds **10** – **11** (A – B)

A (10)

Rudolph Research Analytical

This sample was measured on an Autopol VI, Serial #91058
Manufactured by Rudolph Research Analytical, Hackettstown, NJ, USA.

Measurement Date : Friday, 19-JUN-2020

Set Temperature : OFF

Time Delay : Disabled

Delay between Measurement : Disabled

n	Average	Std.Dev.	% RSD	Maximum	Minimum
5	1.92	1.07	55.72	2.97	0.25

S.No	Sample ID	Time	Result	Scale	OR °Arc	WLG.nm	Lg.mm	Conc.g/100ml	Temp.
1	PH39	03:46:27 PM	0.25	SR	0.0003	589	100.00	0.118	23.6
2	PH39	03:46:35 PM	2.54	SR	0.0030	589	100.00	0.118	23.6
3	PH39	03:46:43 PM	2.97	SR	0.0035	589	100.00	0.118	23.6
4	PH39	03:46:51 PM	1.53	SR	0.0018	589	100.00	0.118	23.6
5	PH39	03:46:59 PM	2.29	SR	0.0027	589	100.00	0.118	23.6

B (**11**)

Rudolph Research Analytical

This sample was measured on an Autopol VI, Serial #91058
Manufactured by Rudolph Research Analytical, Hackettstown, NJ, USA.

Measurement Date : Friday, 19-JUN-2020

Set Temperature : OFF

Time Delay : Disabled

Delay between Measurement : Disabled

n	Average	Std.Dev.	% RSD	Maximum	Minimum
5	14.72	1.86	12.63	16.67	12.50

S.No	Sample ID	Time	Result	Scale	OR °Arc	WLG.nm	Lg.mm	Conc.g/100ml	Temp.
1	PH49	03:56:51 PM	16.67	SR	0.012	589	100.00	0.072	23.6
2	PH49	03:56:57 PM	16.67	SR	0.012	589	100.00	0.072	23.6
3	PH49	03:57:03 PM	13.89	SR	0.010	589	100.00	0.072	23.6
4	PH49	03:57:08 PM	13.89	SR	0.010	589	100.00	0.072	23.6
5	PH49	03:57:14 PM	12.50	SR	0.009	589	100.00	0.072	23.6

Figure S6 HRESIMS spectrum of Compounds **5** – **12** (A – H)

A (**5**)

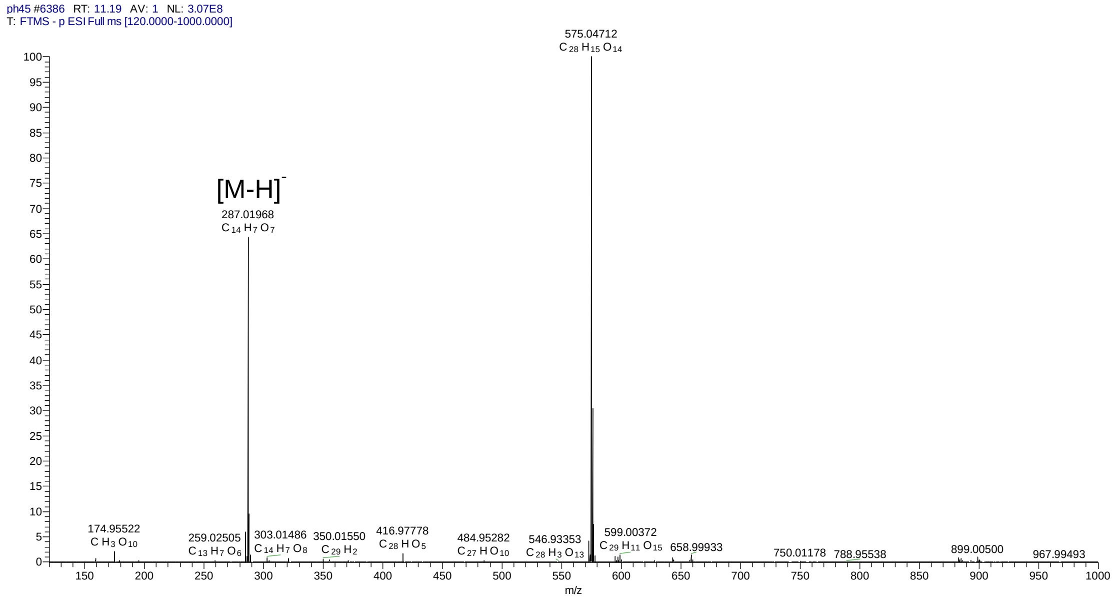

B (**6**)

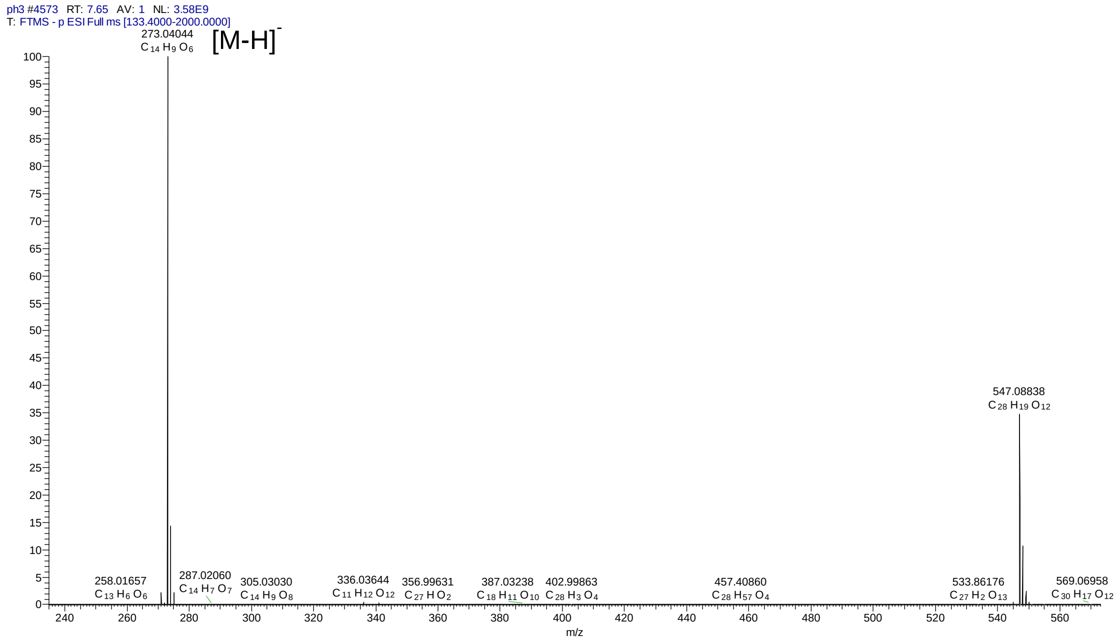

C (**7**)

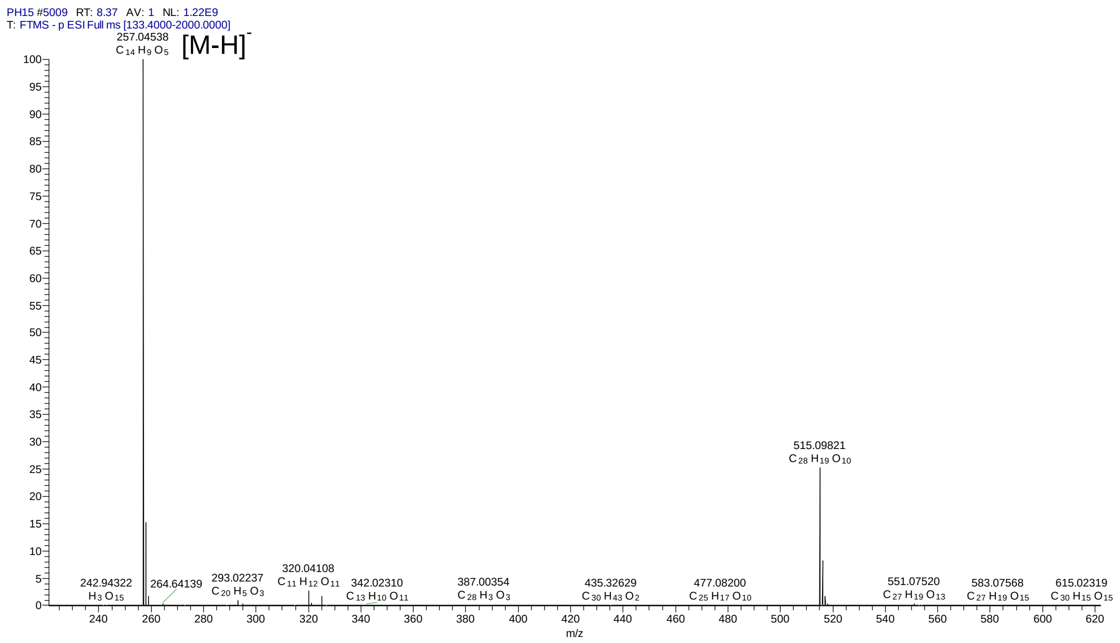
PH15 #5009 RT: 8.37 AV: 1 NL: 1.22E9
T: FTMS - p ESI Full ms [133.4000-2000.0000]
257.04538
C 14 H 9 O 5
[M-H]-
515.09821
C 28 H 19 O 10
242.94322
H 3 O 15
264.64139
293.02237
C 20 H 5 O 3
320.04108
C 11 H 12 O 11
342.02310
C 13 H 10 O 11
387.00354
C 28 H 3 O 3
435.32629
C 30 H 43 O 2
477.08200
C 25 H 17 O 10
551.07520
C 27 H 19 O 13
583.07568
C 27 H 19 O 15
615.02319
C 30 H 15 O 15
m/z

D (**8**)

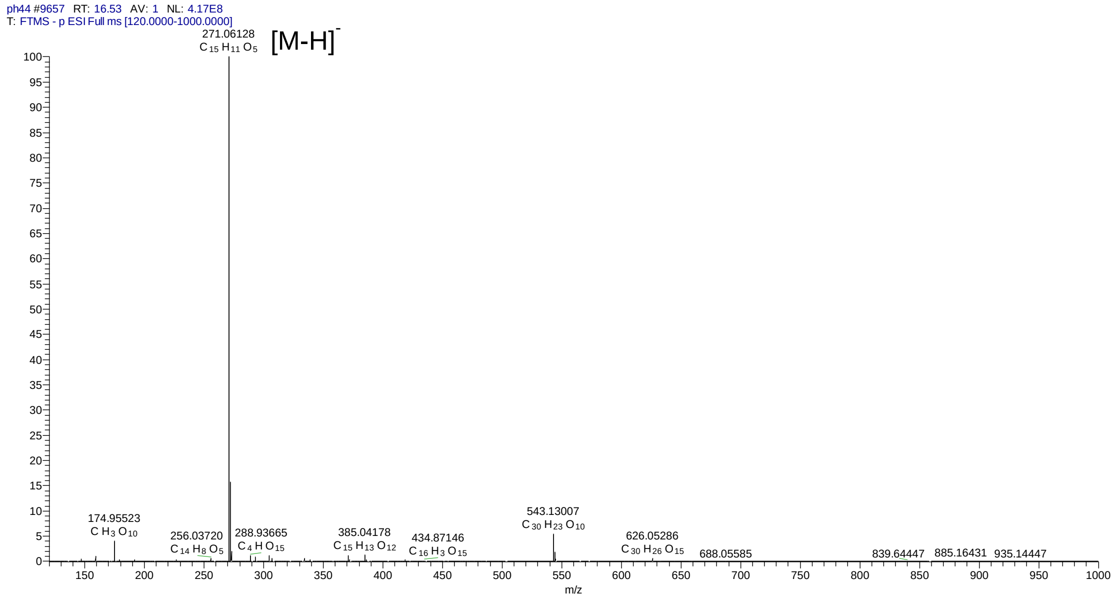
ph44 #9657 RT: 16.53 AV: 1 NL: 4.17E8
T: FTMS - p ESI Full ms [120.0000-1000.0000]
271.06128
C 15 H 11 O 5
[M-H]-
174.95523
C H 3 O 10
256.03720
C 14 H 8 O 5
288.93665
C 4 H O 15
385.04178
C 15 H 13 O 12
434.87146
C 16 H 3 O 15
543.13007
C 30 H 23 O 10
626.05286
C 30 H 26 O 15
688.05585
839.64447
885.16431
935.14447
m/z

E (**9**)

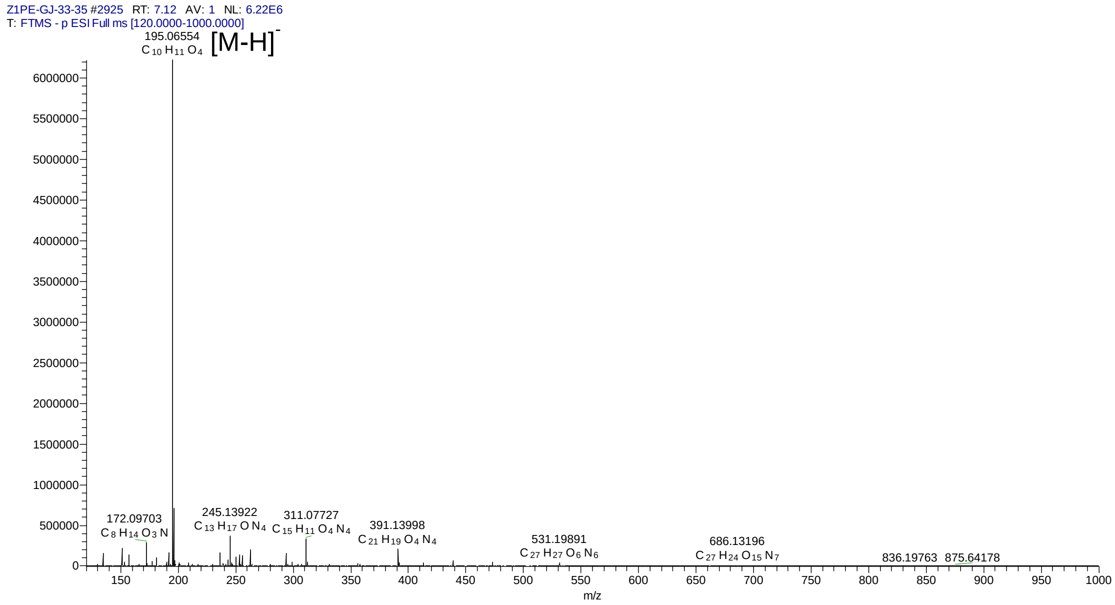

Z1PE-GJ-33-35 #2925 RT: 7.12 AV: 1 NL: 6.22E6
T: FTMS - p ESI Full ms [120.0000-1000.0000]
195.06554
C 10 H 11 O 4
[M-H]-
172.09703
C 8 H 14 O 3 N
245.13922
C 13 H 17 O N 4
311.07727
C 15 H 11 O 4 N 4
391.13998
C 21 H 19 O 4 N 4
531.19891
C 27 H 27 O 6 N 6
686.13196
C 27 H 24 O 15 N 7
836.19763
875.64178
m/z

F (**10**)

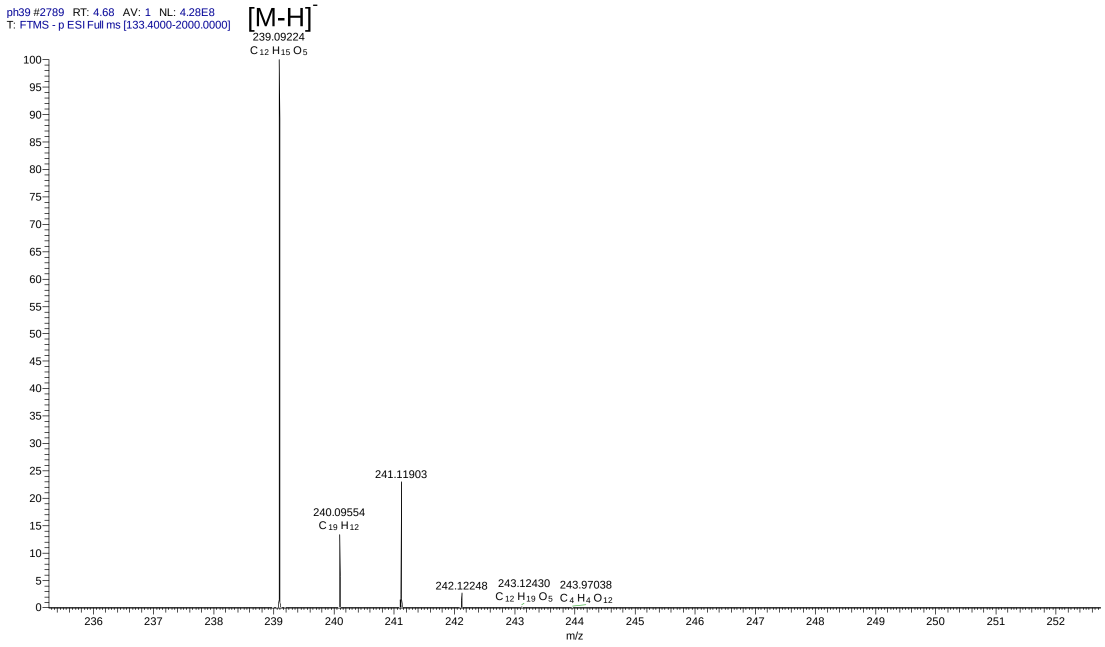

ph39 #2789 RT: 4.68 AV: 1 NL: 4.28E8
T: FTMS - p ESI Full ms [133.4000-2000.0000]
[M-H]-
239.09224
C 12 H 15 O 5
240.09554
C 19 H 12
241.11903
242.12248
243.12430
C 12 H 19 O 5
243.97038
C 4 H 4 O 12
m/z

G (**11**)

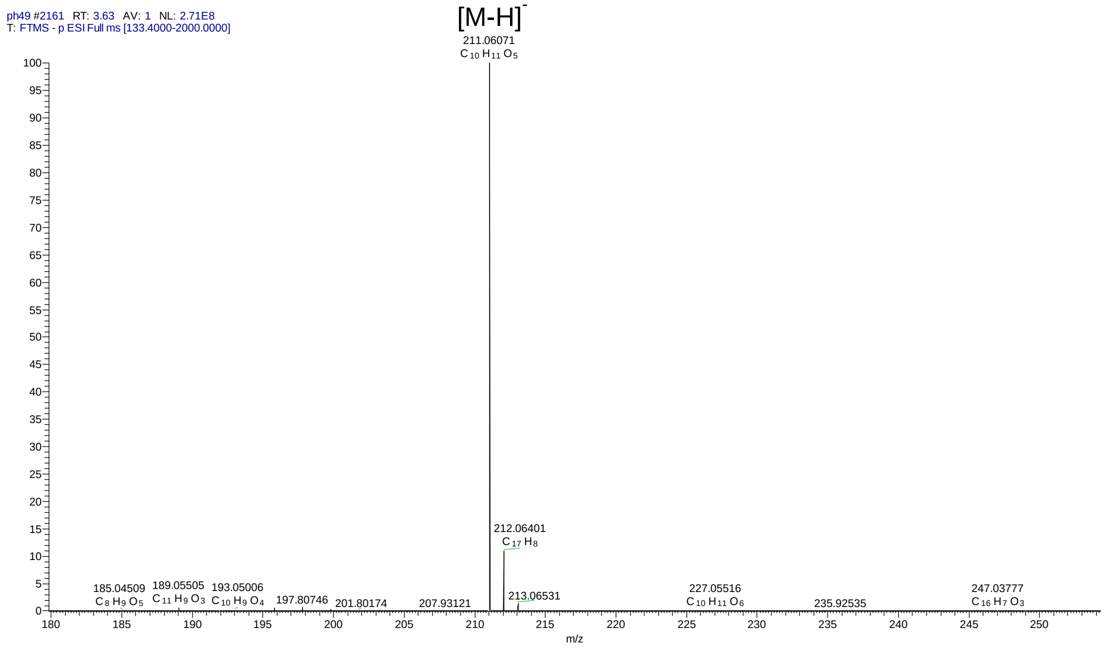
ph49 #2161 RT: 3.63 AV: 1 NL: 2.71E8
T: FTMS - p ESI Full ms [133.4000-2000.0000]
[M-H]-
211.06071
C 10 H 11 O 5
212.06401
C 17 H 8
213.06531
185.04509
C 8 H 9 O 5
189.05505
C 11 H 9 O 3
193.05006
C 10 H 9 O 4
197.80746
201.80174
207.93121
227.05516
C 10 H 11 O 6
235.92535
247.03777
C 16 H 7 O 3
m/z

H (**12**)

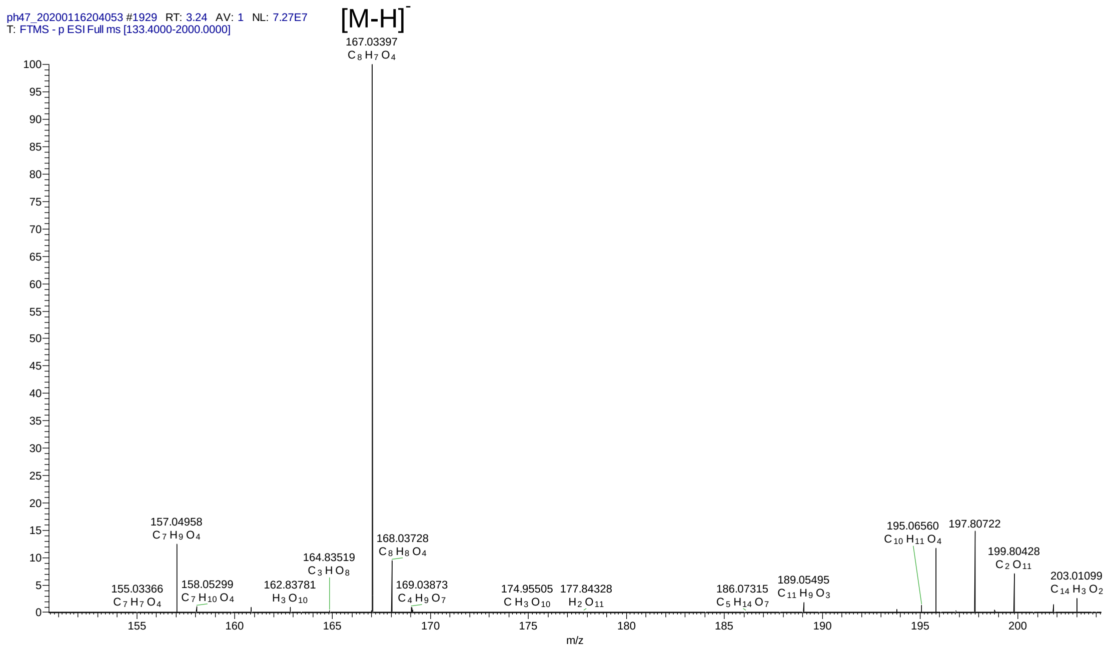
ph47_20200116204053 #1929 RT: 3.24 AV: 1 NL: 7.27E7
T: FTMS - p ESI Full ms [133.4000-2000.0000]
[M-H]-
167.03397
C 8 H 7 O 4
157.04958
C 7 H 9 O 4
155.03366
C 7 H 7 O 4
158.05299
C 7 H 10 O 4
162.83781
H 3 O 10
164.83519
C 3 H O 8
168.03728
C 8 H 8 O 4
169.03873
C 4 H 9 O 7
174.95505
C H 3 O 10
177.84328
H 2 O 11
186.07315
C 5 H 14 O 7
189.05495
C 11 H 9 O 3
195.06560
C 10 H 11 O 4
197.80722
199.80428
C 2 O 11
203.01099
C 14 H 3 O 2
m/z

Figure S7 ^{1}H NMR spectrum of **5** in DMSO-d_6 (500 MHz)

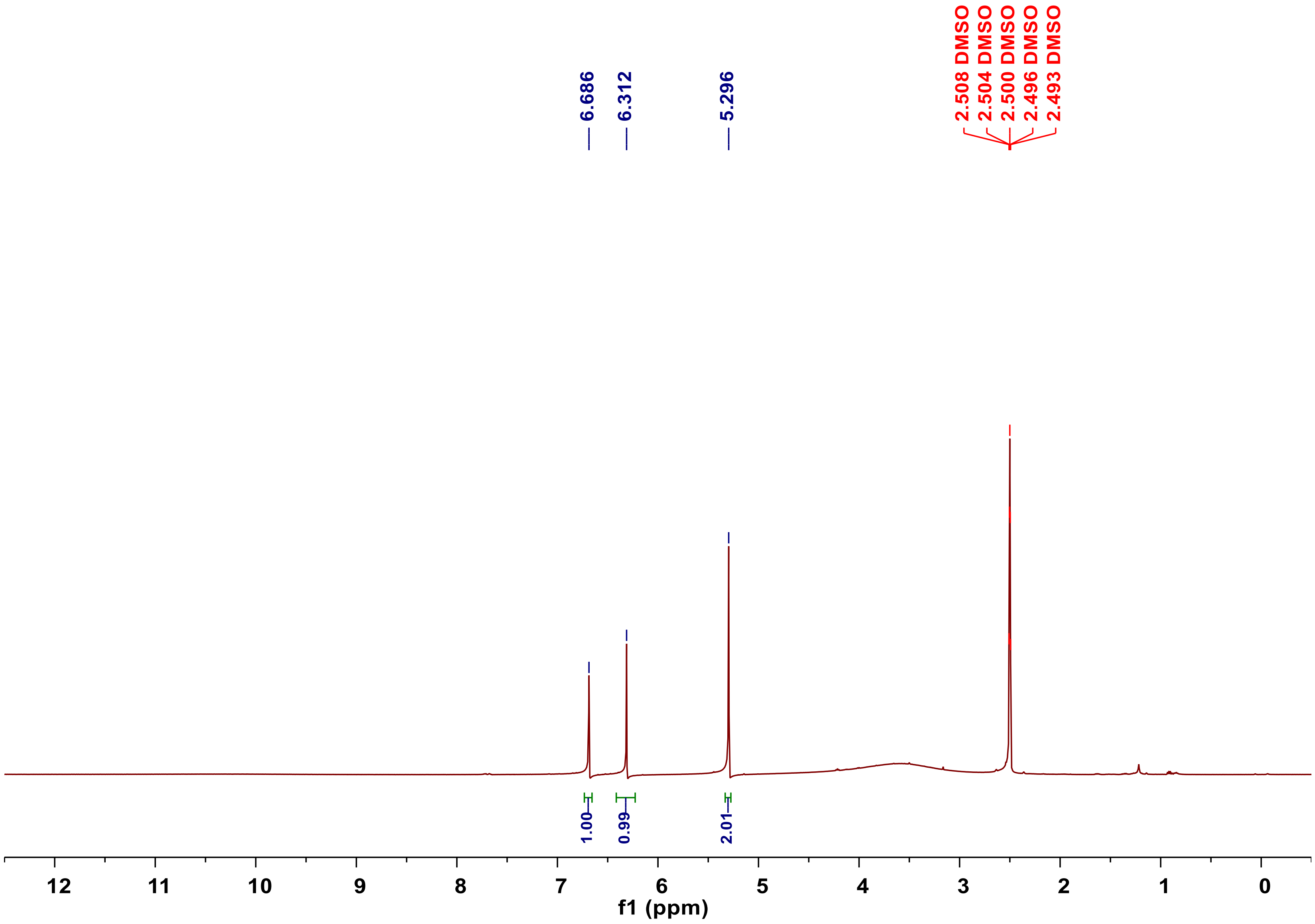

Figure S8 ^{13}C NMR spectrum of **5** in DMSO-d_6 (125 MHz)

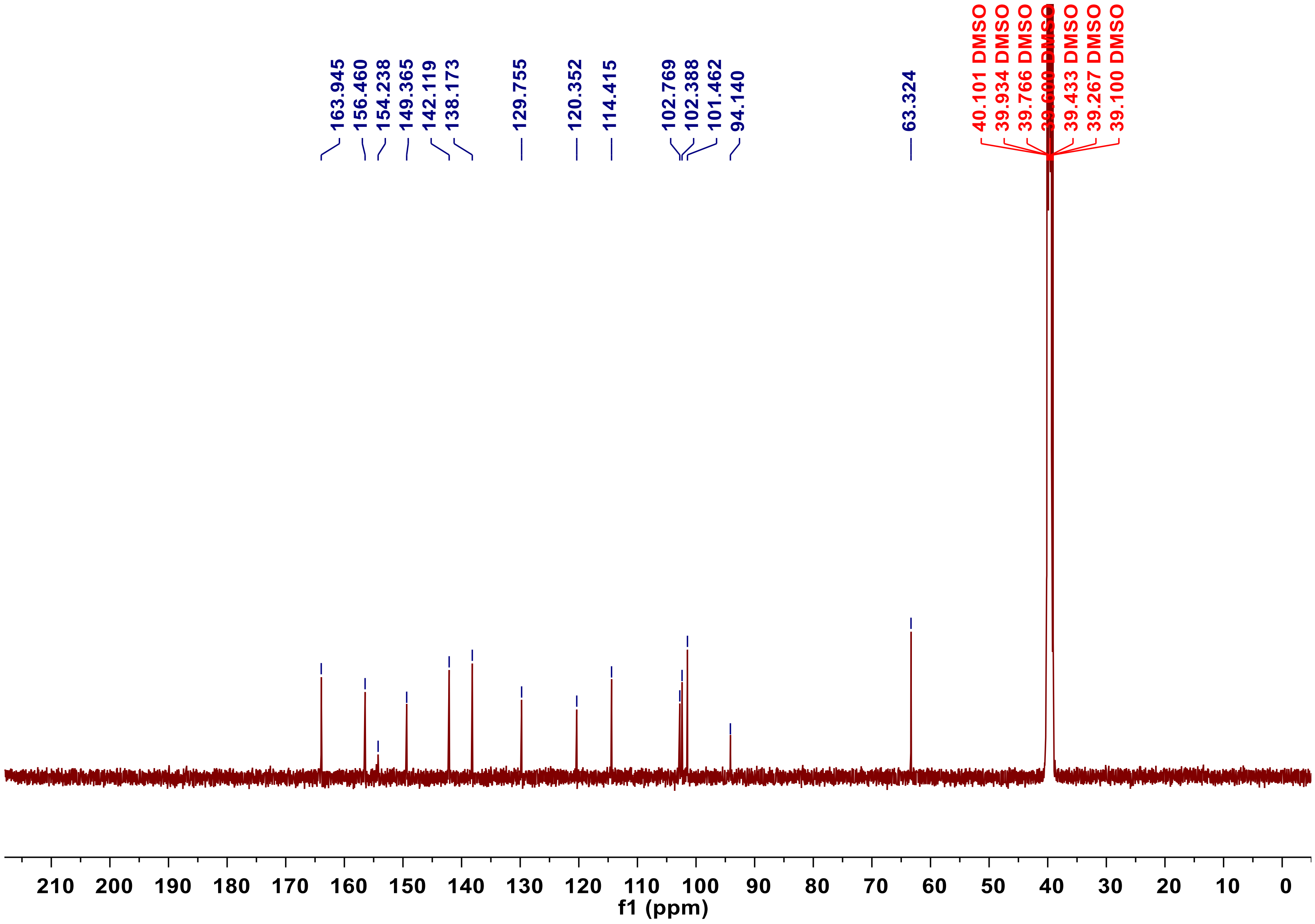

Figure S9 DEPT-90 spectrum of **5**

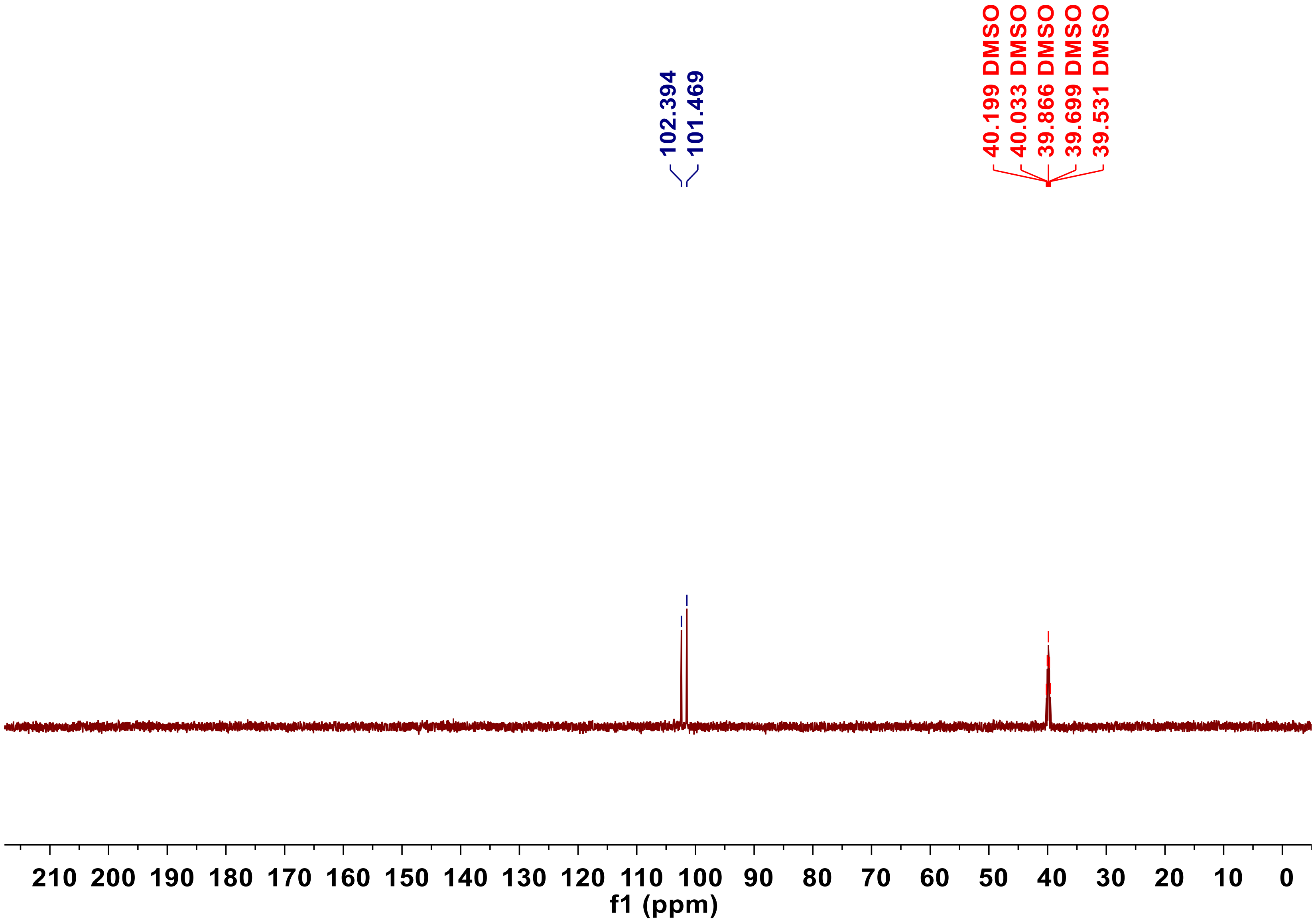

Figure S10 DEPT-135 spectrum of **5**

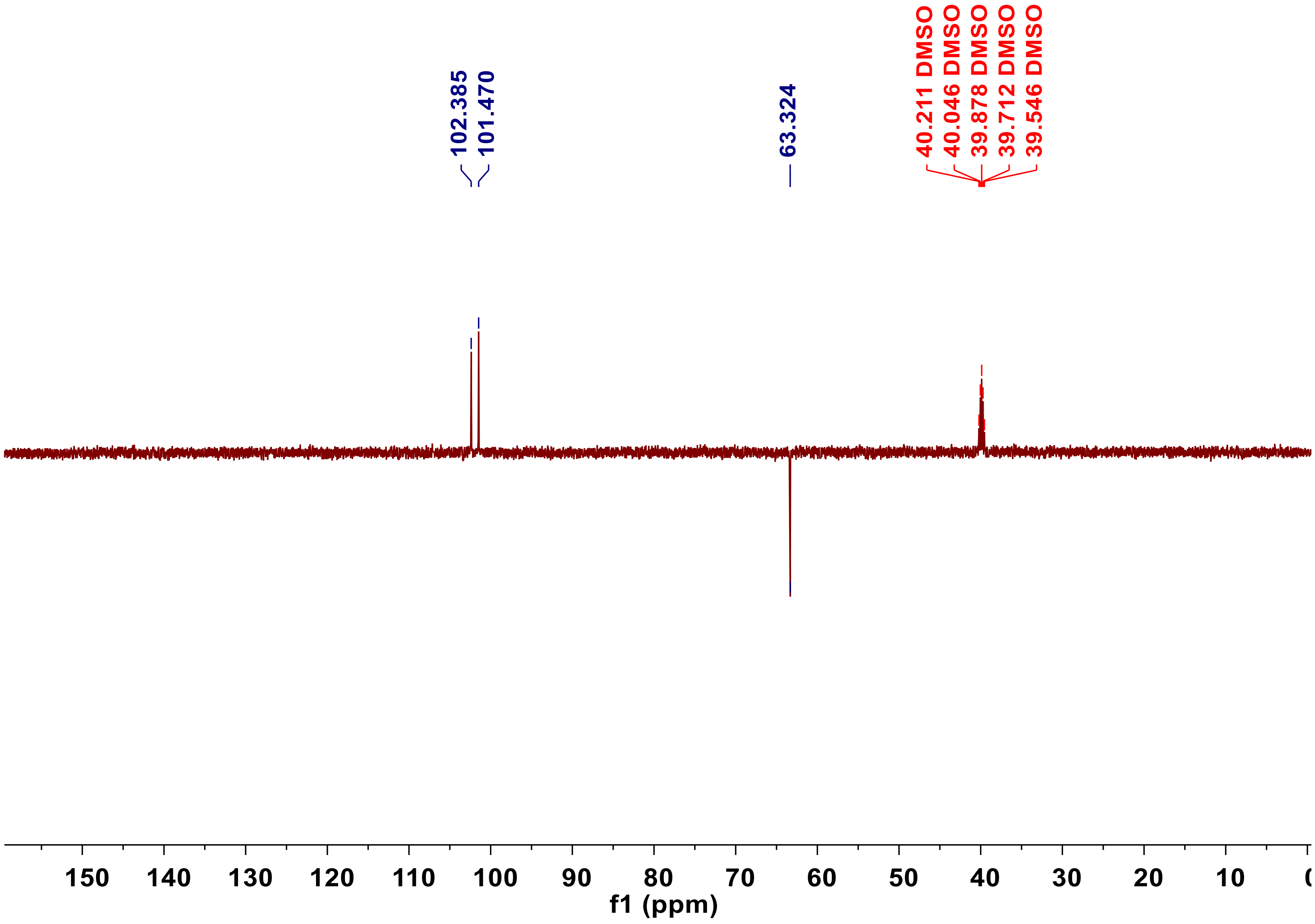

Figure S11 HSQC spectrum of **5**

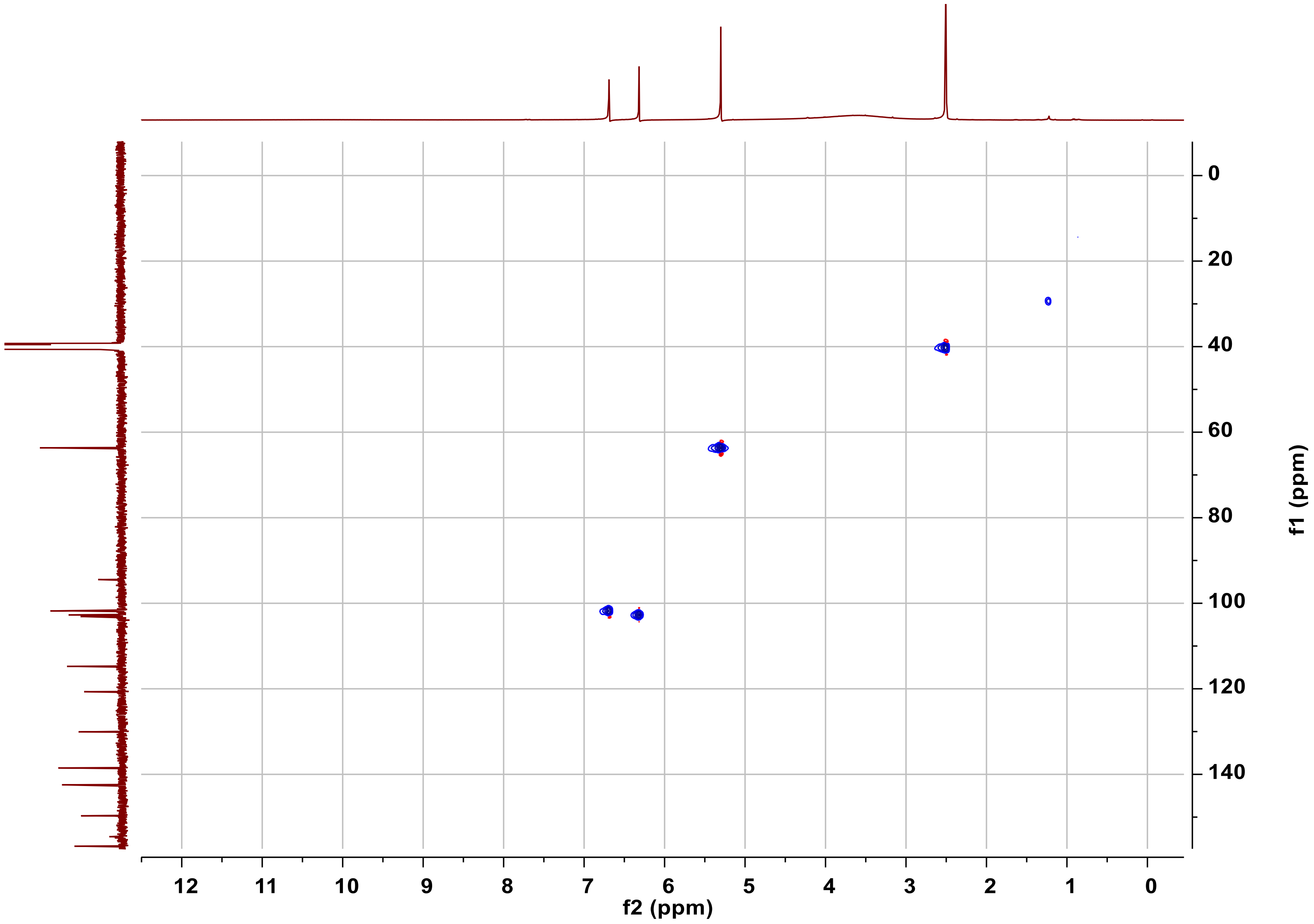

Figure S12 HMBC spectrum of **5**

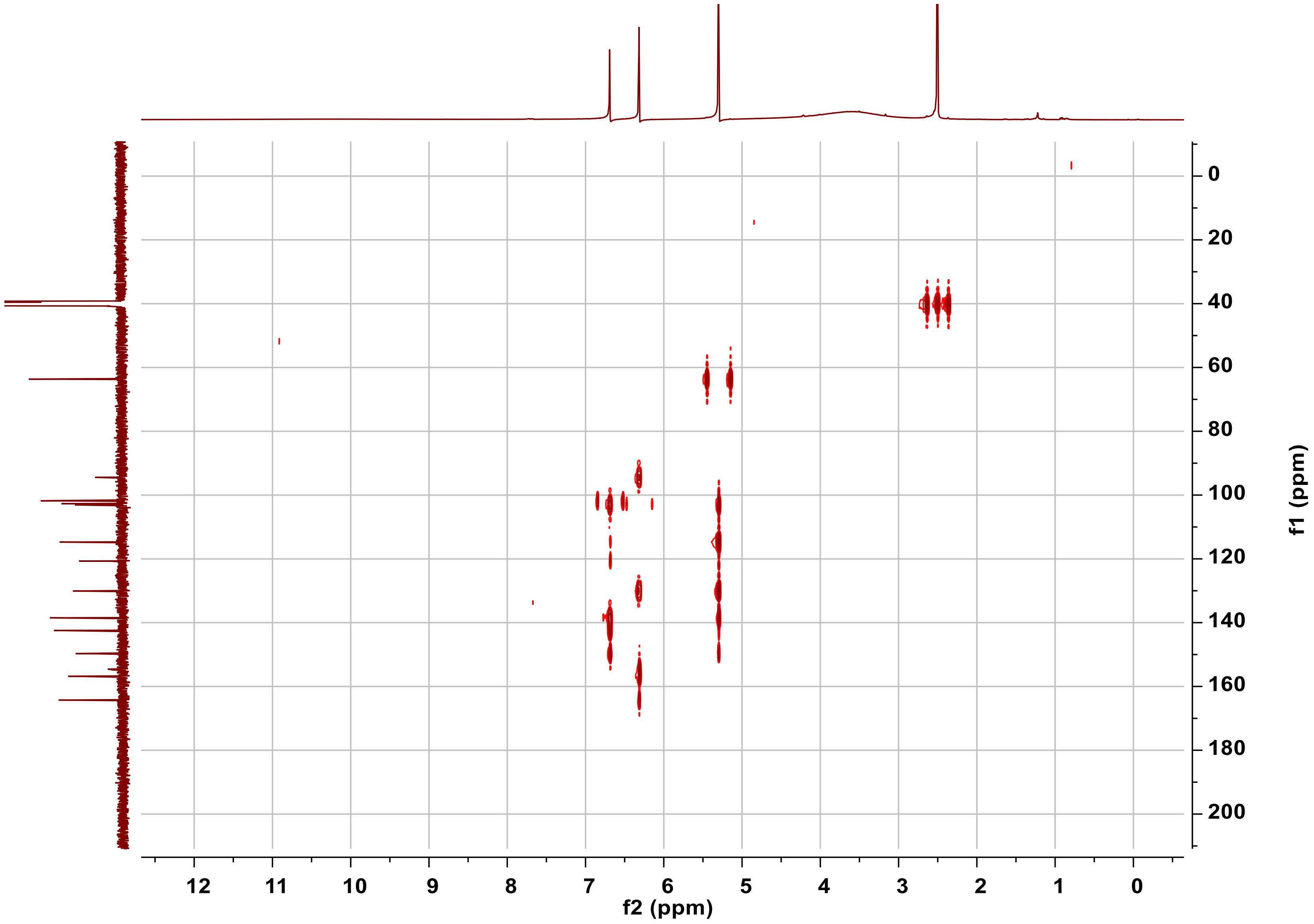

Figure S13 ^{1}H-^{1}H COSY spectrum of **5**

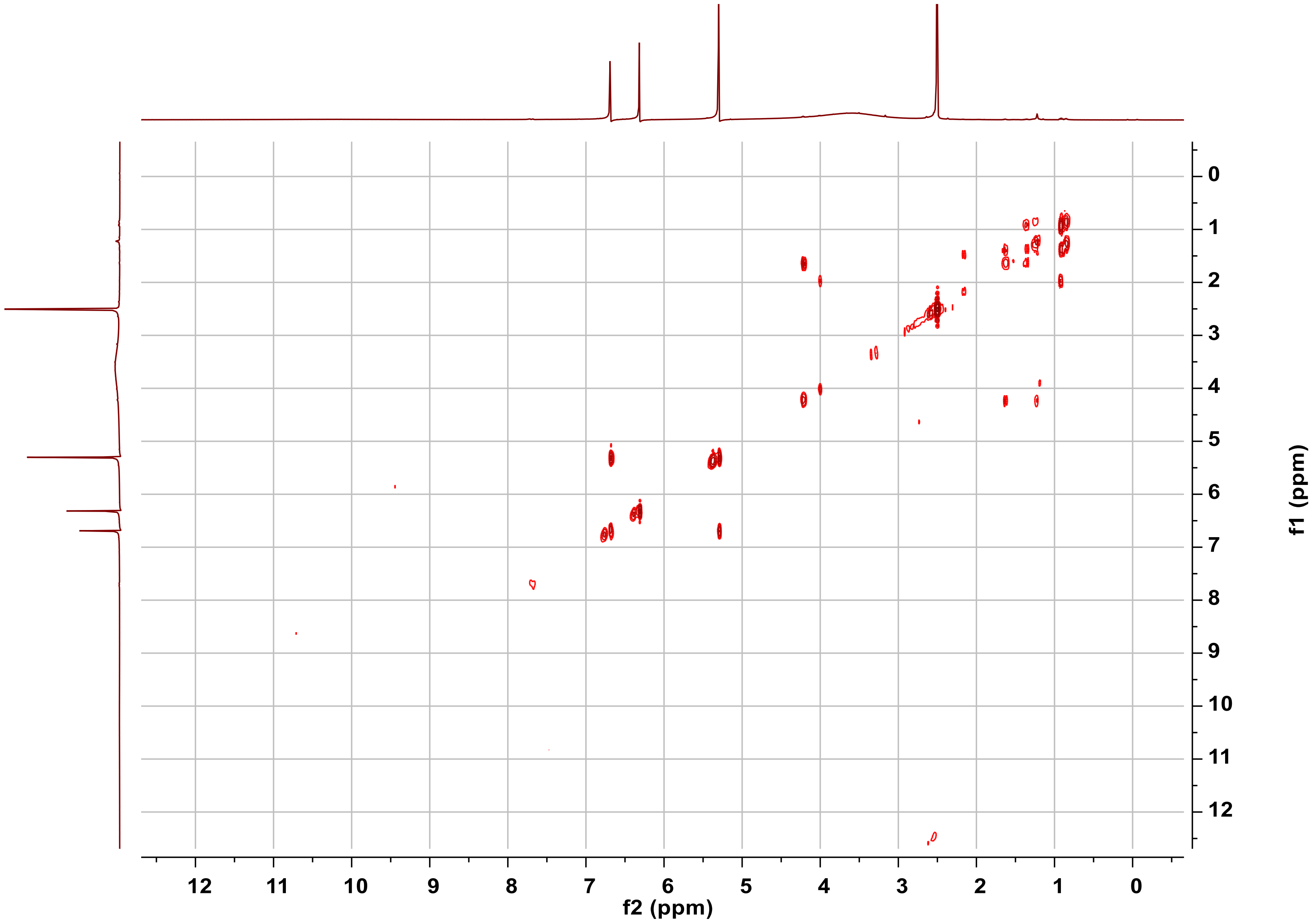

Figure S14 ^{1}H NMR spectrum of **6** in DMSO-d_6 (500 MHz)

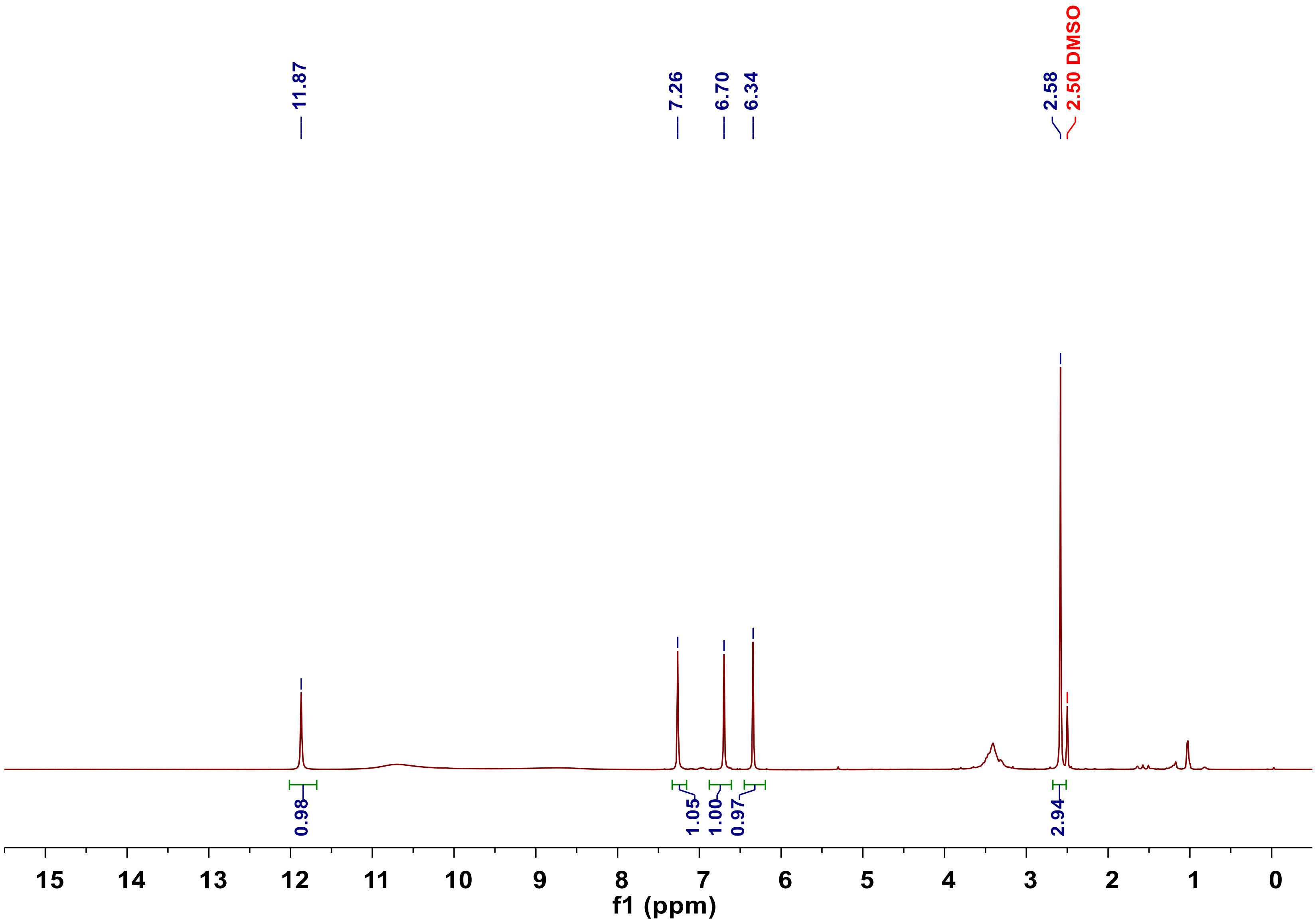

Figure S15 ^{13}C NMR spectrum of **6** in DMSO-d_6 (125MHz)

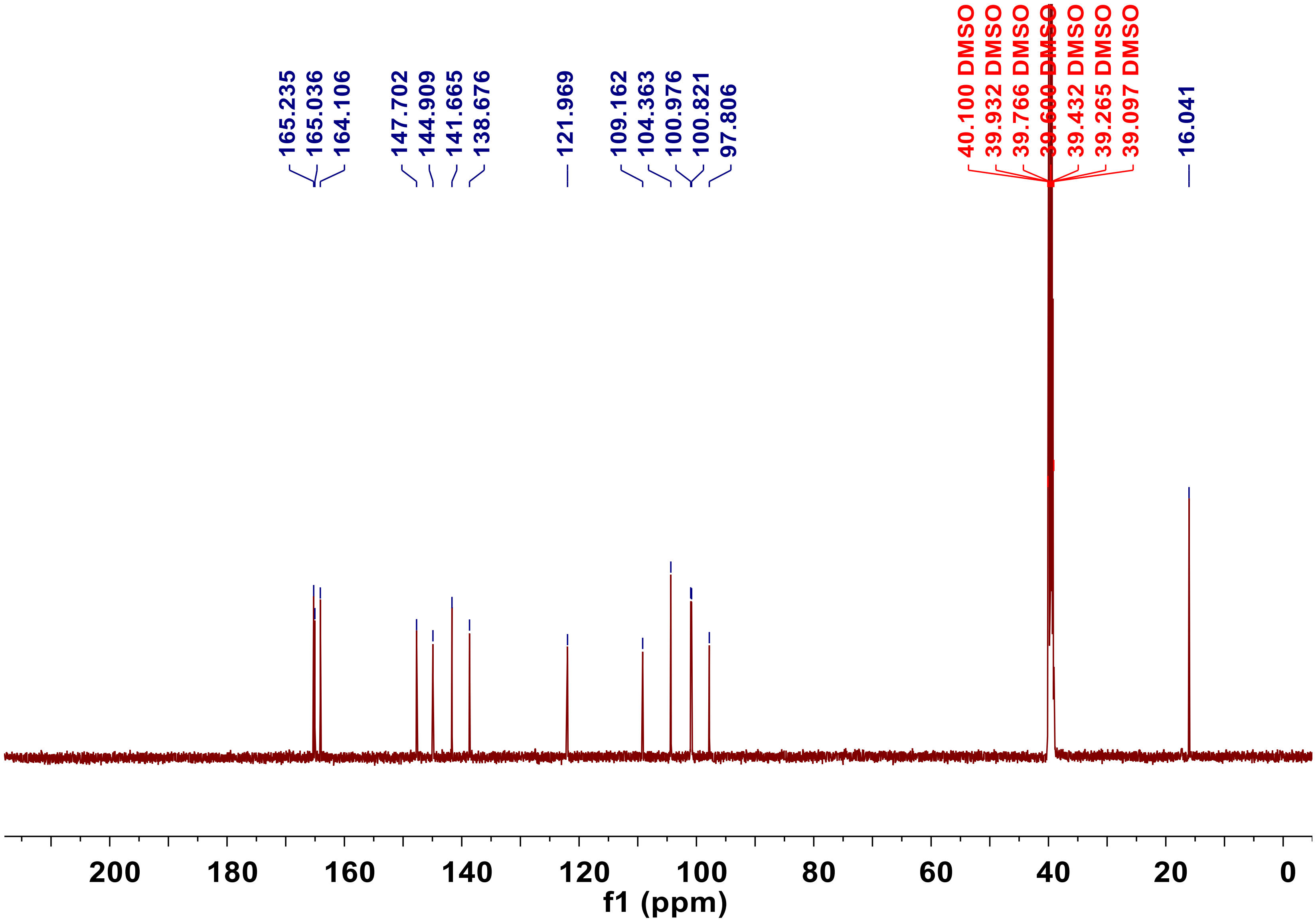

Figure S16 DEPT-90 spectrum of **6**

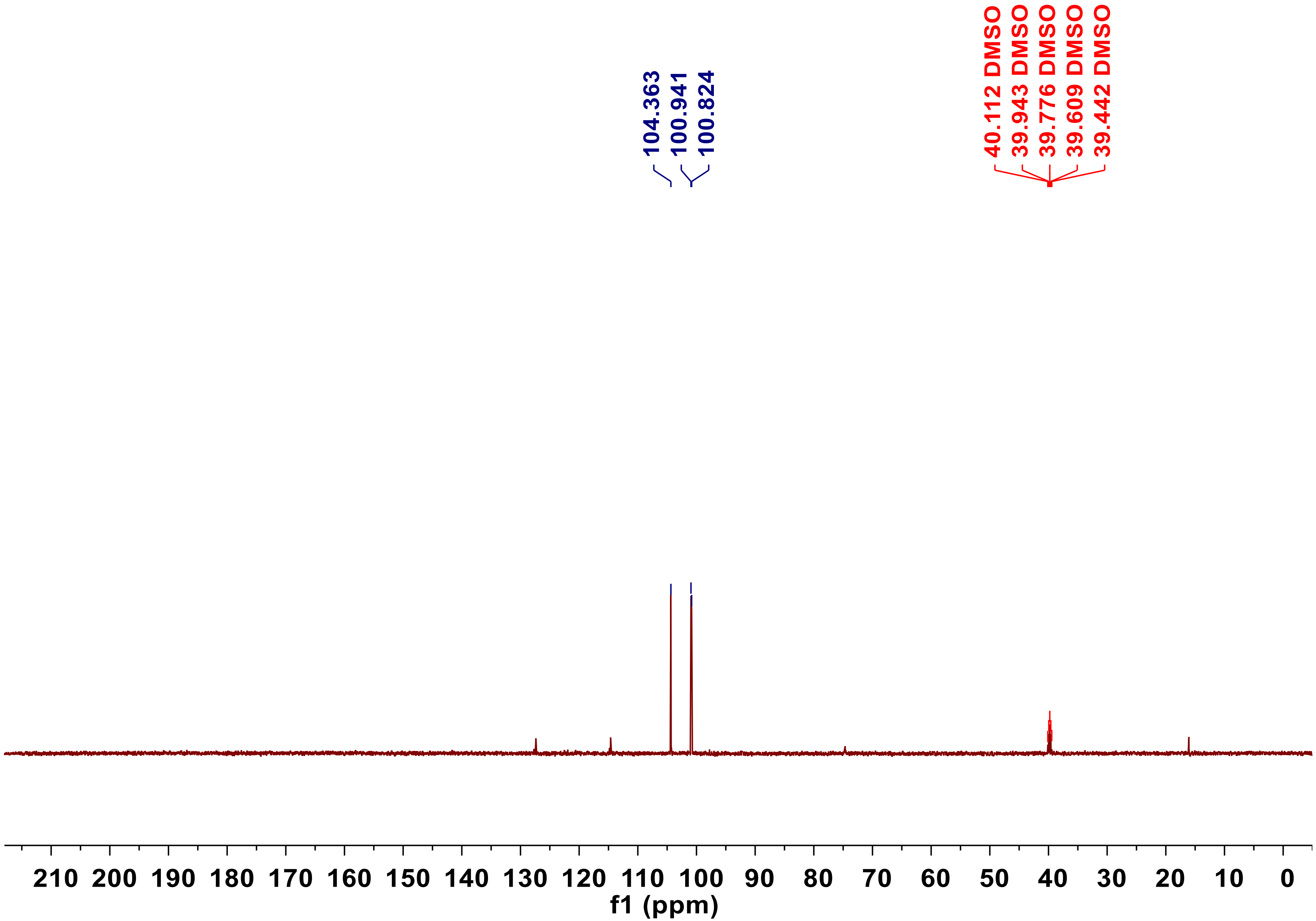

Figure S17 DEPT-135 spectrum of **6**

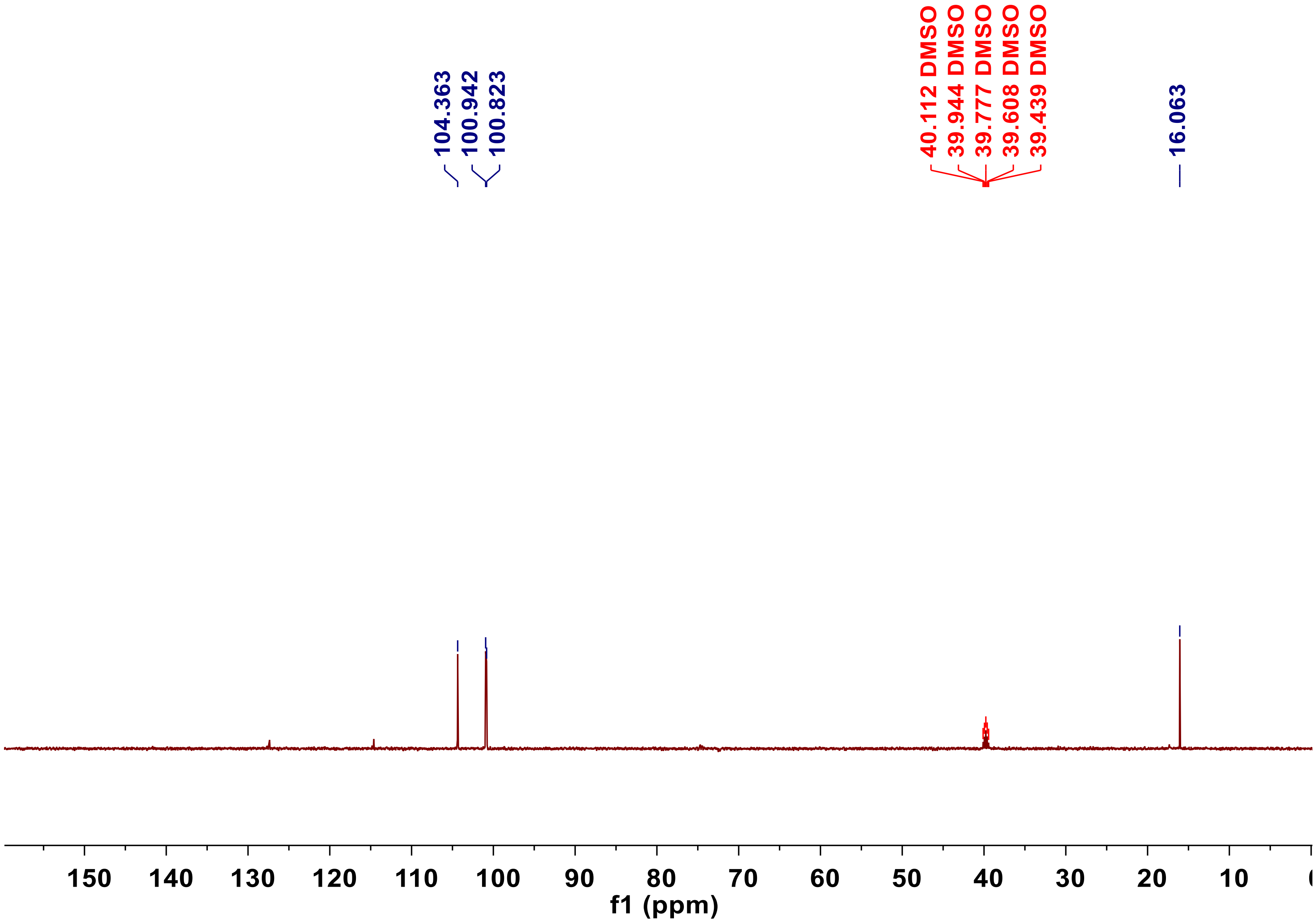

Figure S18 HSQC spectrum of **6**

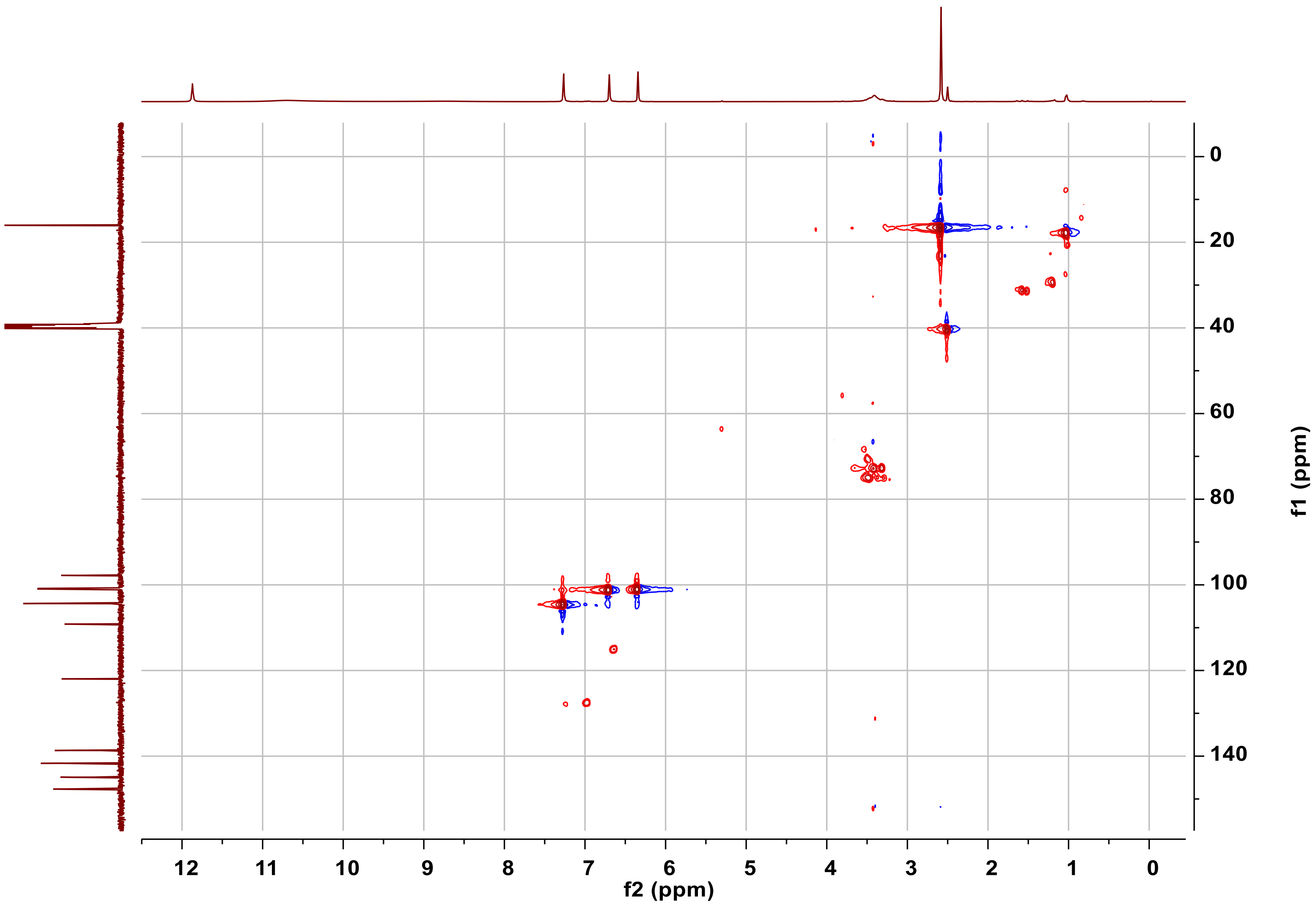

Figure S19 HMBC spectrum of **6**

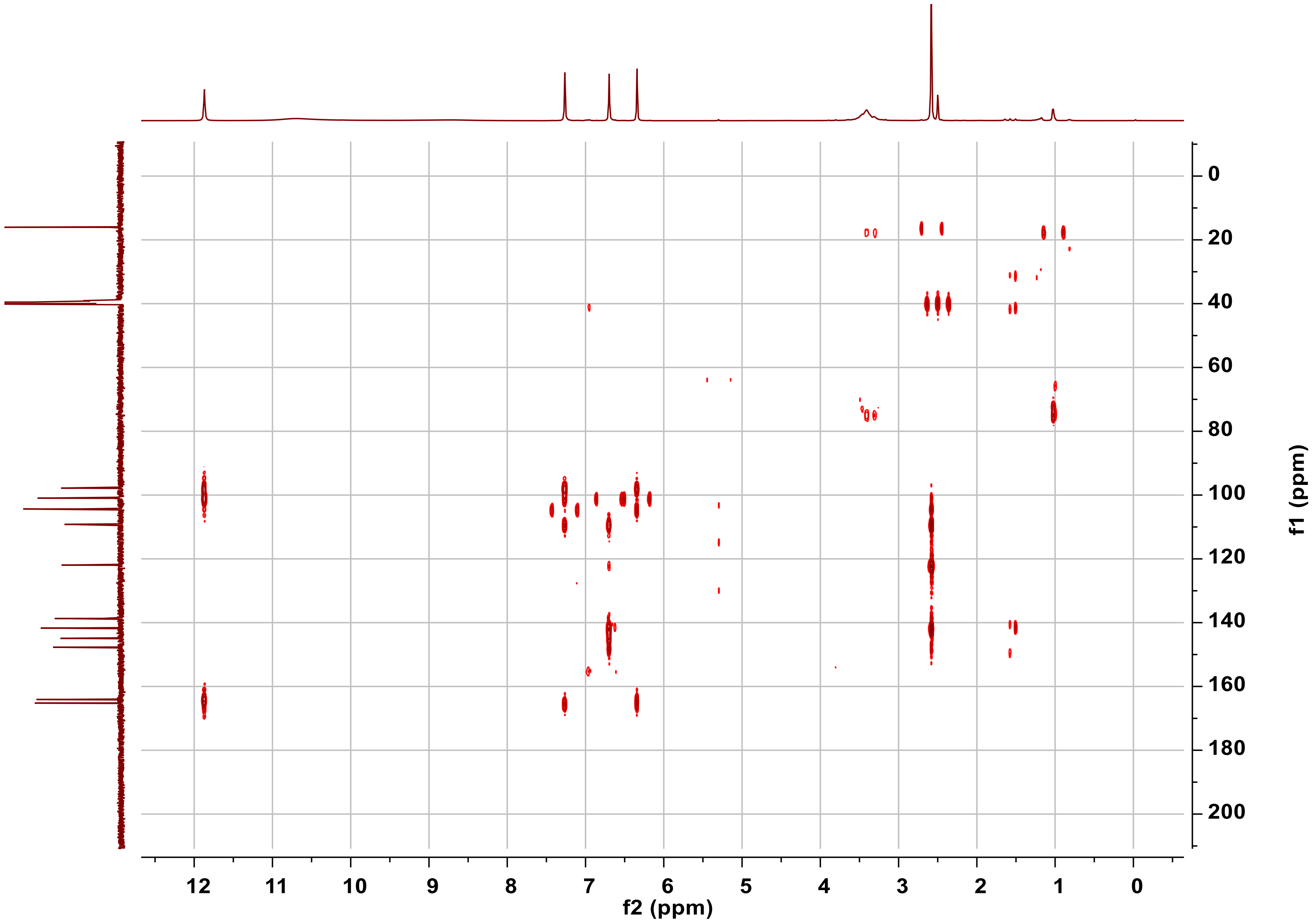

Figure S20 ^{1}H-^{1}H COSY spectrum of **6**

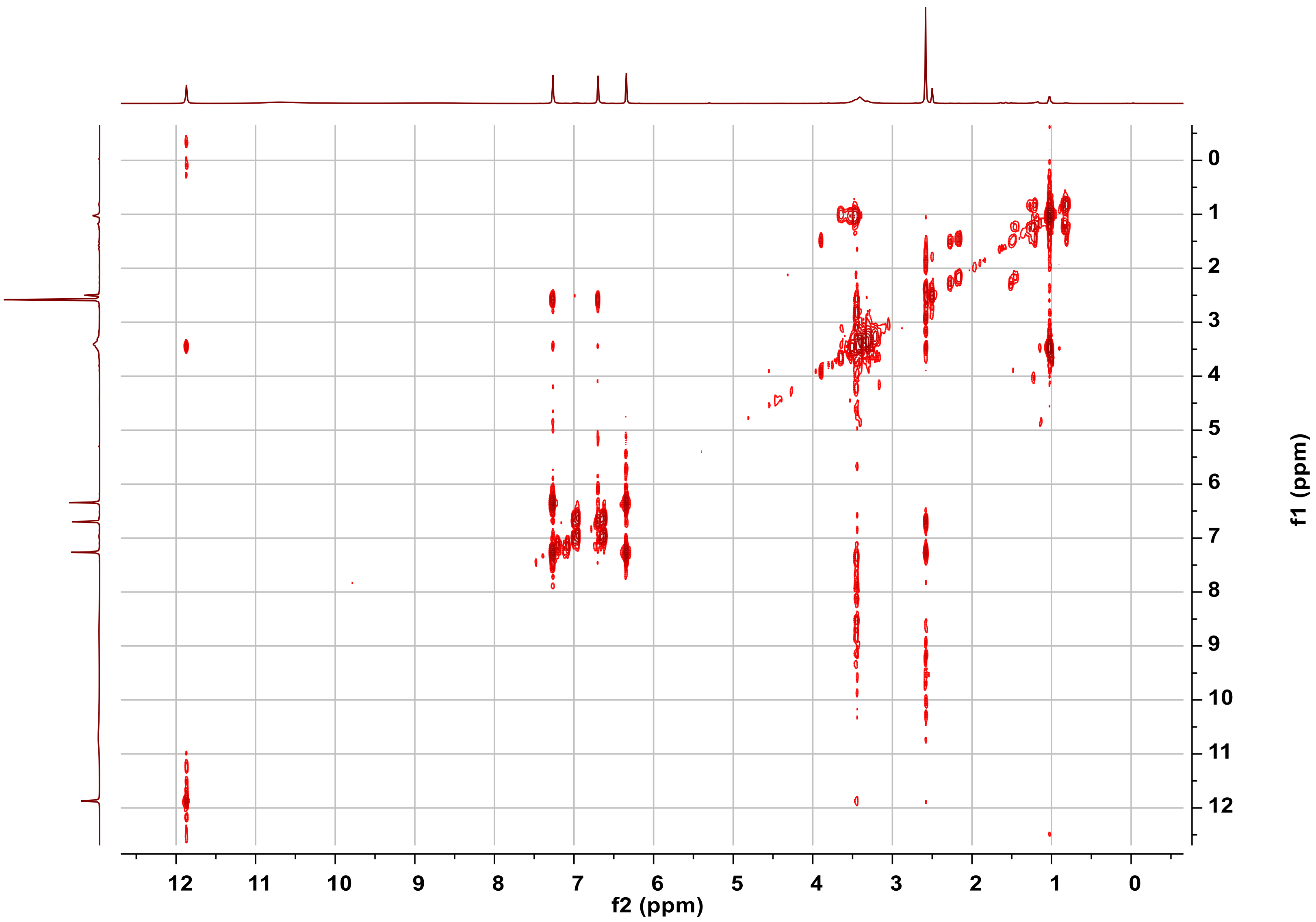

Figure S21 ^{1}H NMR spectrum of **7** in DMSO (400 MHz)

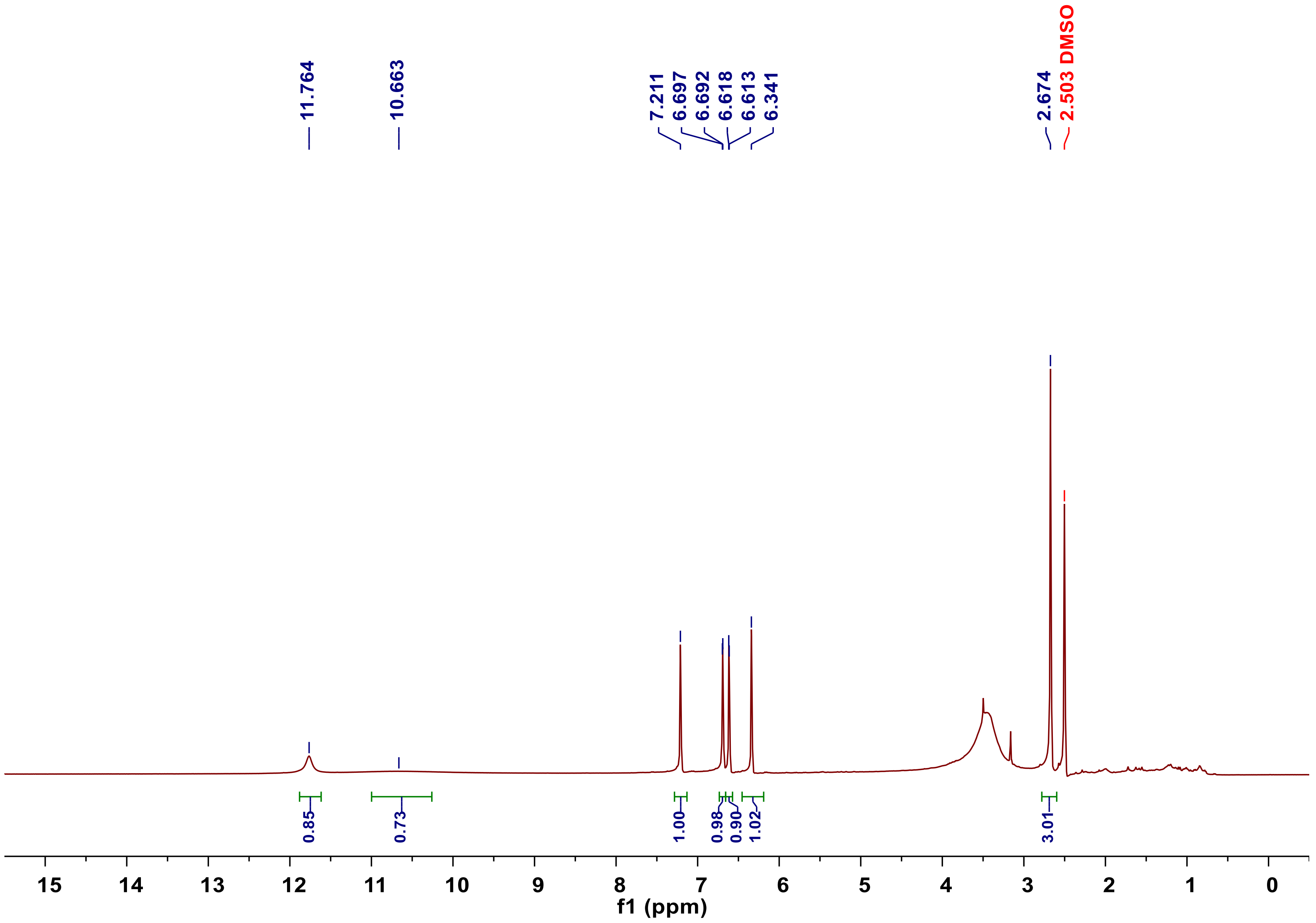

Figure S22 ^{13}C NMR spectrum of **7** in DMSO (100 MHz)

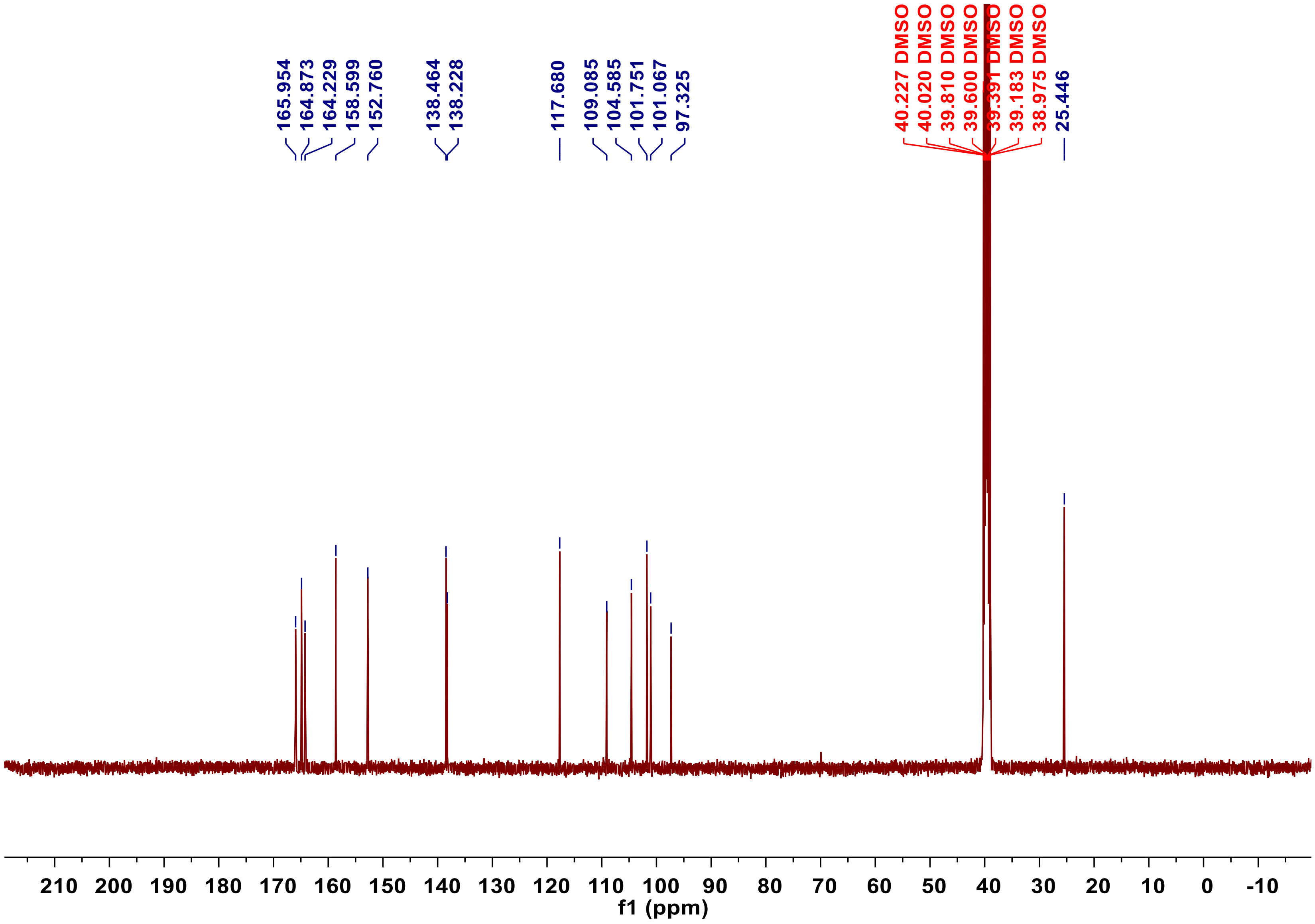

Figure S23 DEPT-90 spectrum of **7**

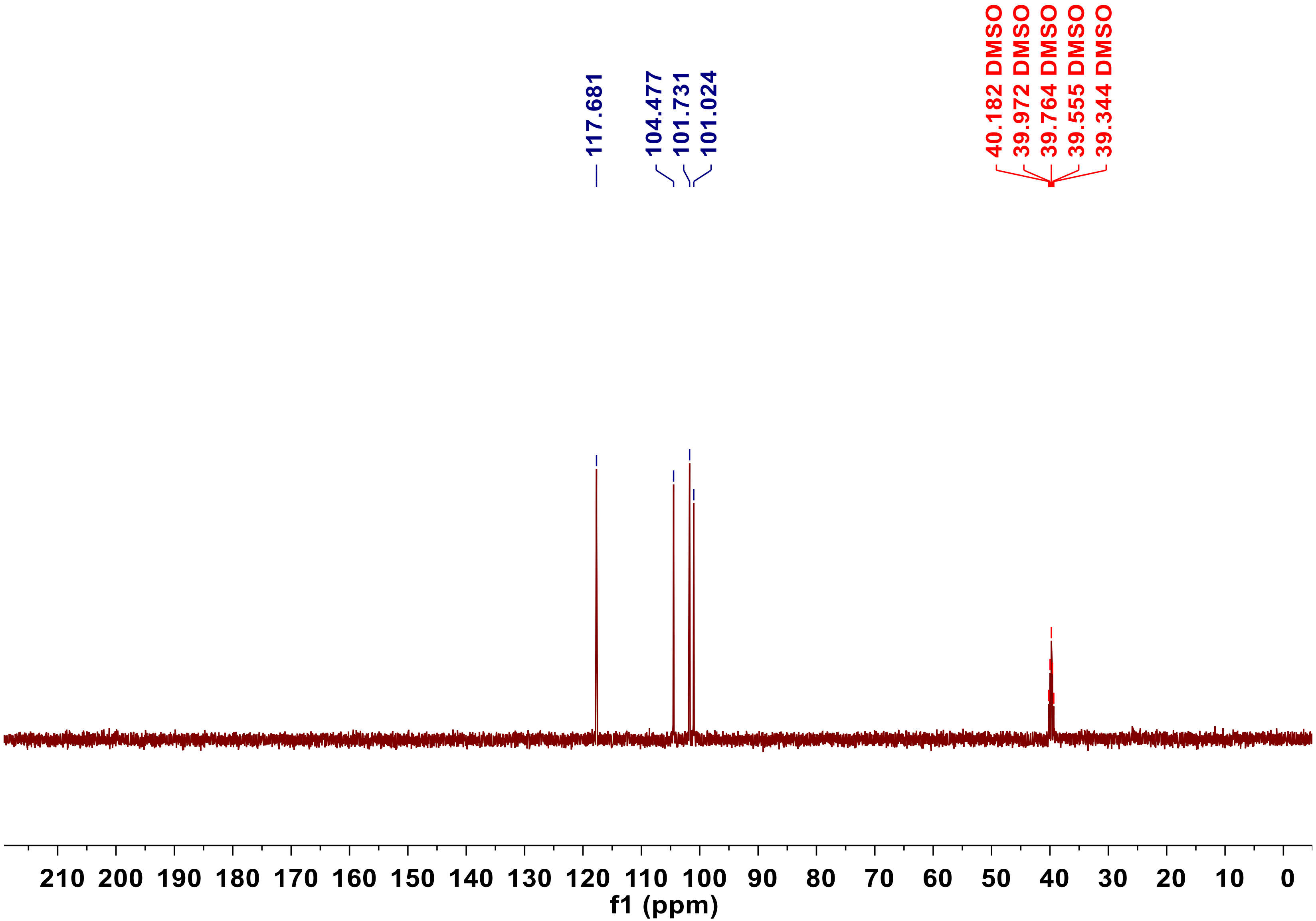

Figure S24 DEPT-135 spectrum of **7**

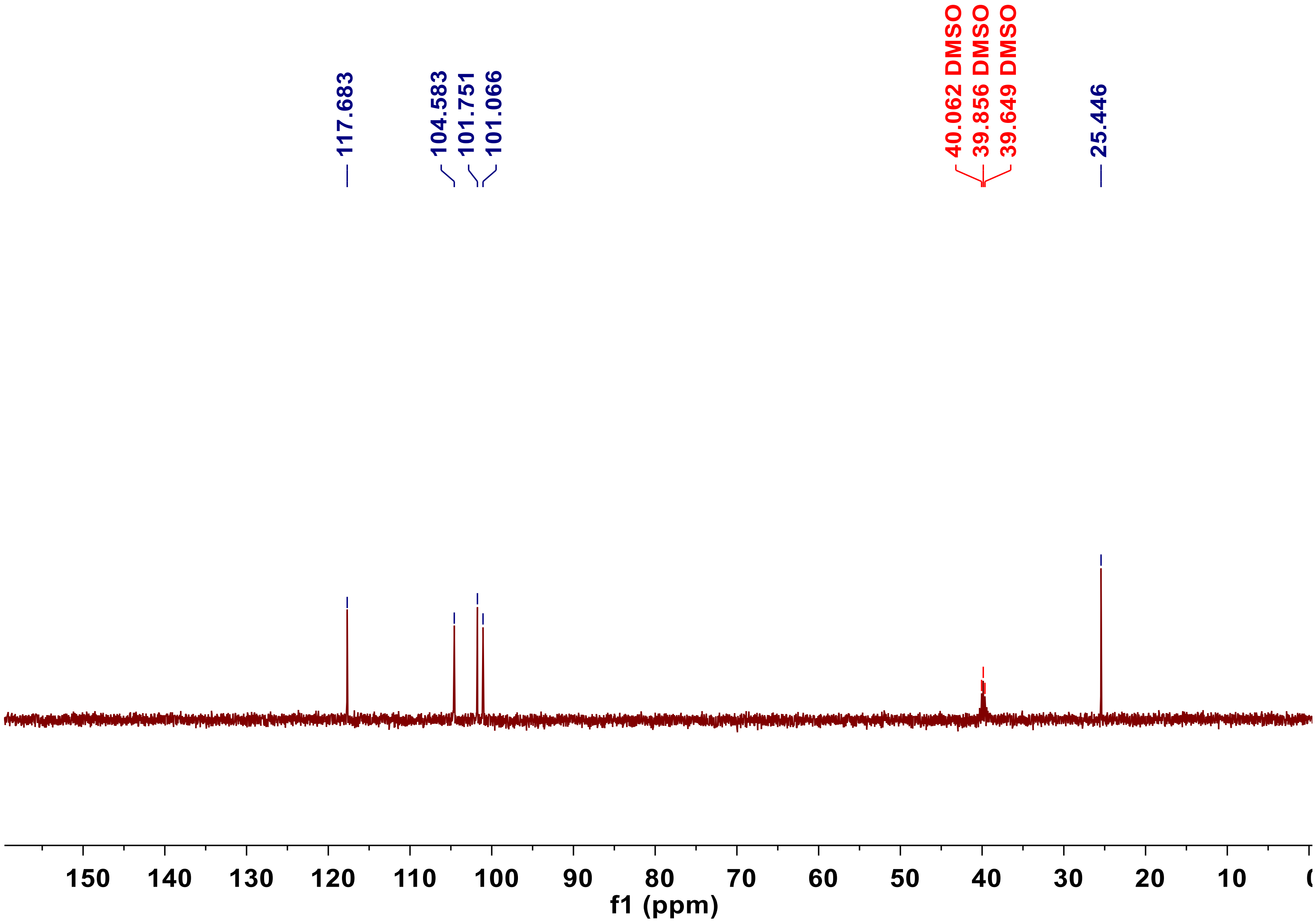

Figure S25 HSQC spectrum of **7**

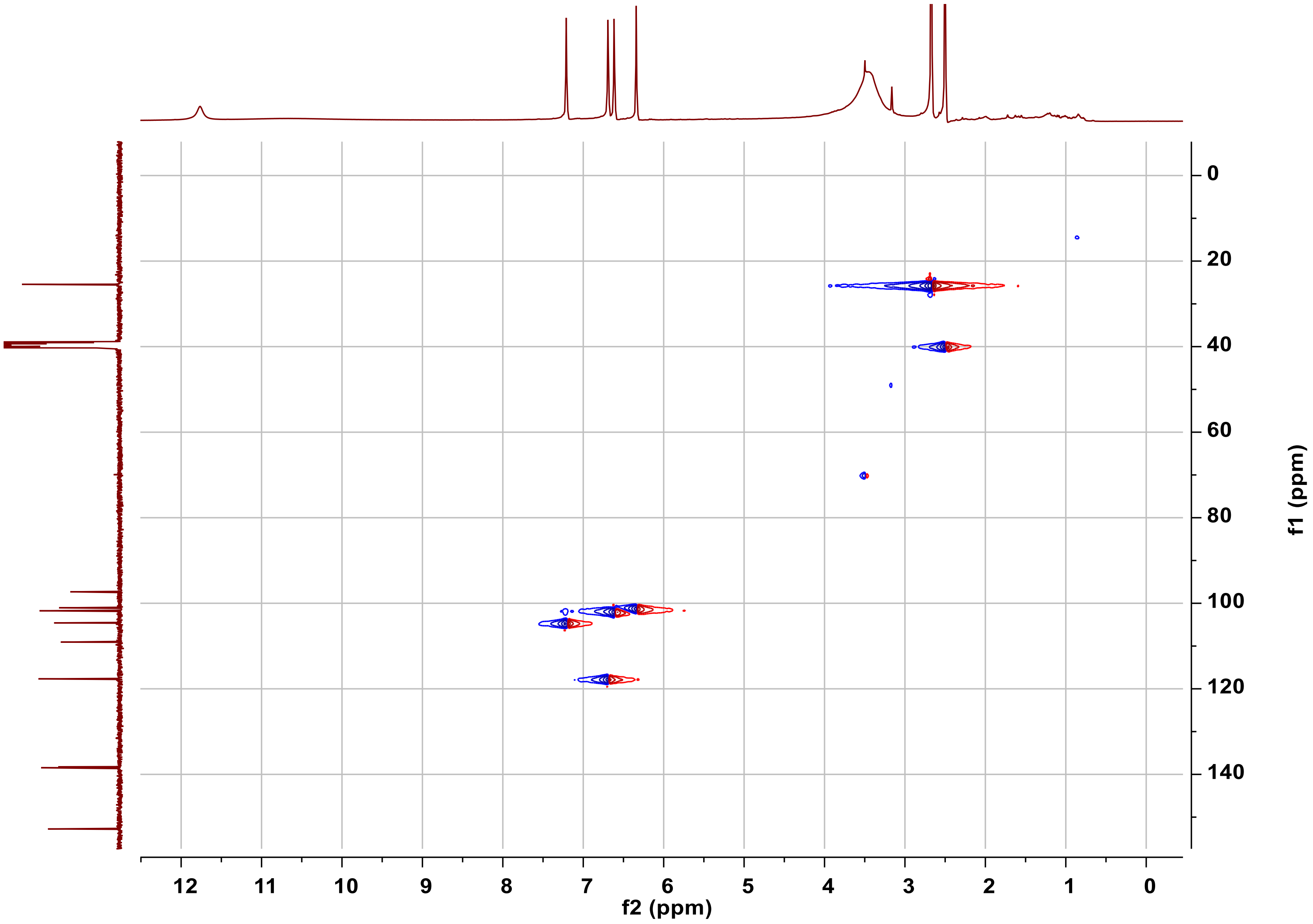

Figure S26 HMBC spectrum of **7**

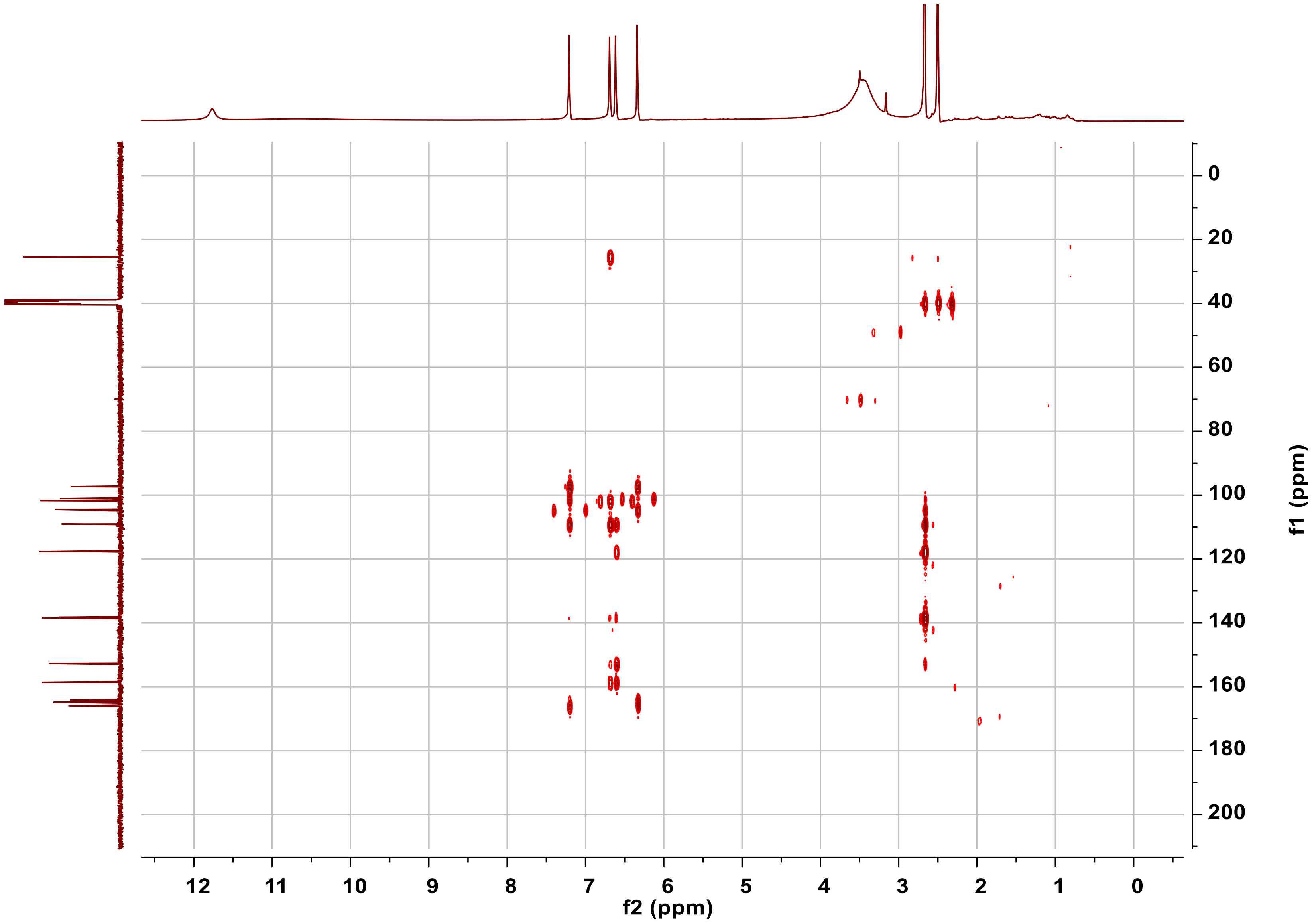

Figure S27 ^{1}H-^{1}H COSY spectrum of **7**

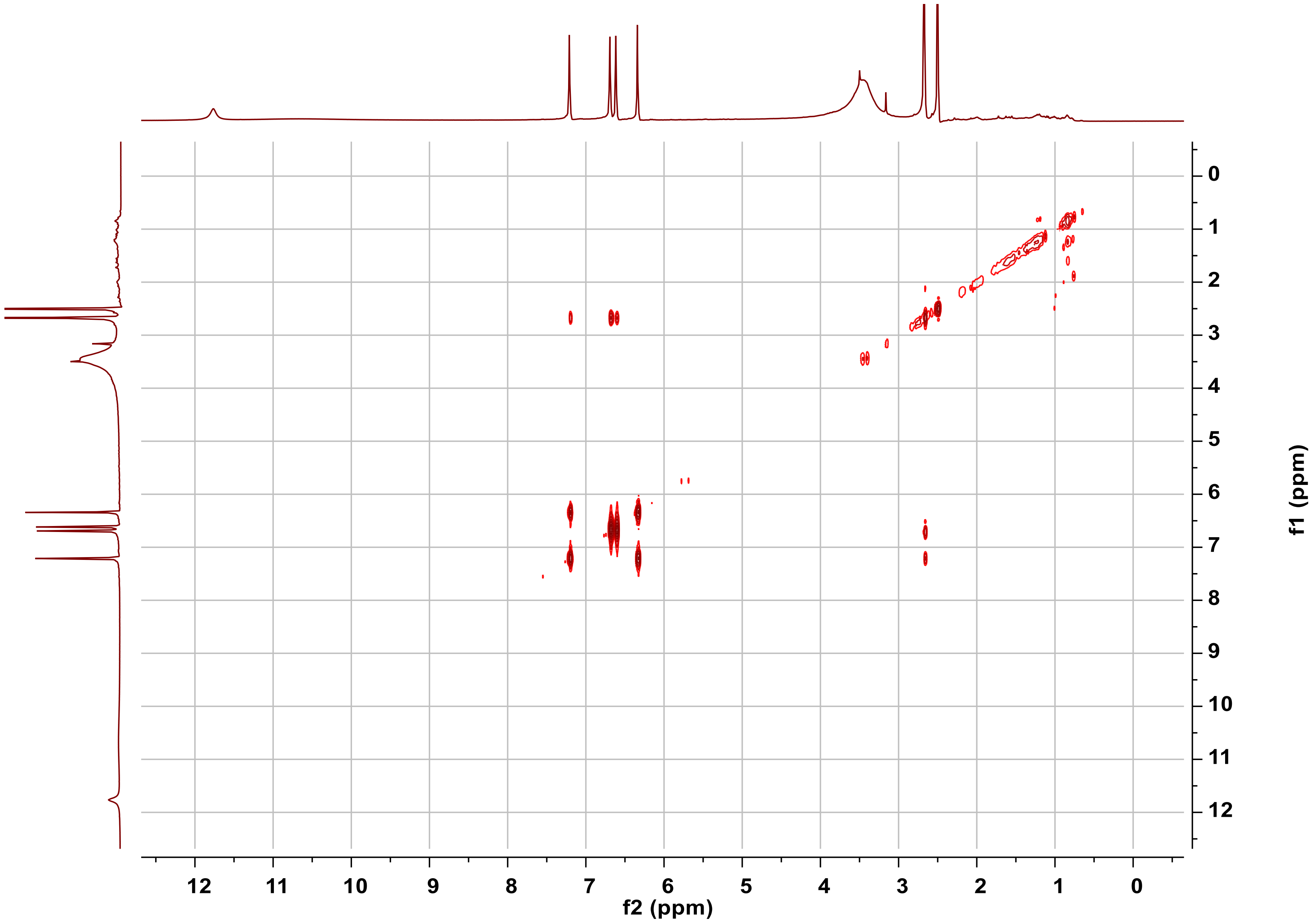

Figure S28 ^{1}H NMR spectrum of **8** in DMSO-d_6 (500 MHz)

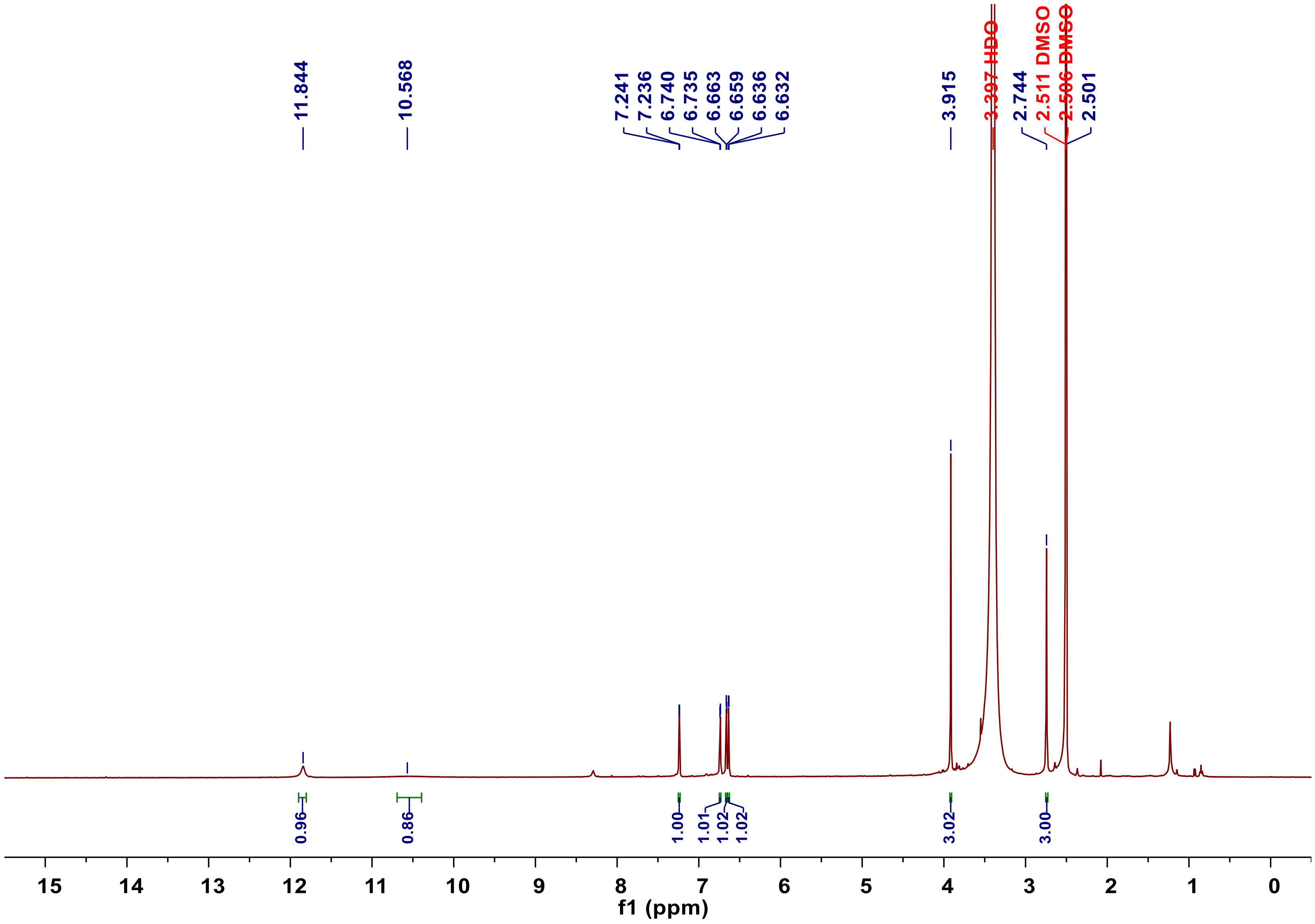

Figure S29 ^{13}C NMR spectrum of **8** in DMSO-d_6 (125 MHz)

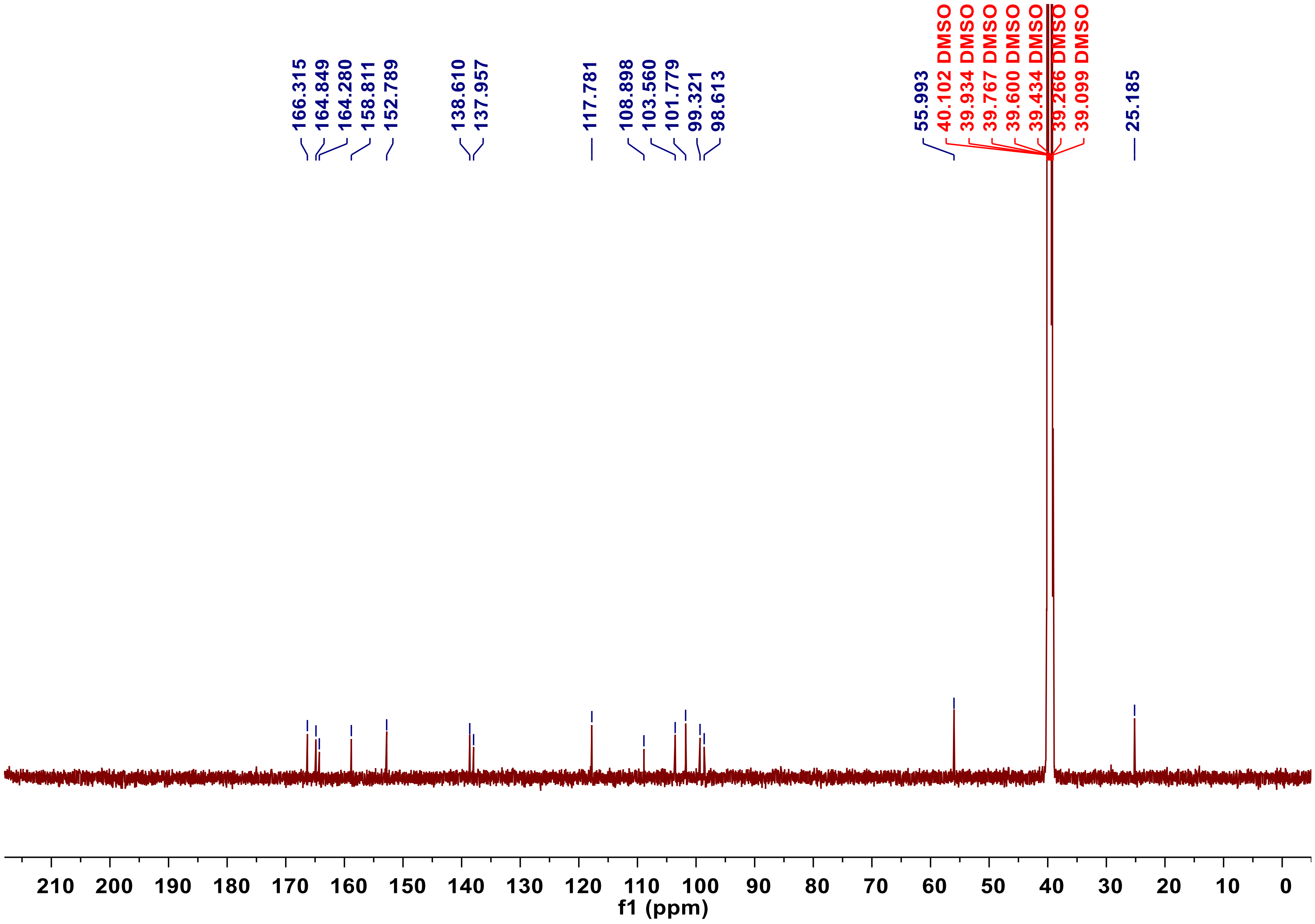

Figure S30 DEPT-90 spectrum of **8**

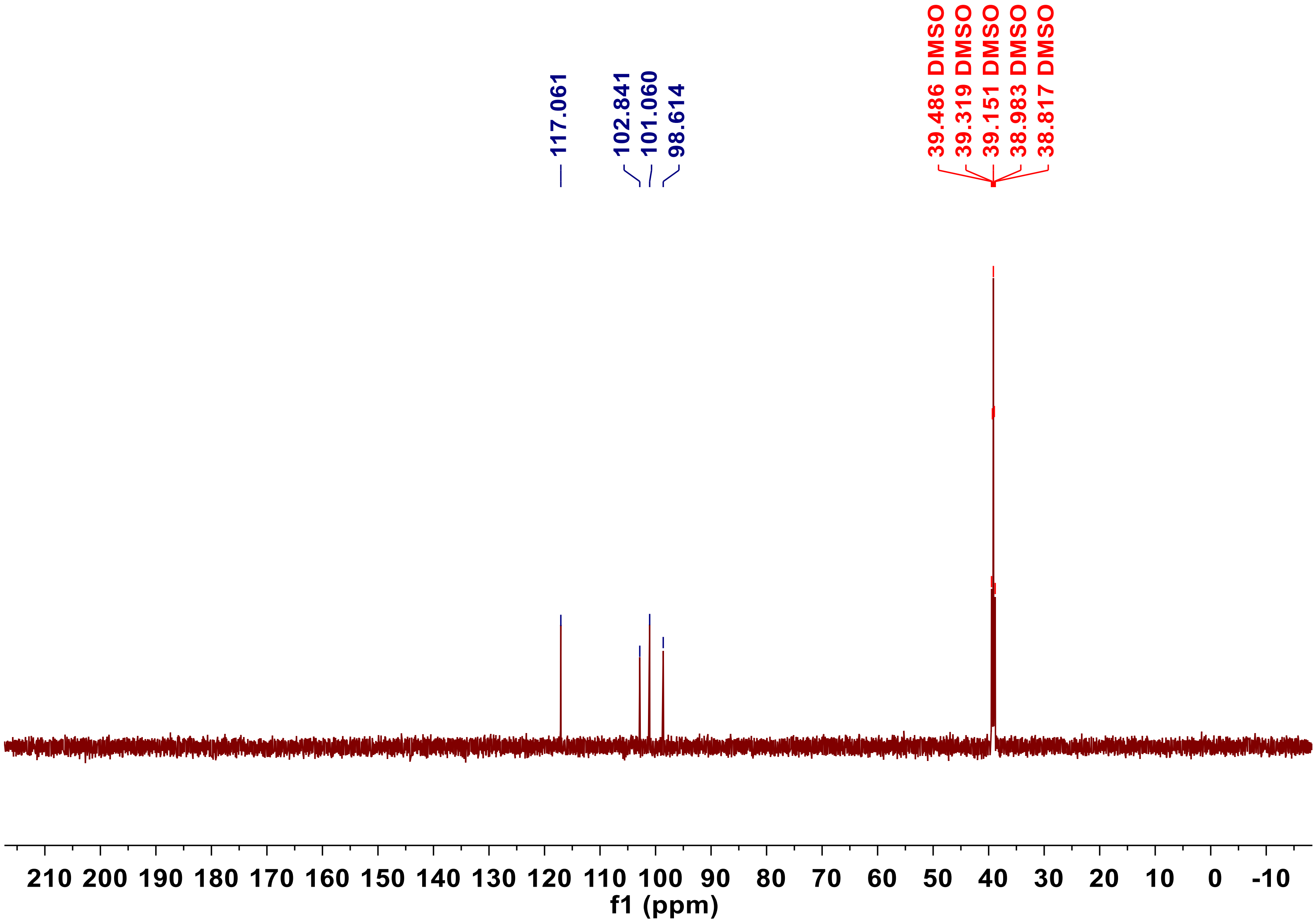

Figure S31 DEPT-135 spectrum of **8**

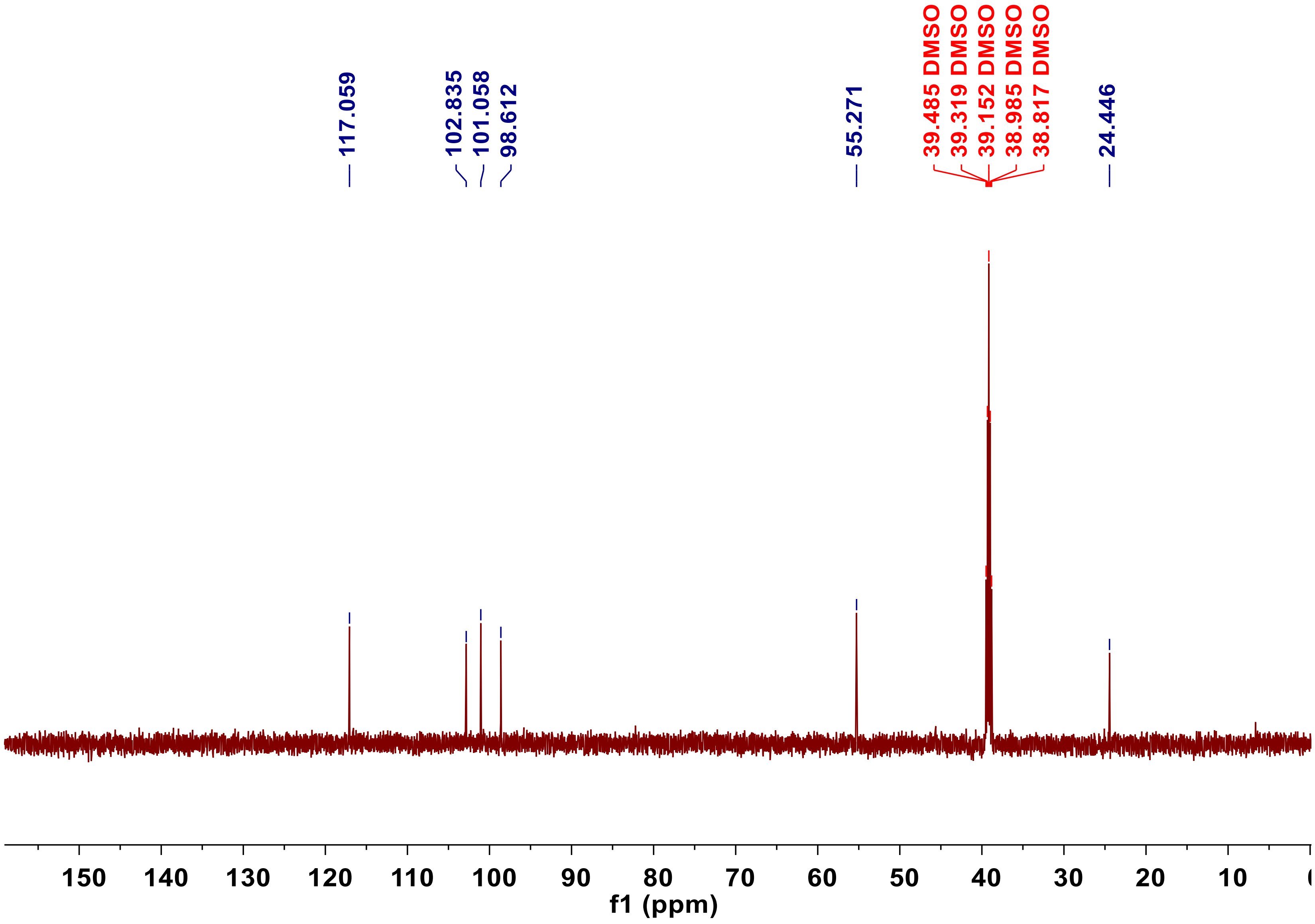

Figure S32 HSQC spectrum of **8**

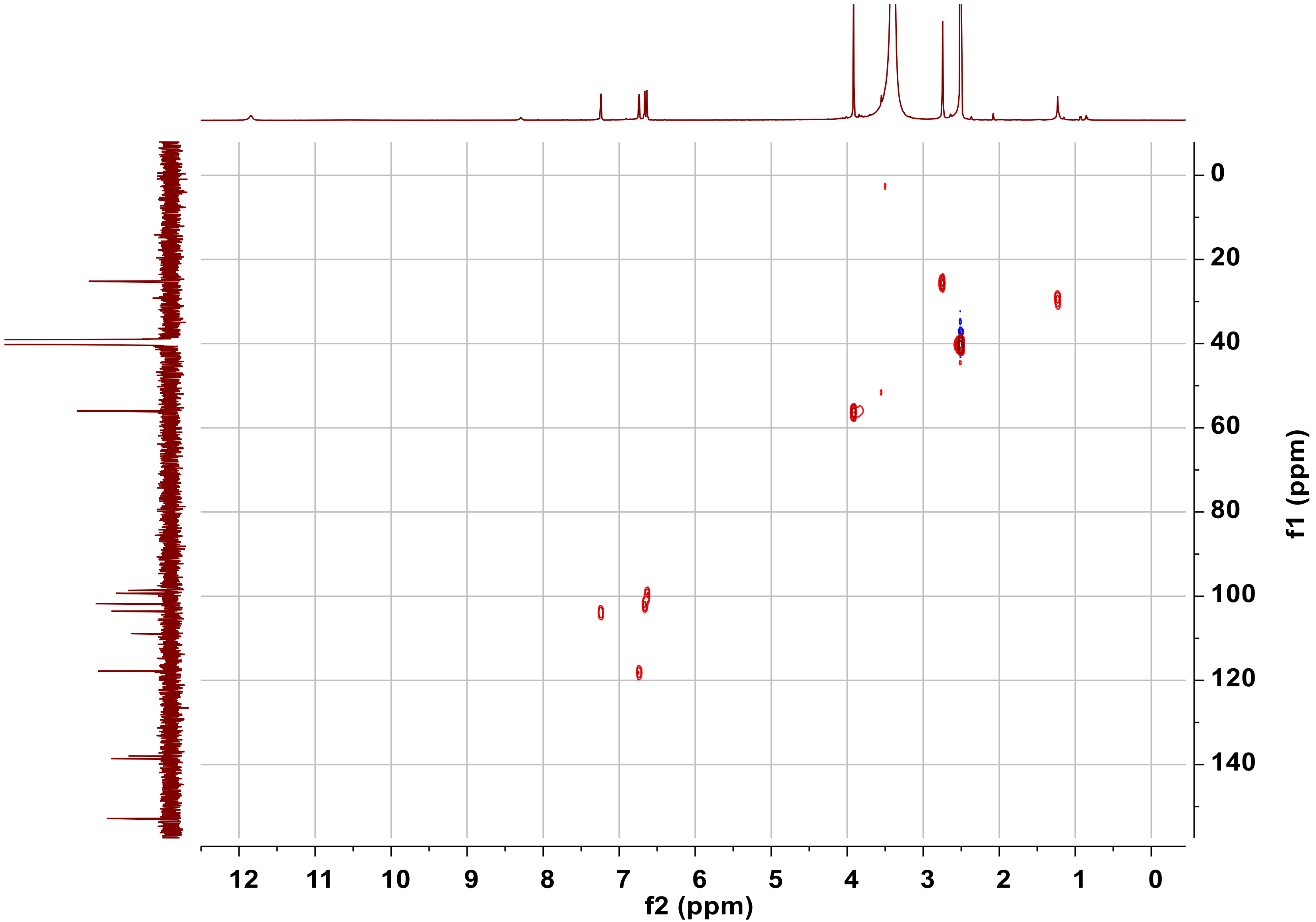

Figure S33 HMBC spectrum of **8**

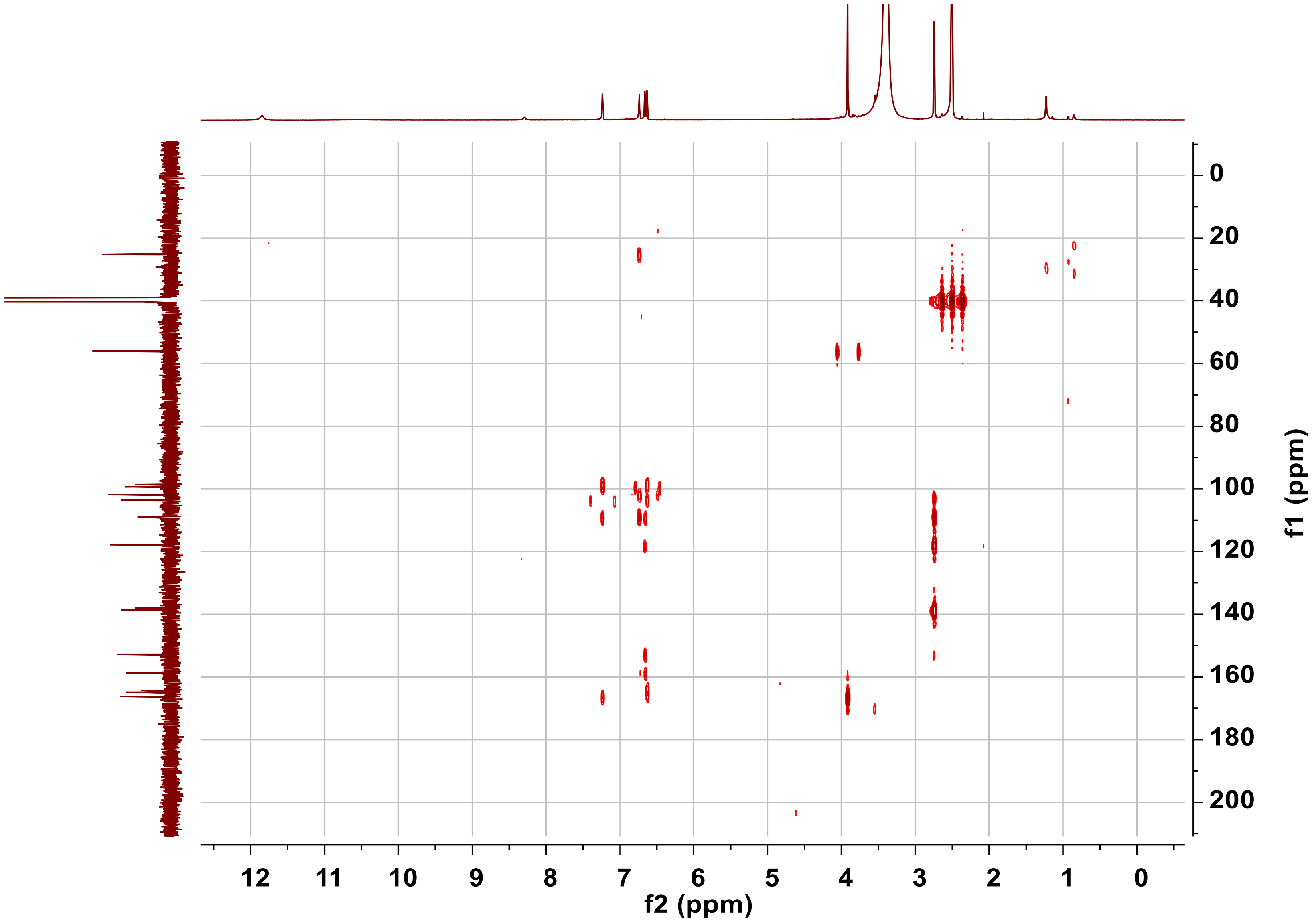

Figure S34 ^{1}H-^{1}H COSY spectrum of **8**

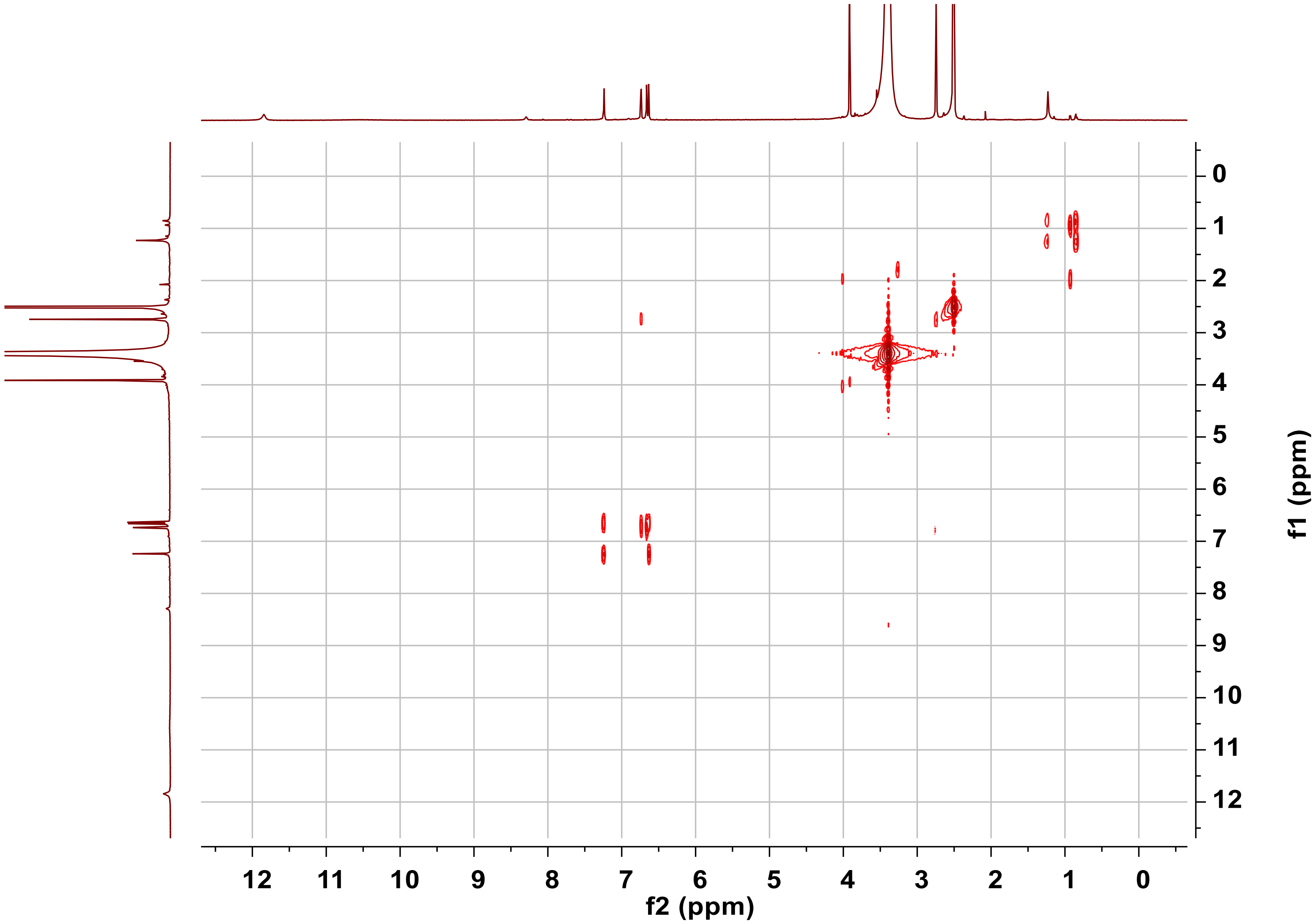

Figure S35 ^{1}H NMR spectrum of **9** in CD_3OD (400 MHz)

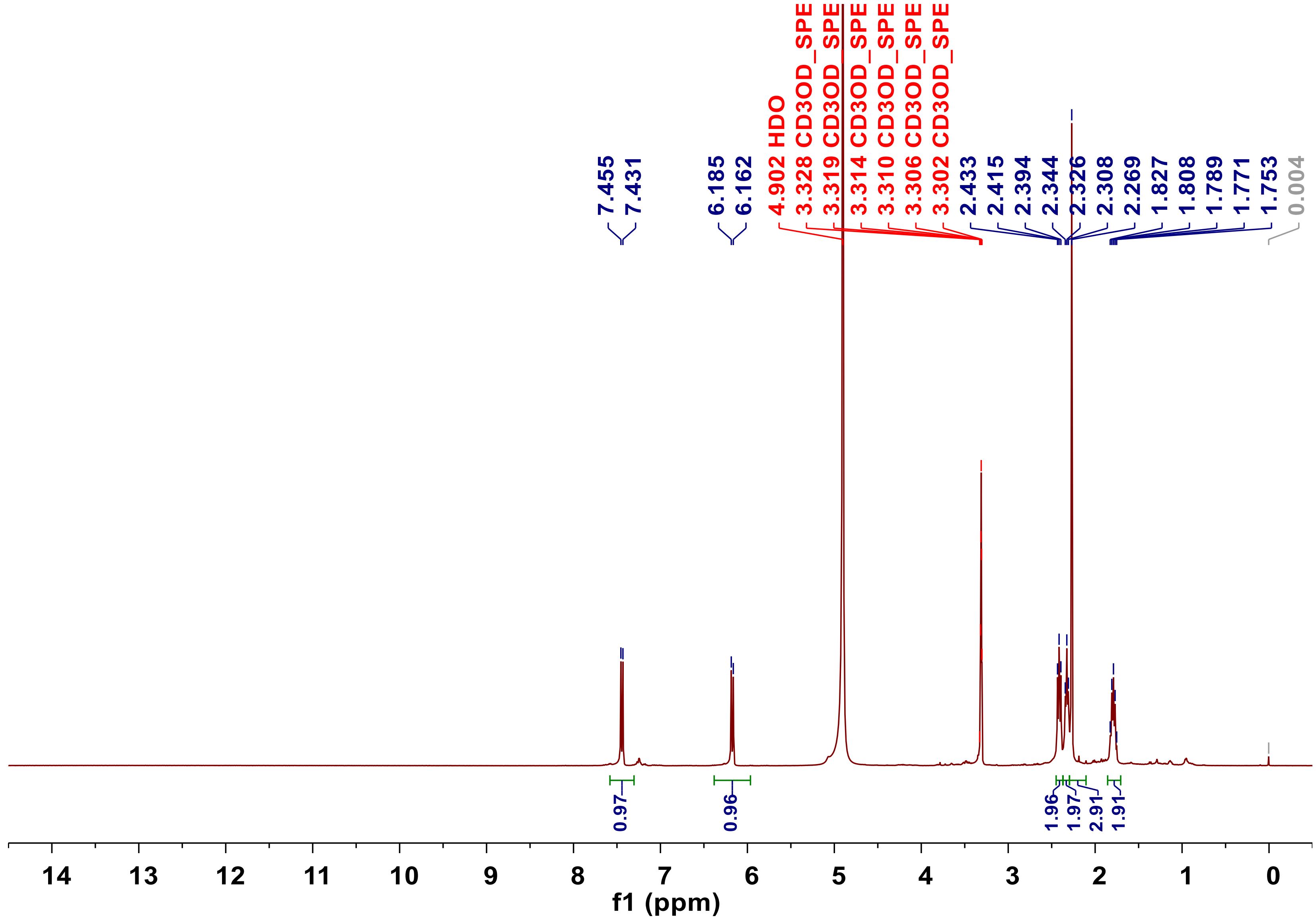

Figure S36 ^{13}C NMR spectrum of **9** in CD_3OD (100 MHz)

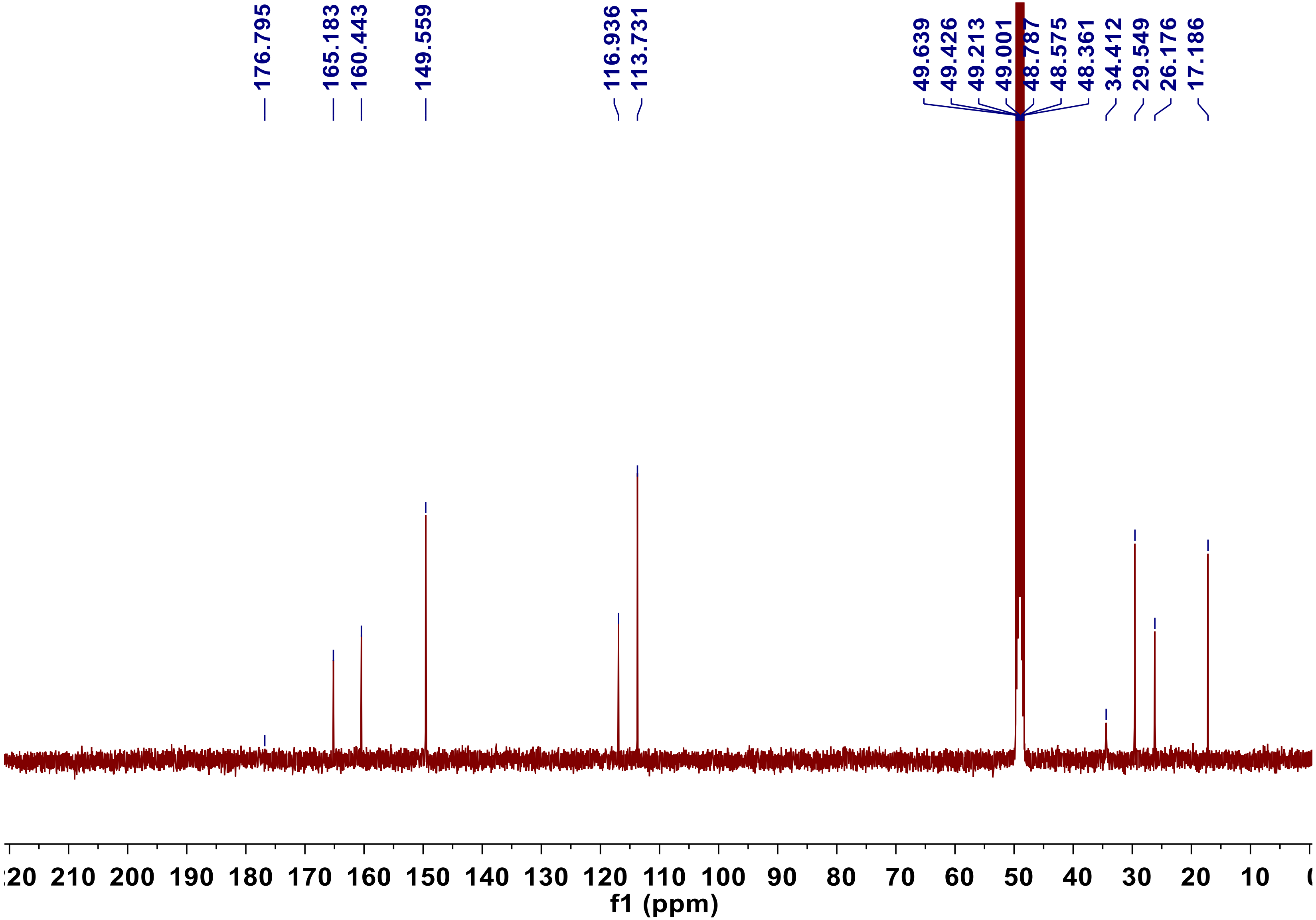

Figure S37 DEPT-90 spectrum of **9**

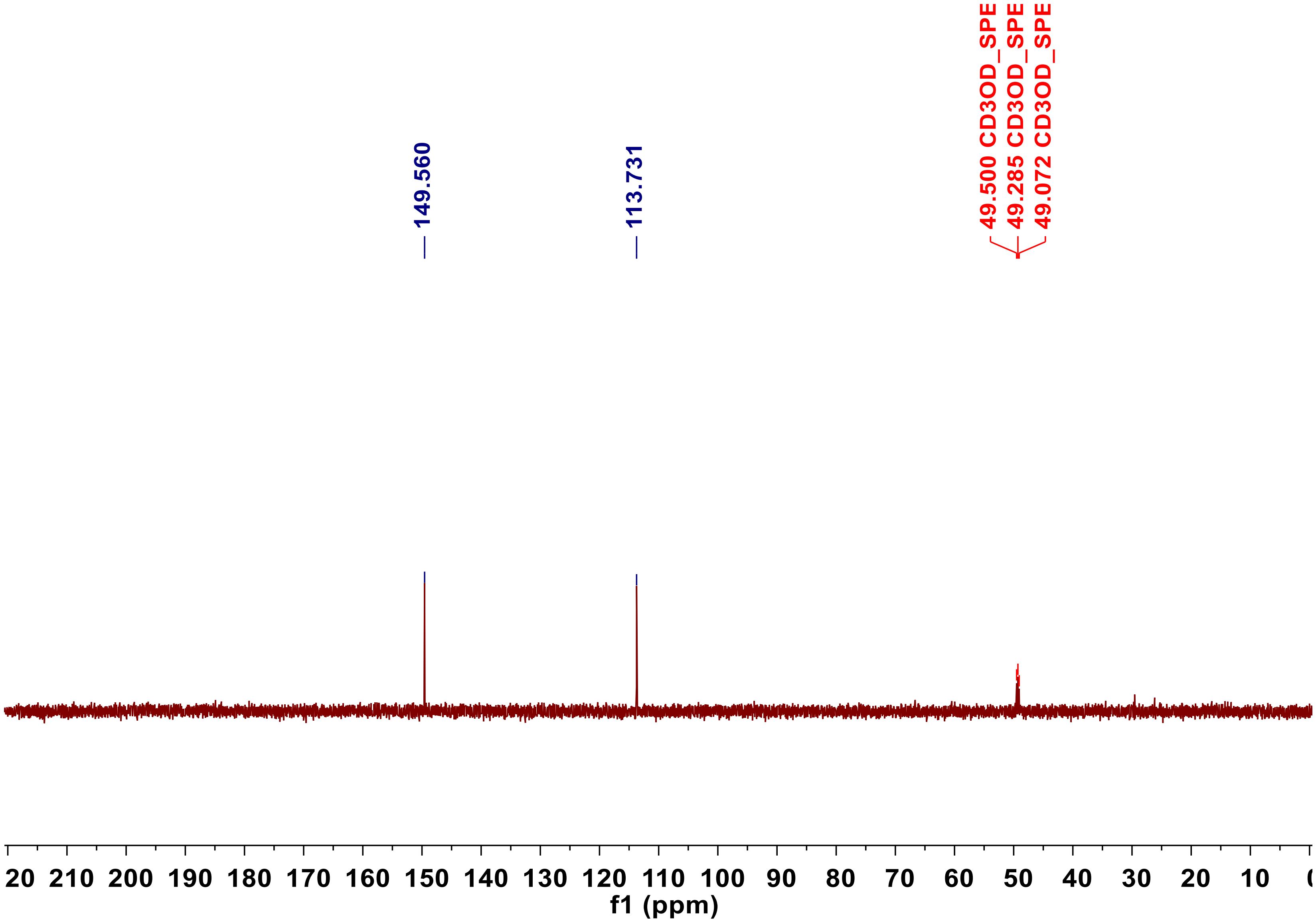

Figure S38 DEPT-135 spectrum of **9**

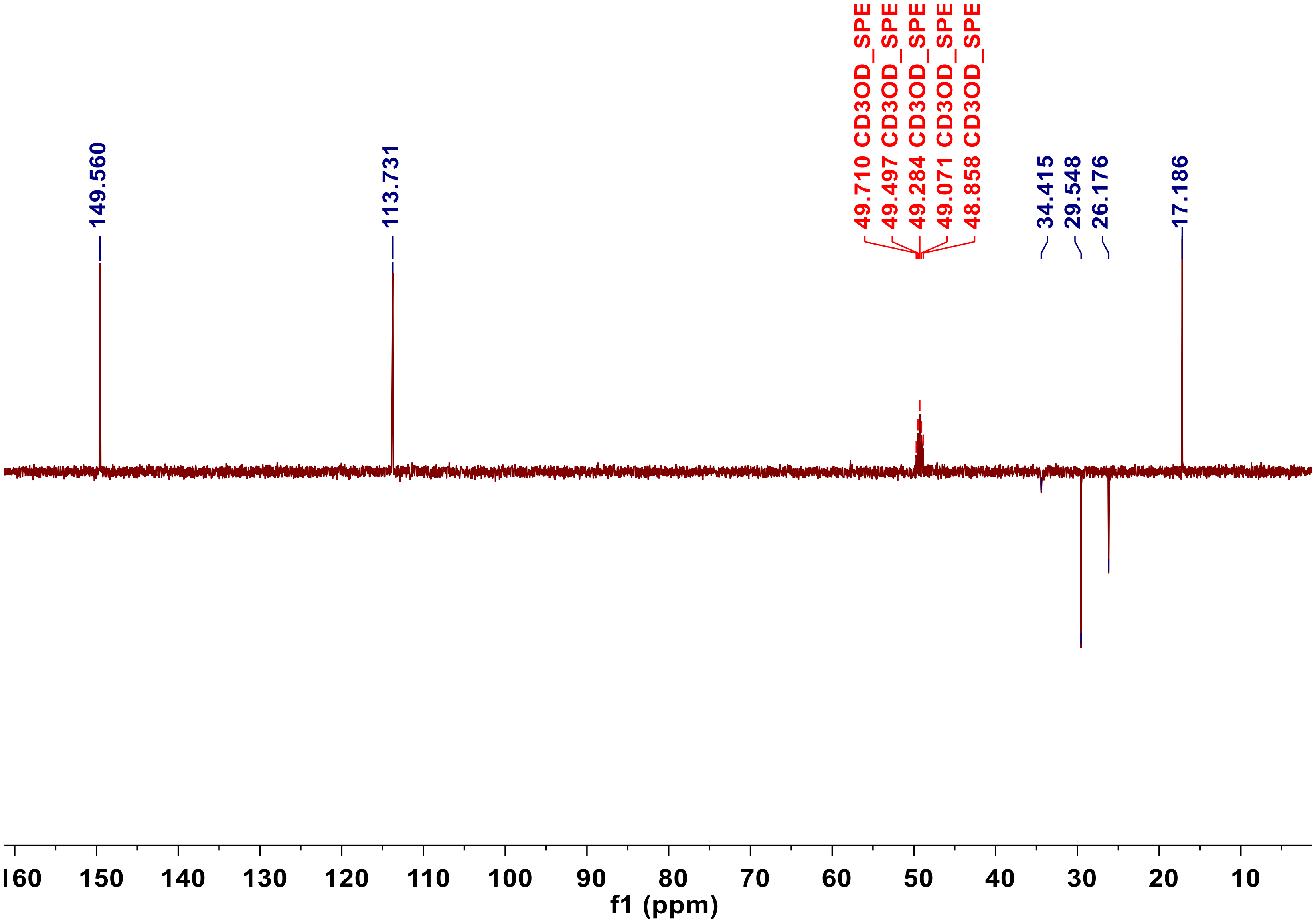

Figure S39 HSQC spectrum of **9**

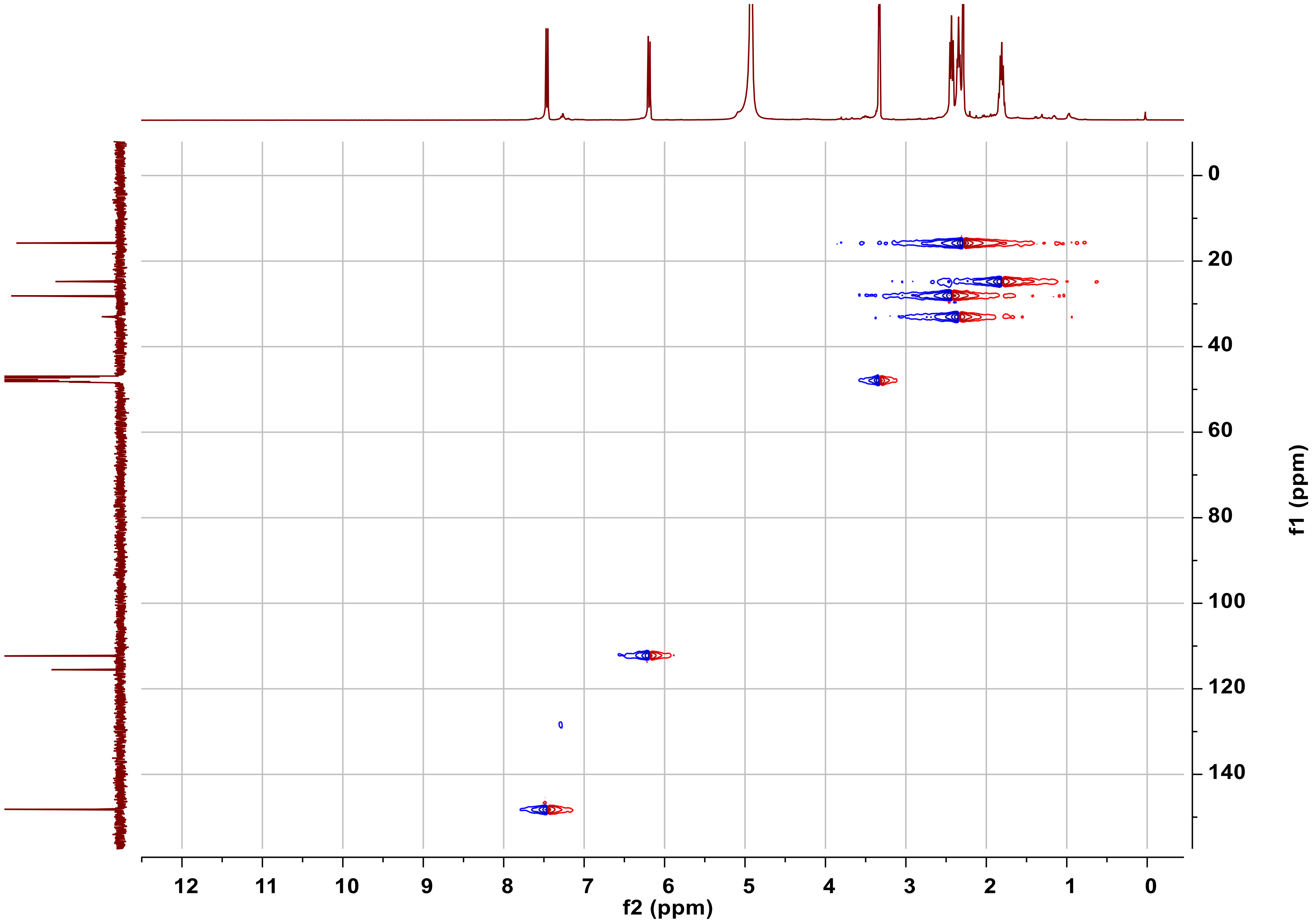

Figure S40 HMBC spectrum of **9**

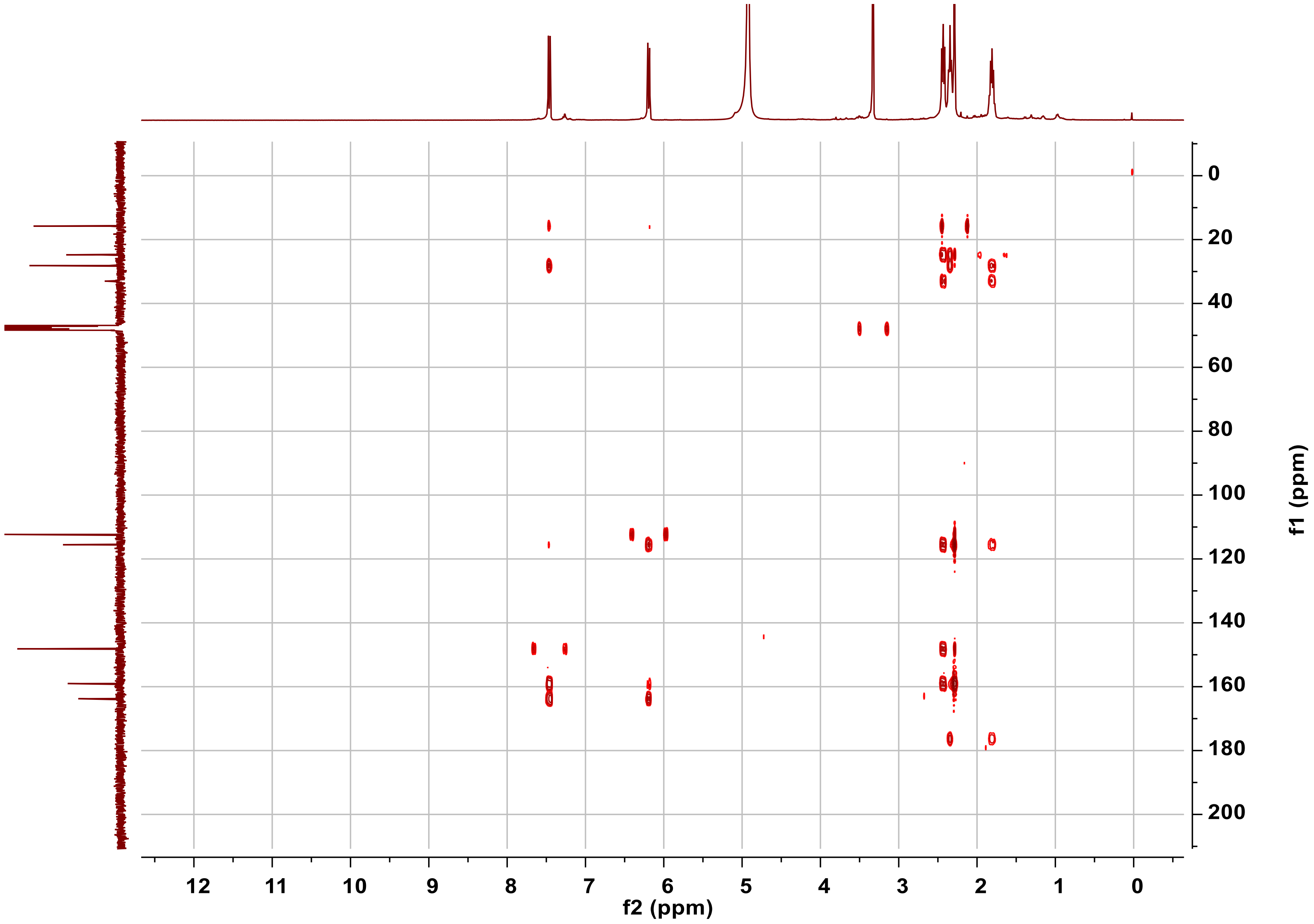

Figure S41 ^{1}H-^{1}H COSY spectrum of **9**

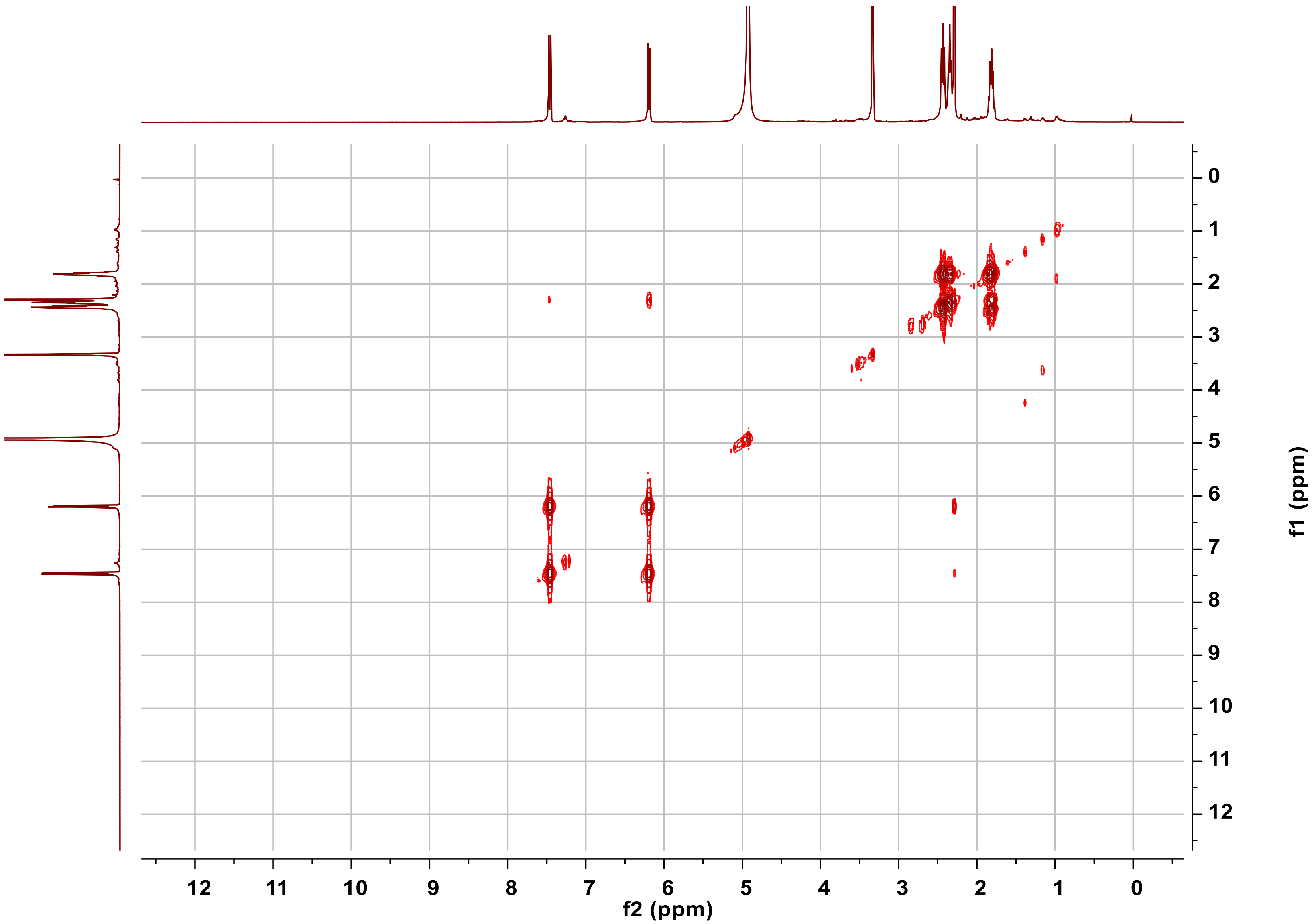

Figure S42 ^{1}H NMR spectrum of **9** in DMSO-d_6 (400 MHz)

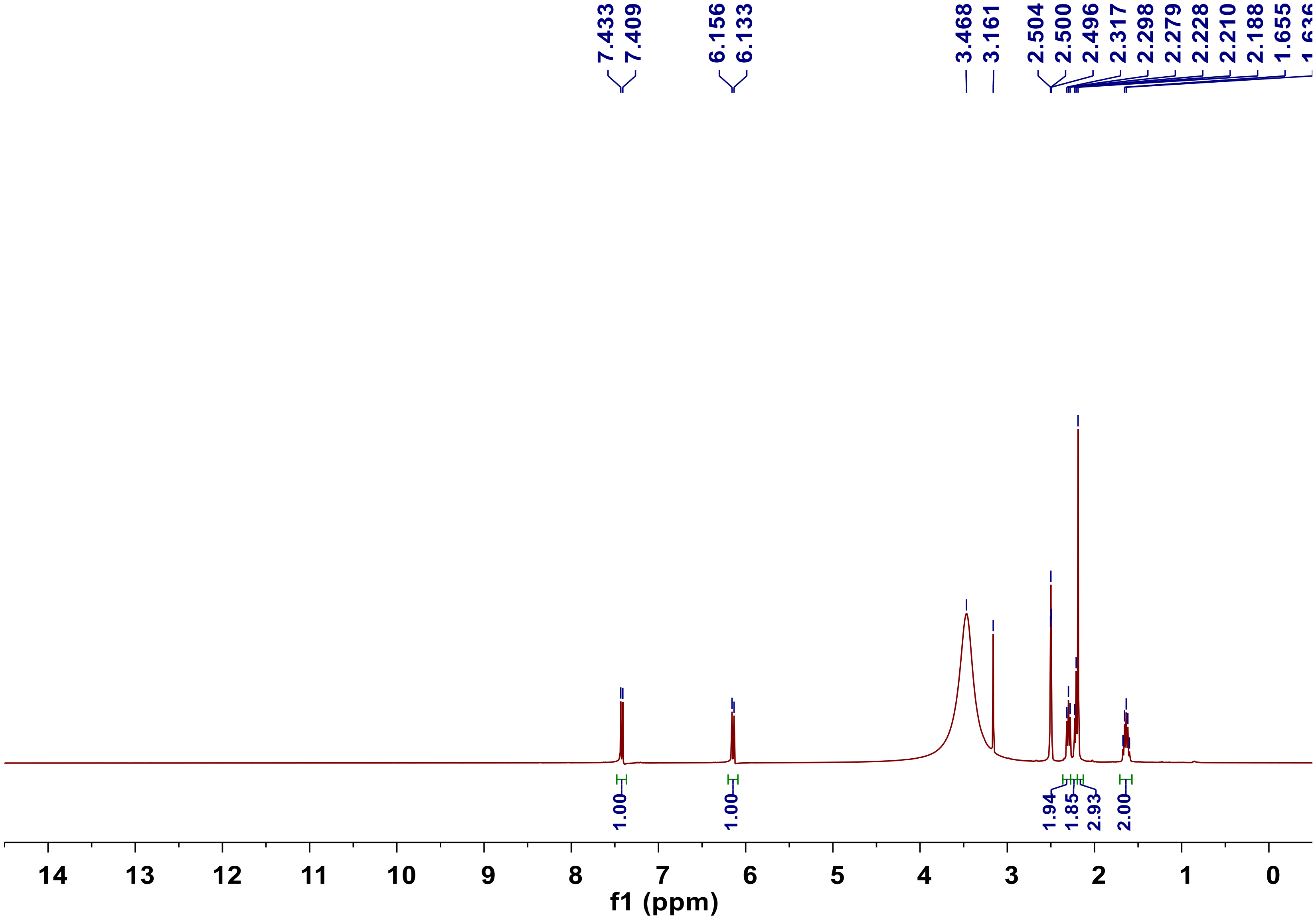

Figure S43 ^{13}C NMR spectrum of **9** in DMSO-d_6 (100 MHz)

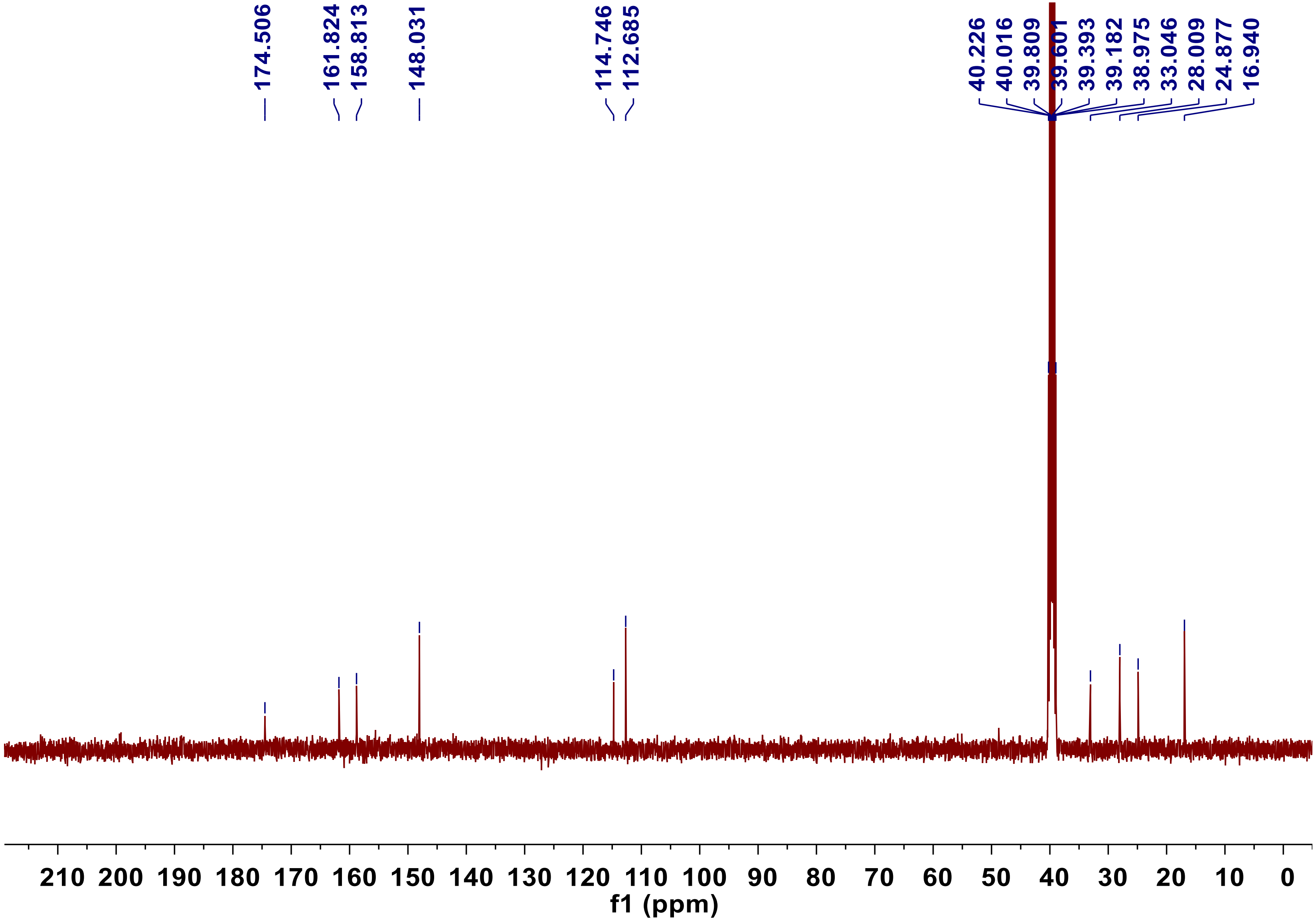

Figure S44 DEPT-90 spectrum of **9**

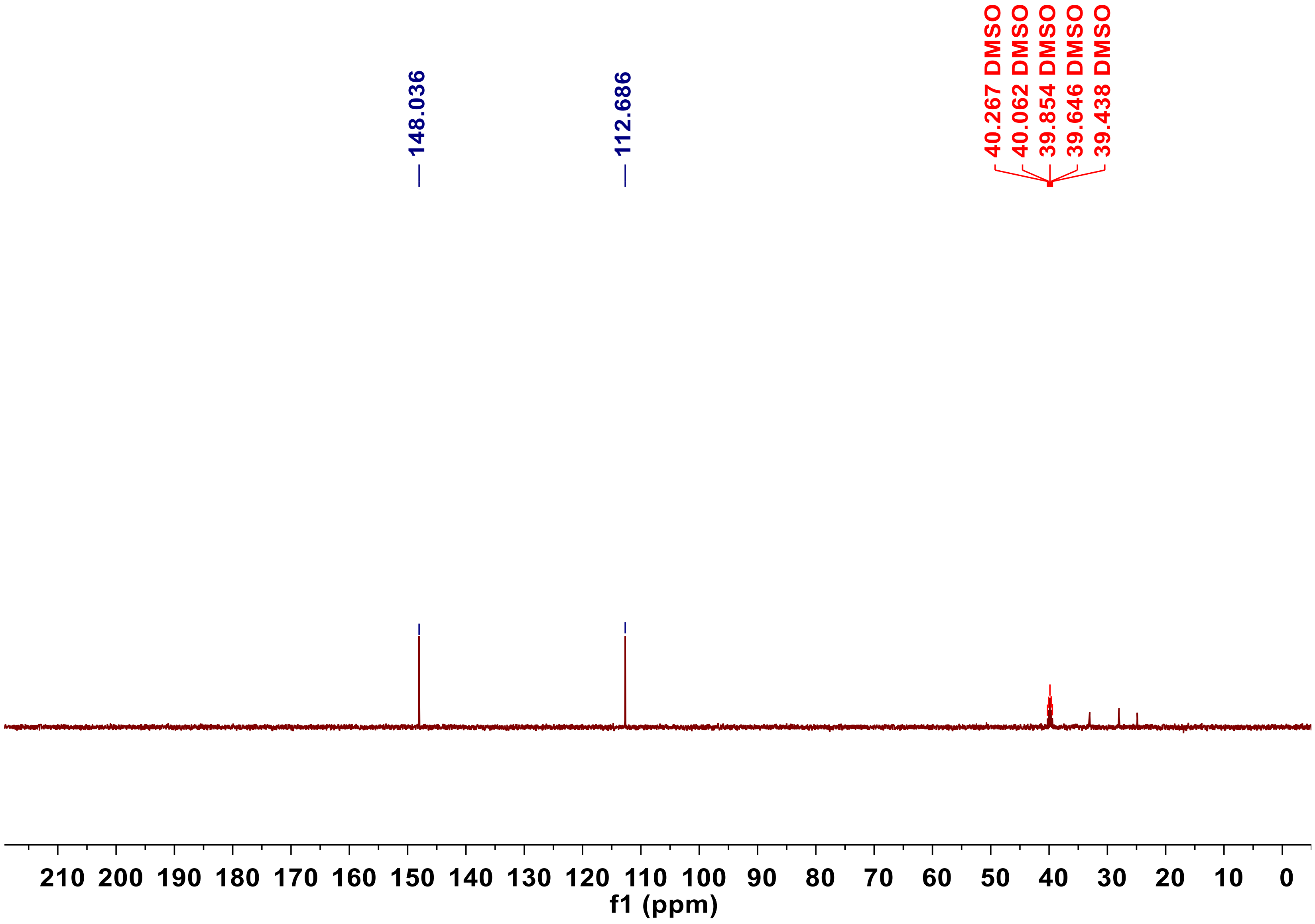

Figure S45 DEPT-135 spectrum of **9**

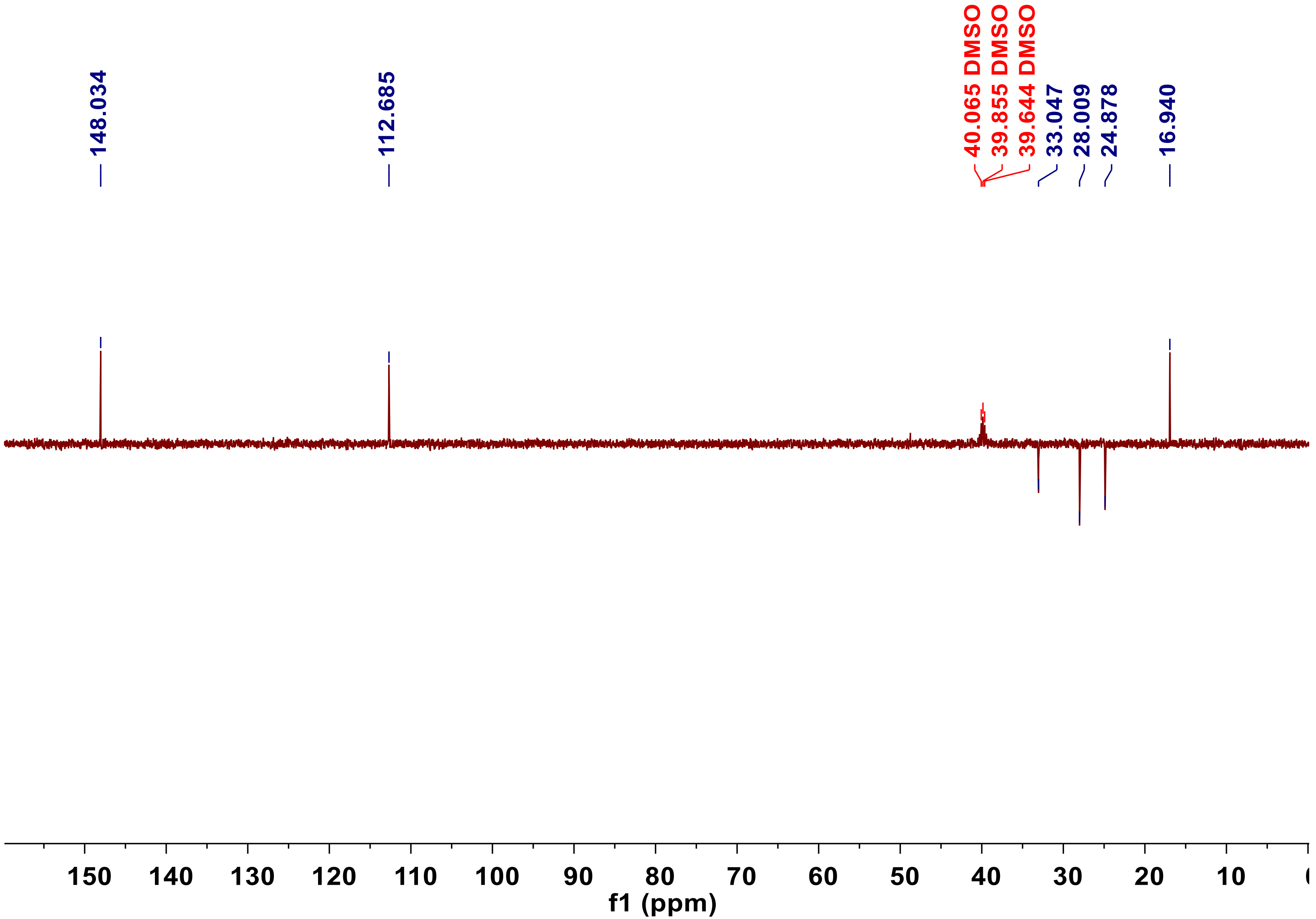

Figure S46 HSQC spectrum of **9**

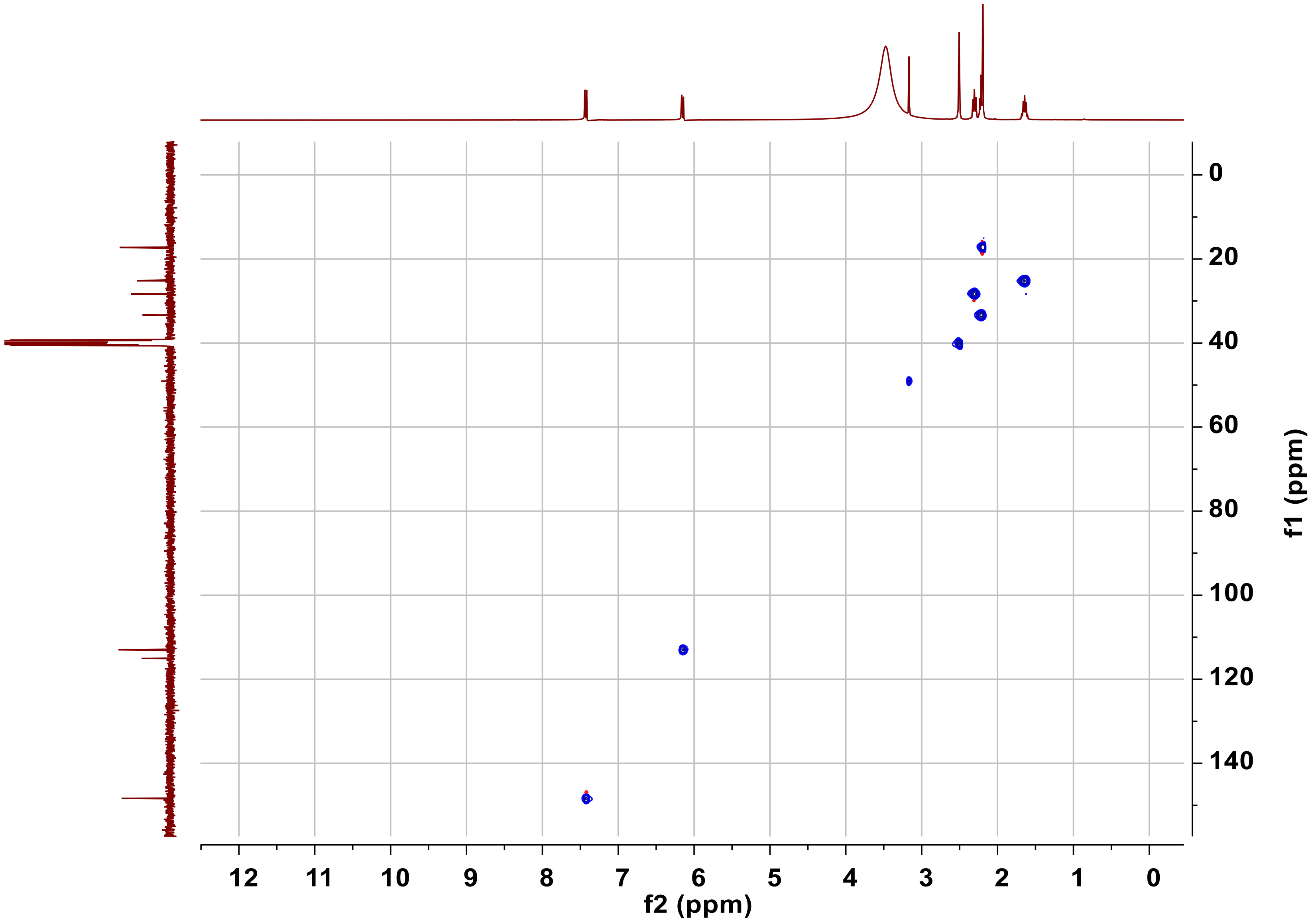

Figure S47 HMBC spectrum of **9**

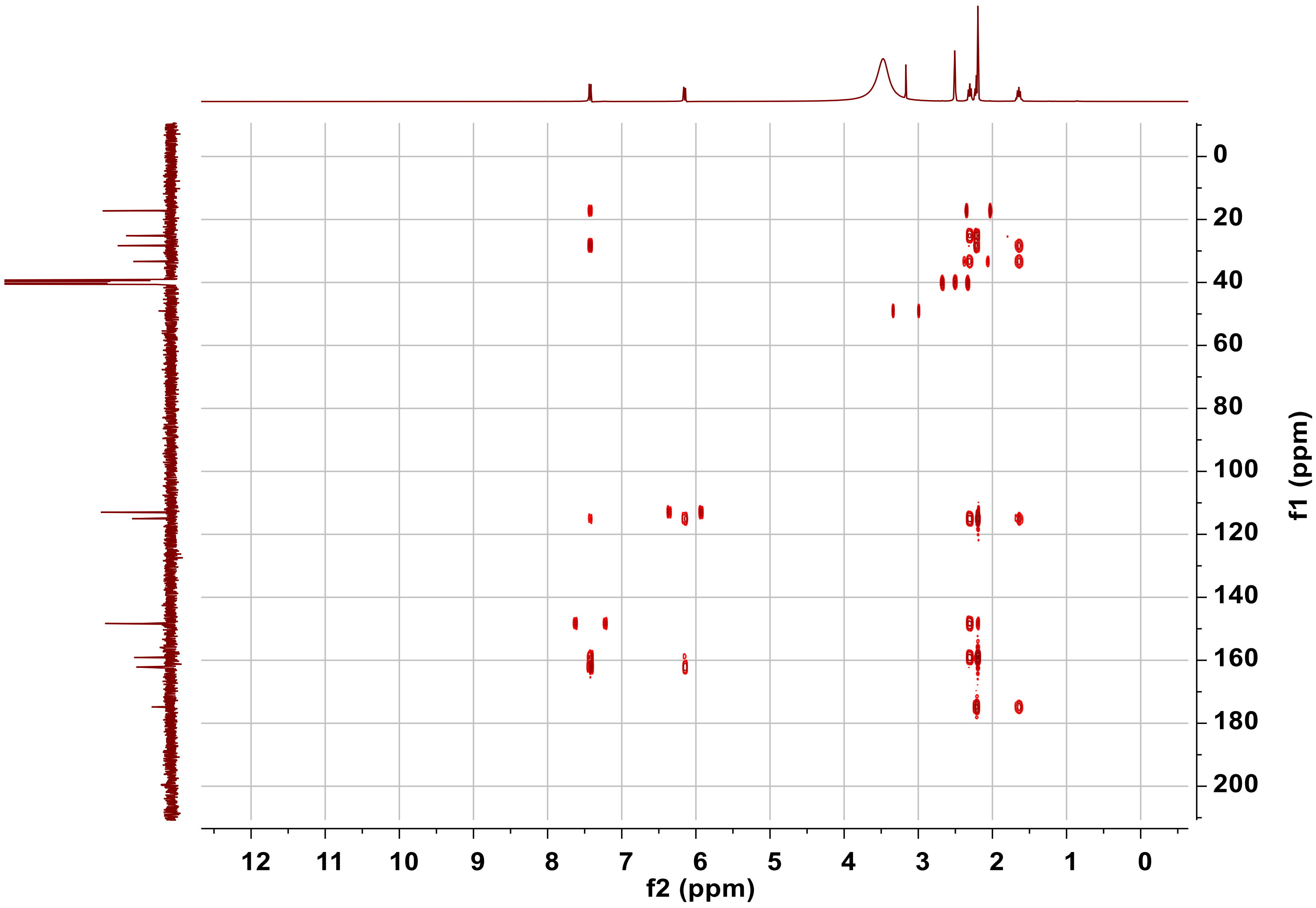

Figure S48 ^{1}H-^{1}H COSY spectrum of **9**

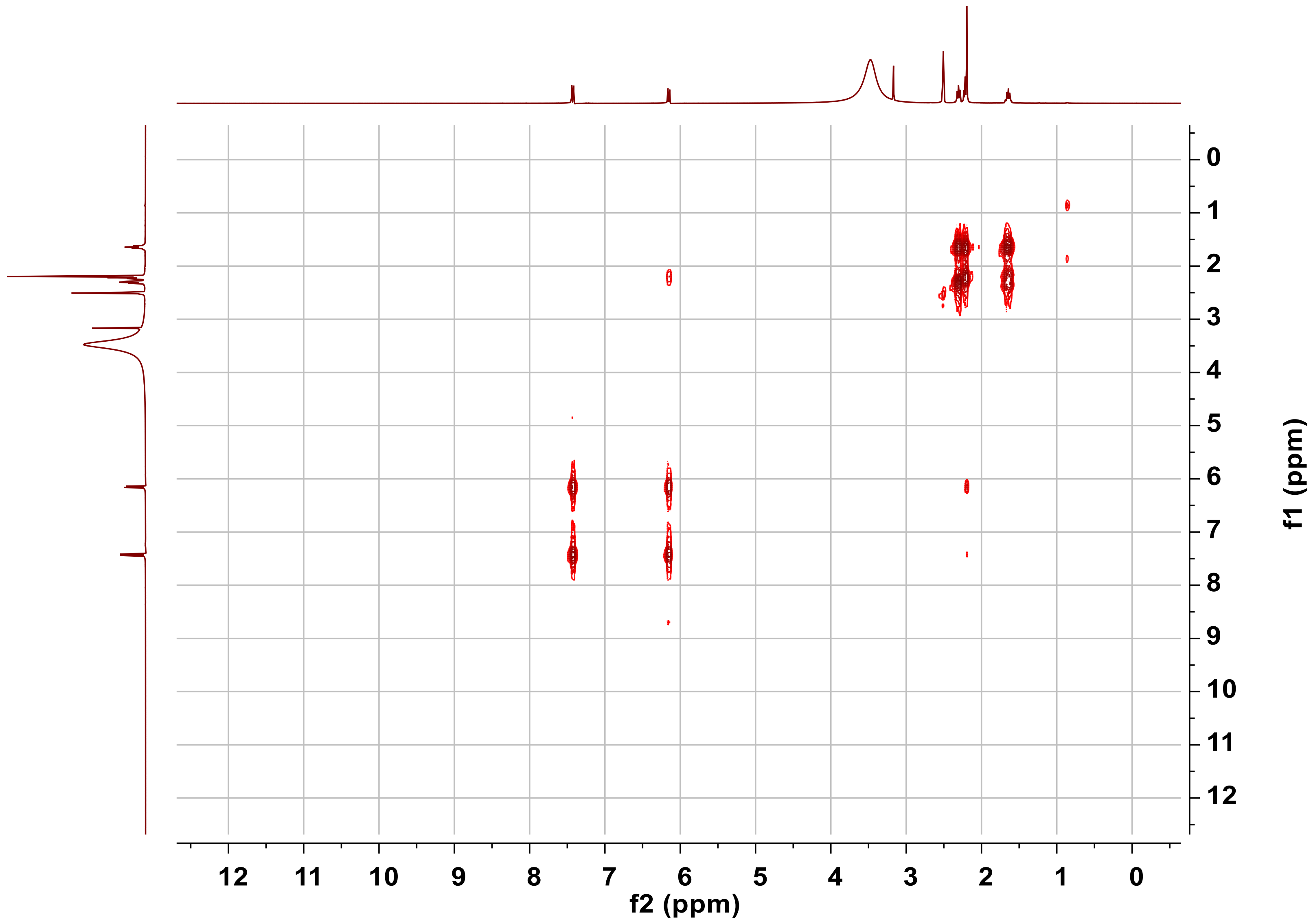

Figure S49 ^{1}H NMR spectrum of **10** in CD_3OD (500 MHz)

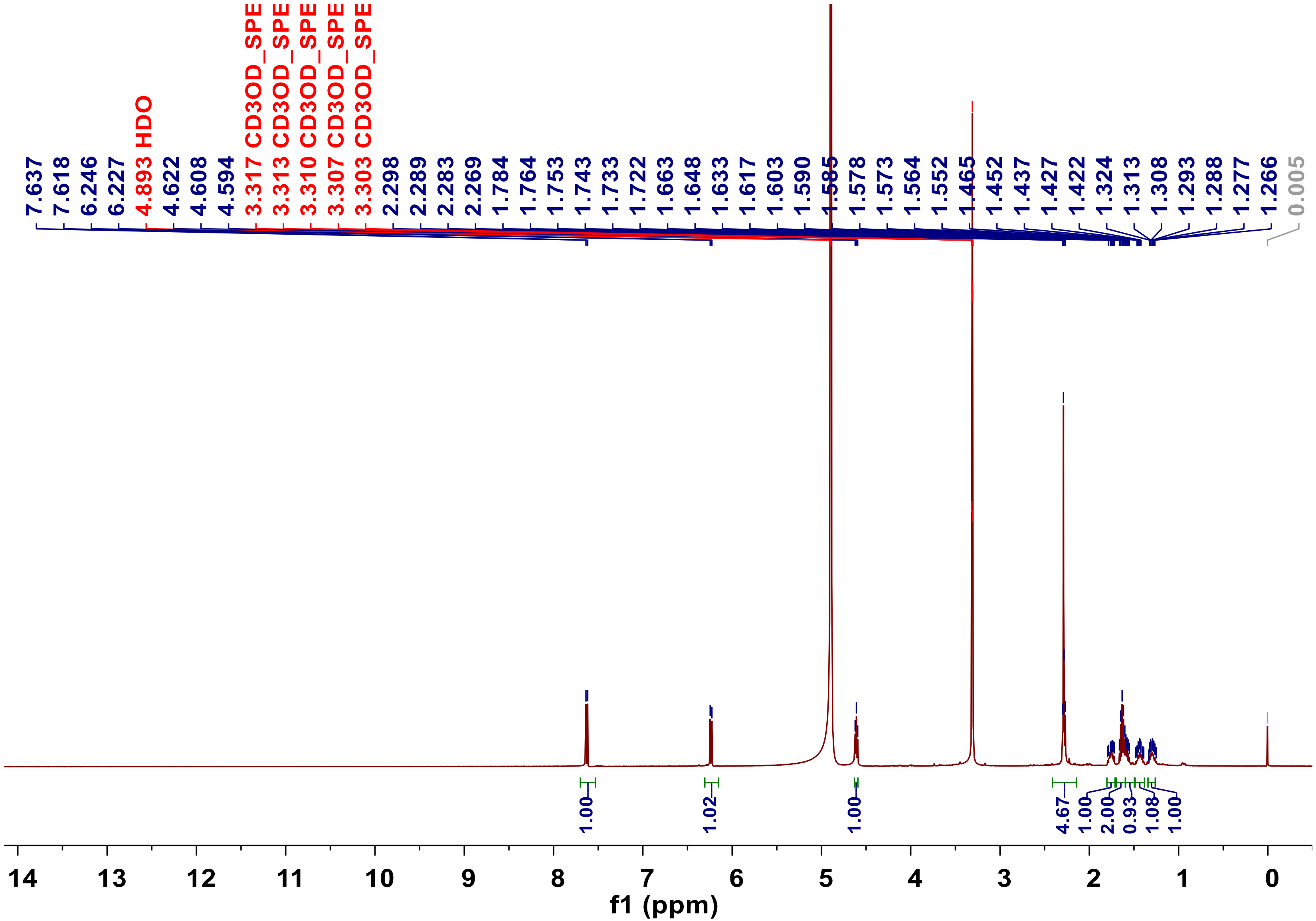

Figure S50 ^{13}C NMR spectrum of **10** in CD_3OD (125 MHz)

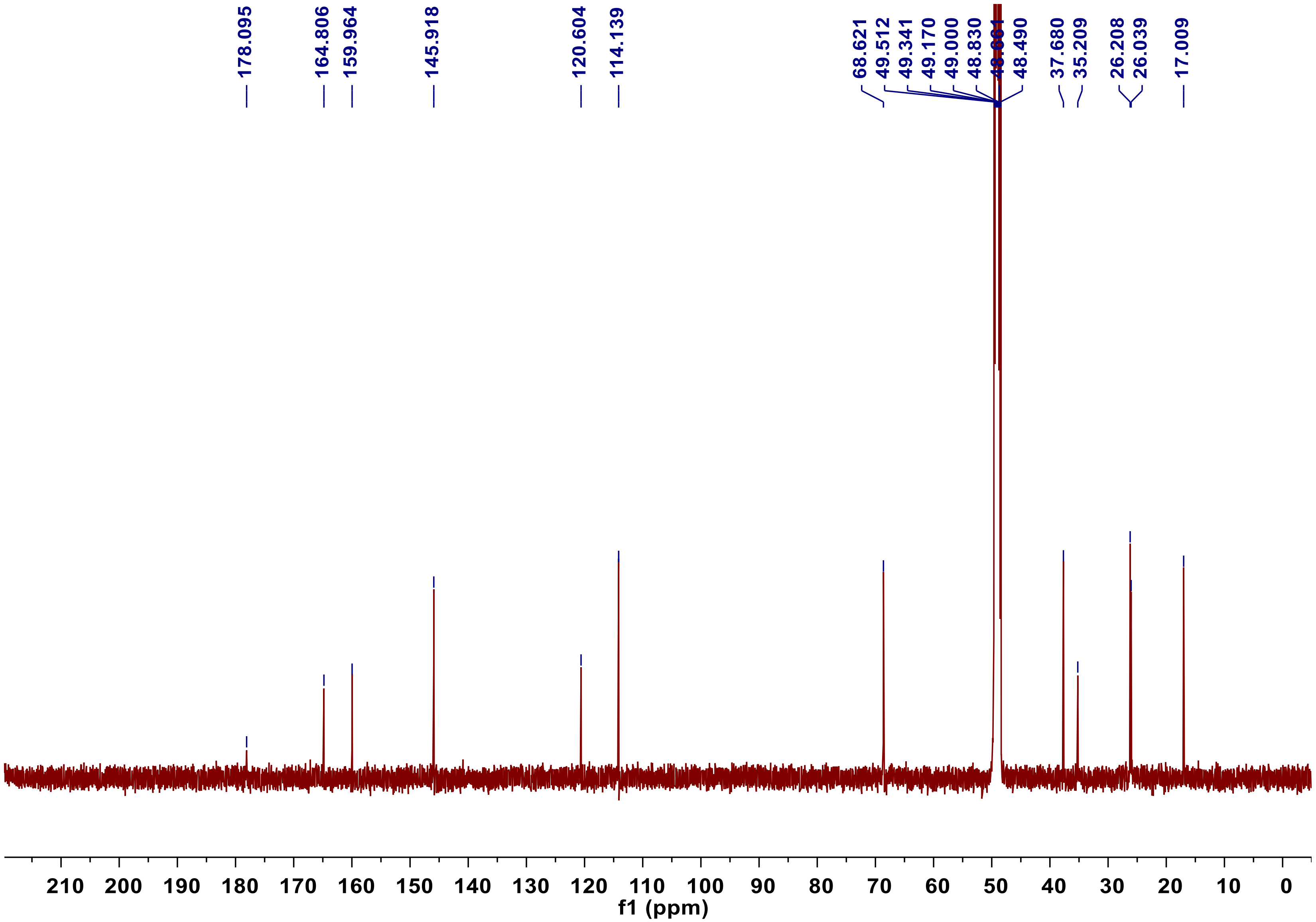

Figure S51 DEPT-90 spectrum of **10**

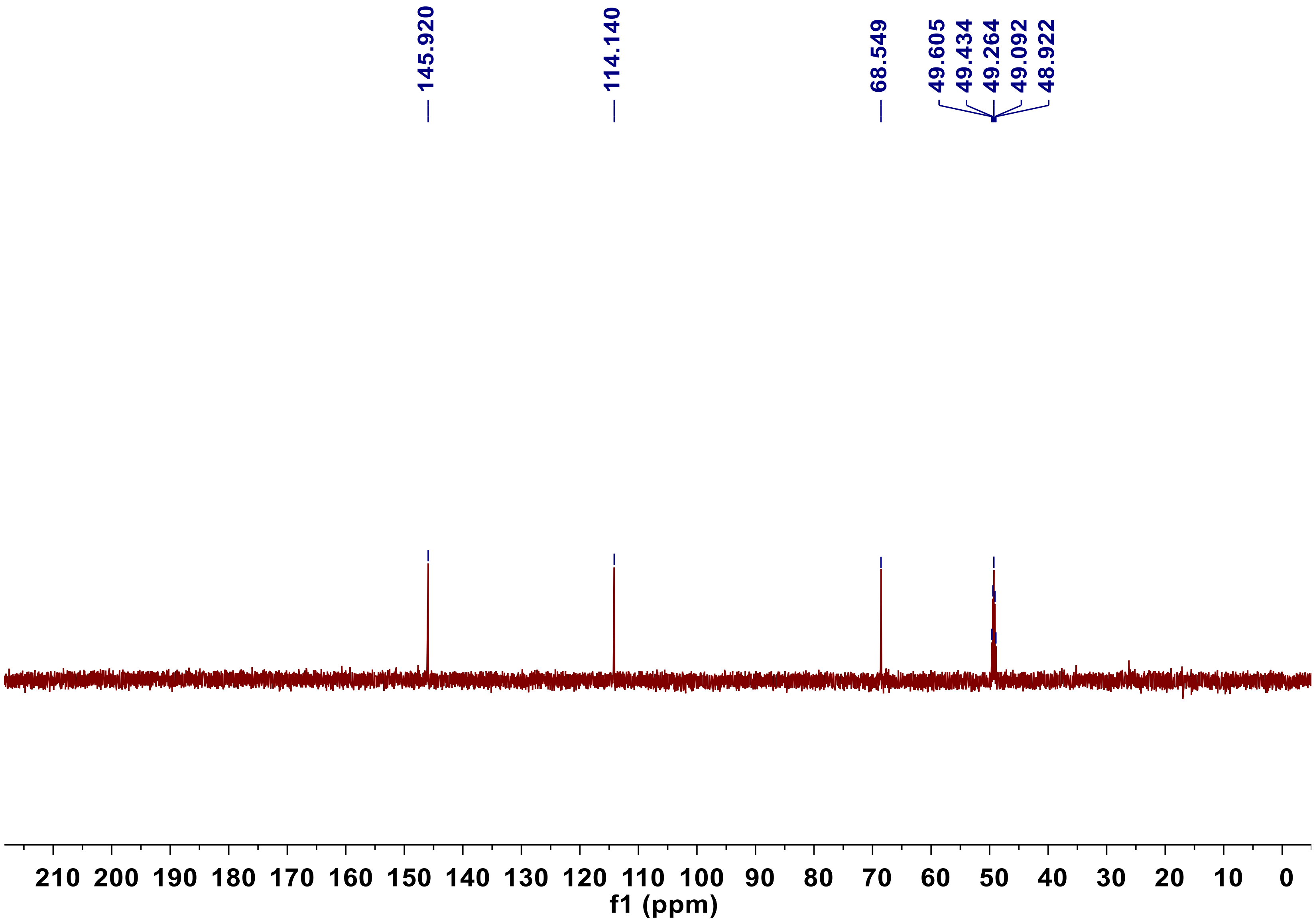

Figure S52 DEPT-135 spectrum of **10**

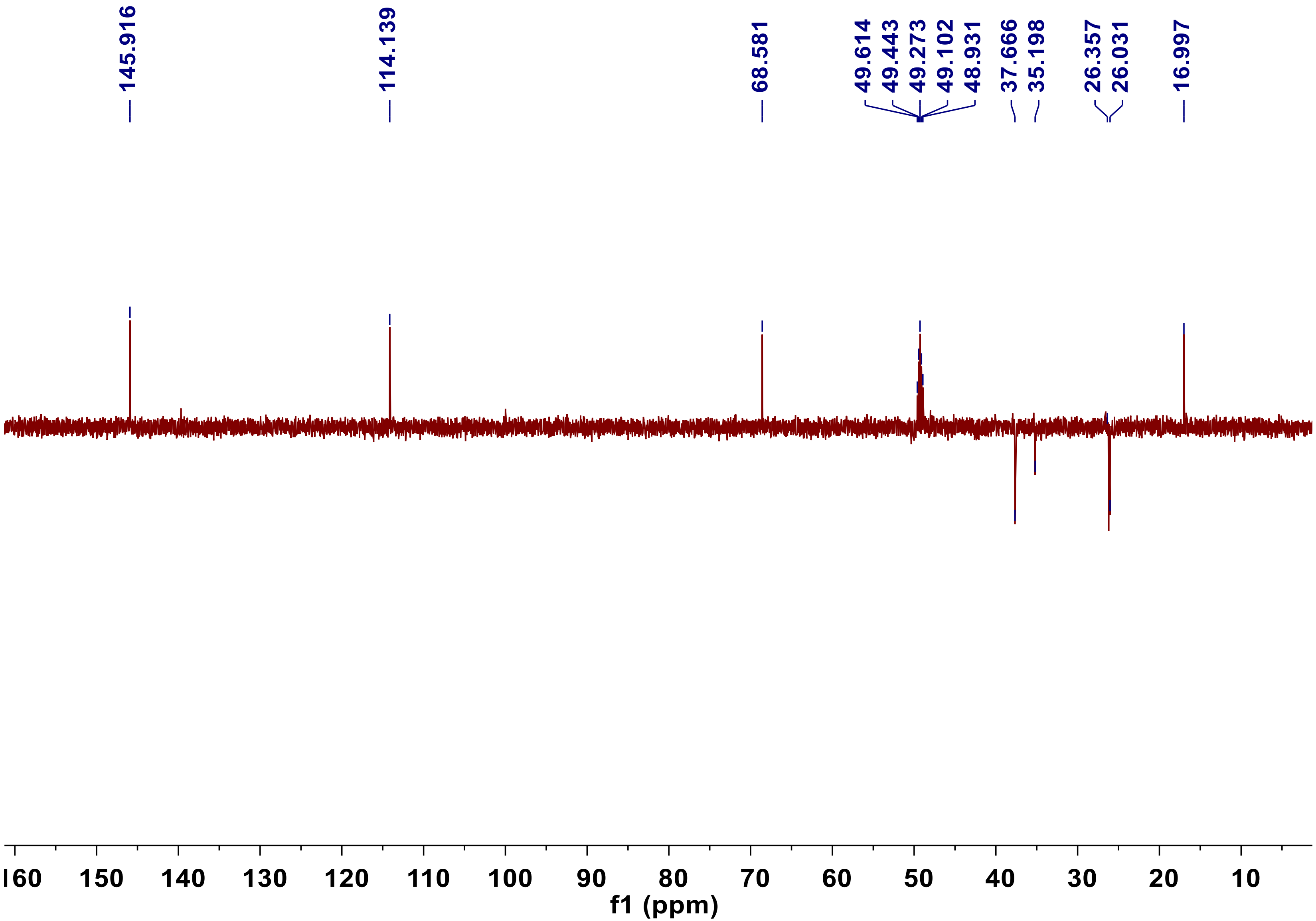

Figure S53 HSQC spectrum of **10**

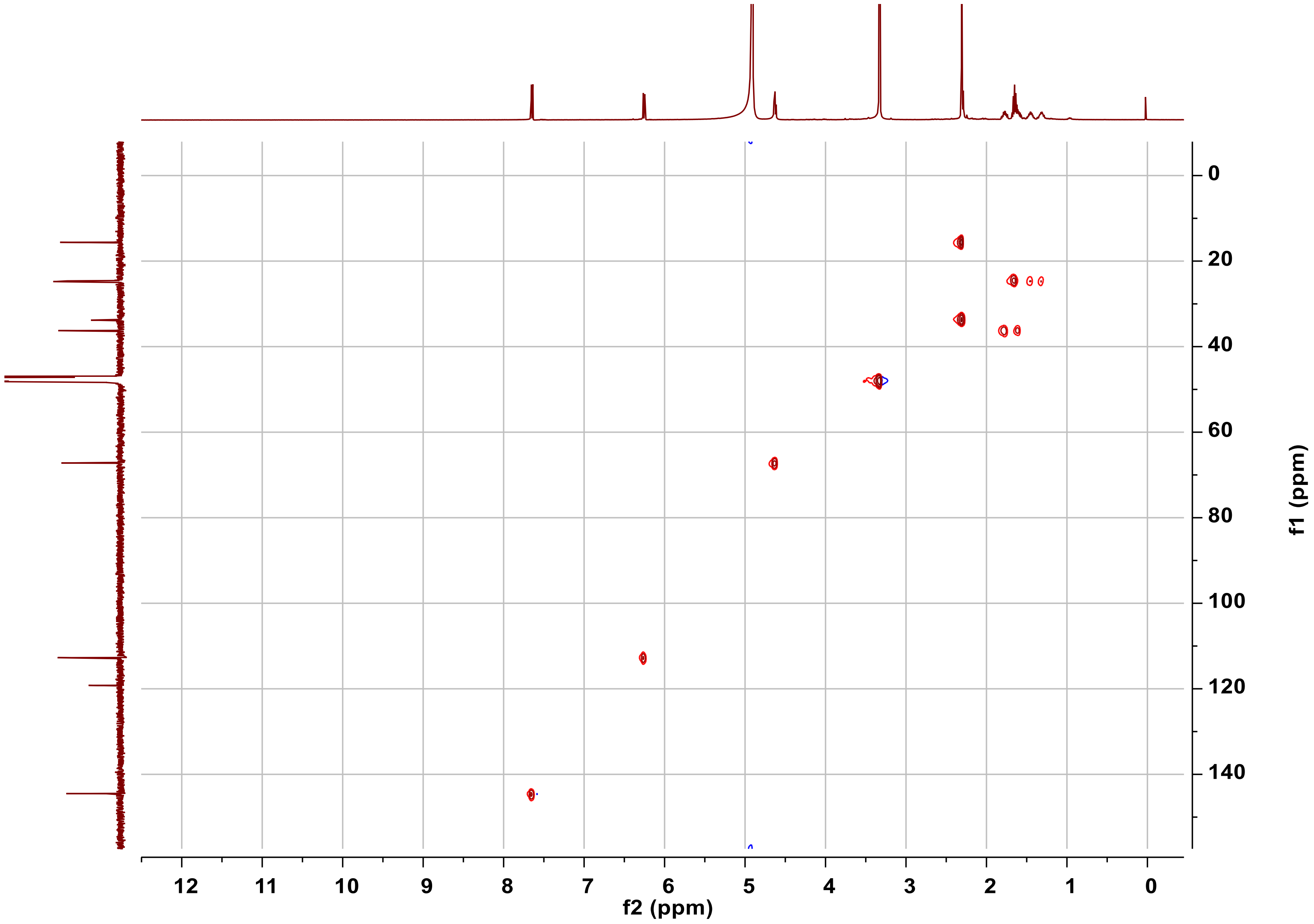

Figure S54 HMBC spectrum of **10**

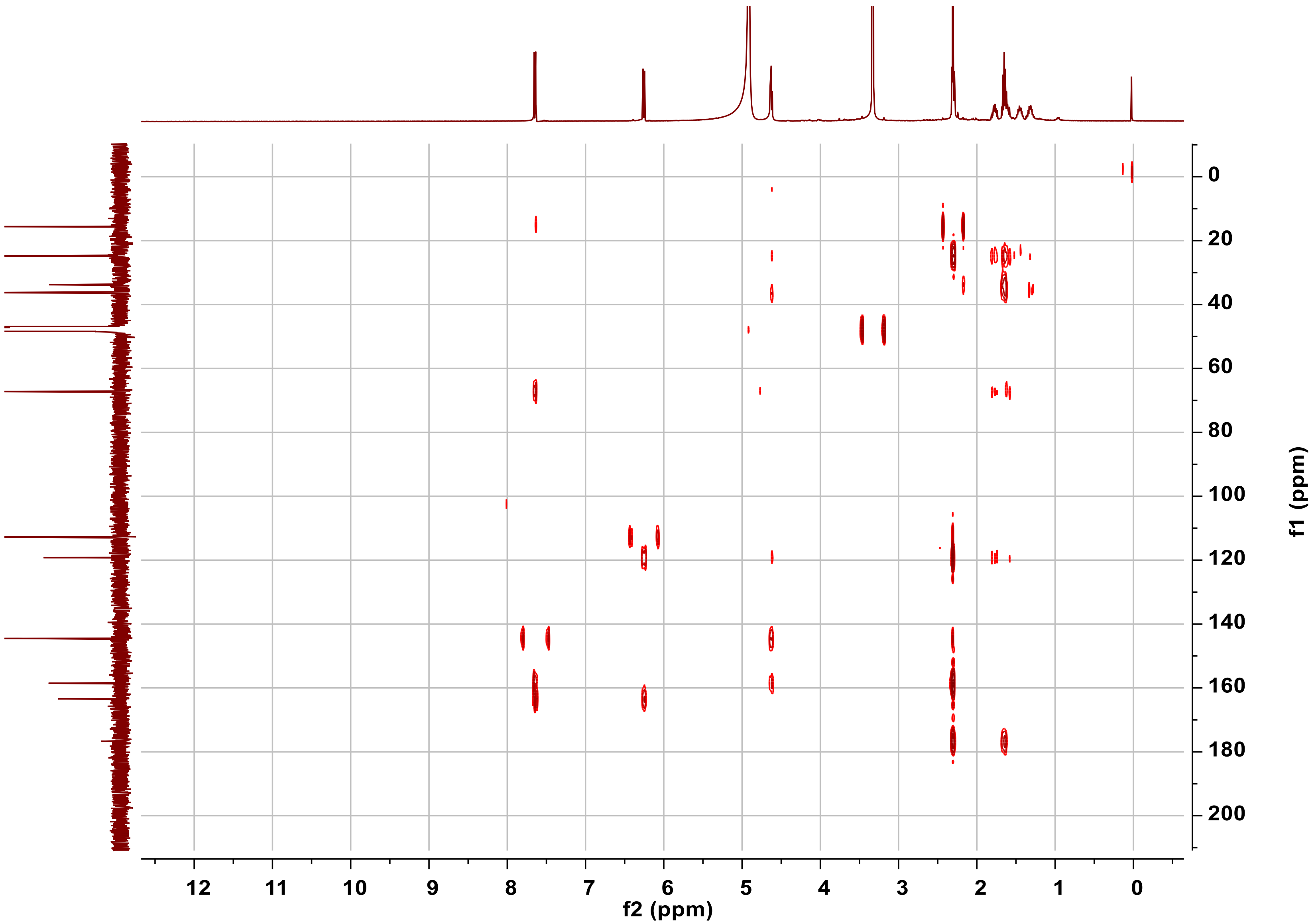

Figure S55 ^{1}H-^{1}H COSY spectrum of **10**

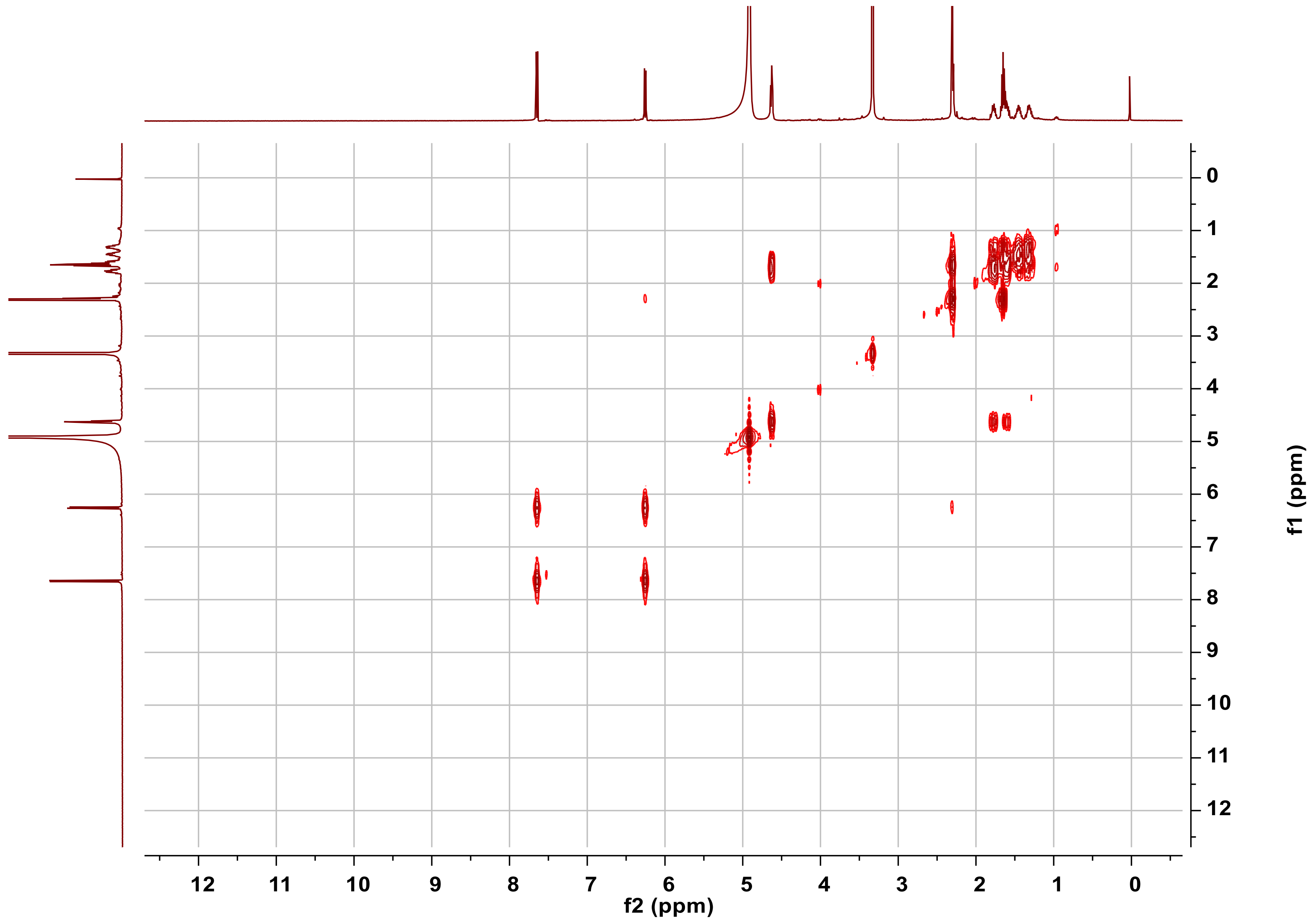

Figure S56 NOESY spectrum of **10**

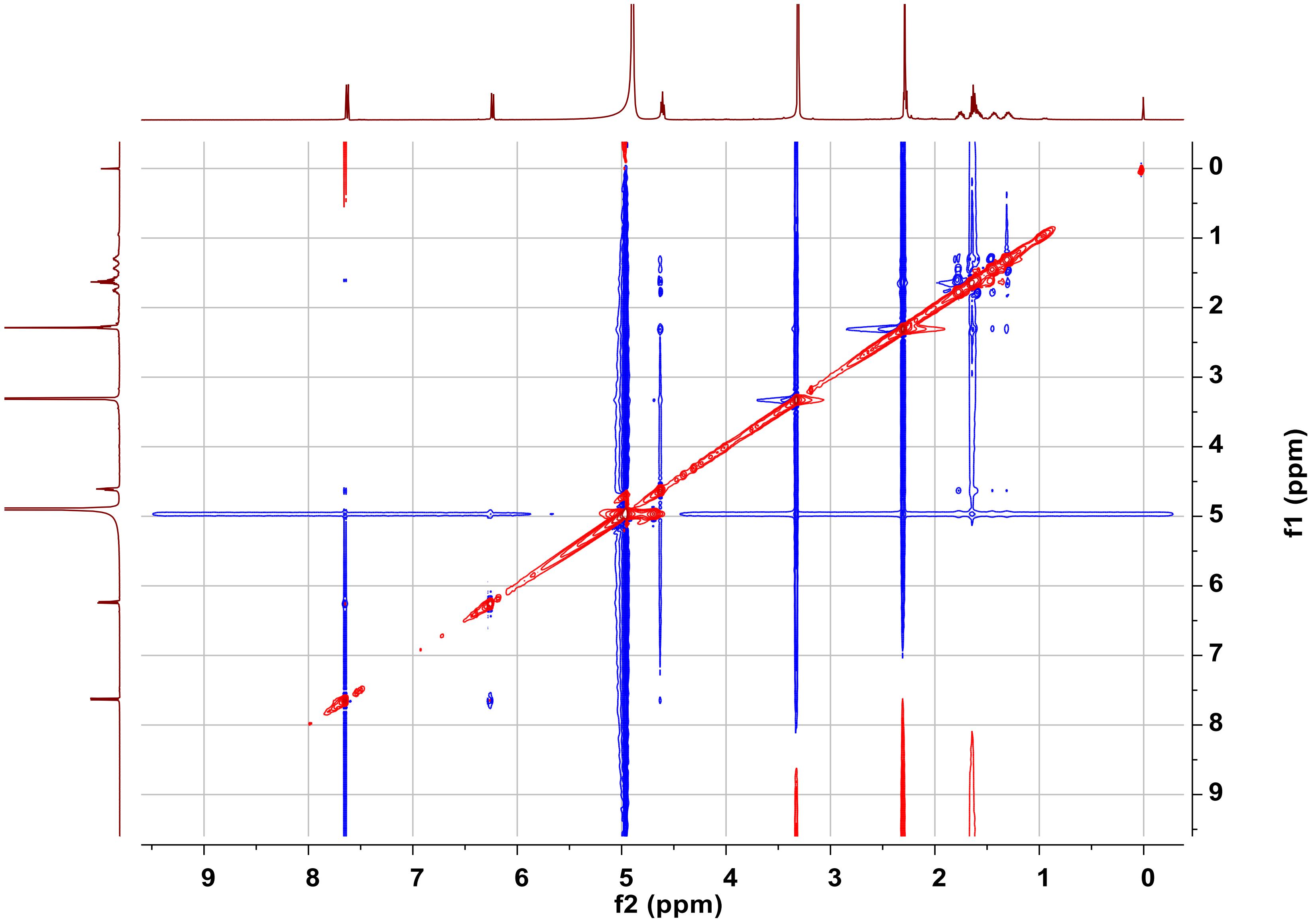

Figure S57 ^{1}H NMR spectrum of **11** in CD_3OD (400 MHz)

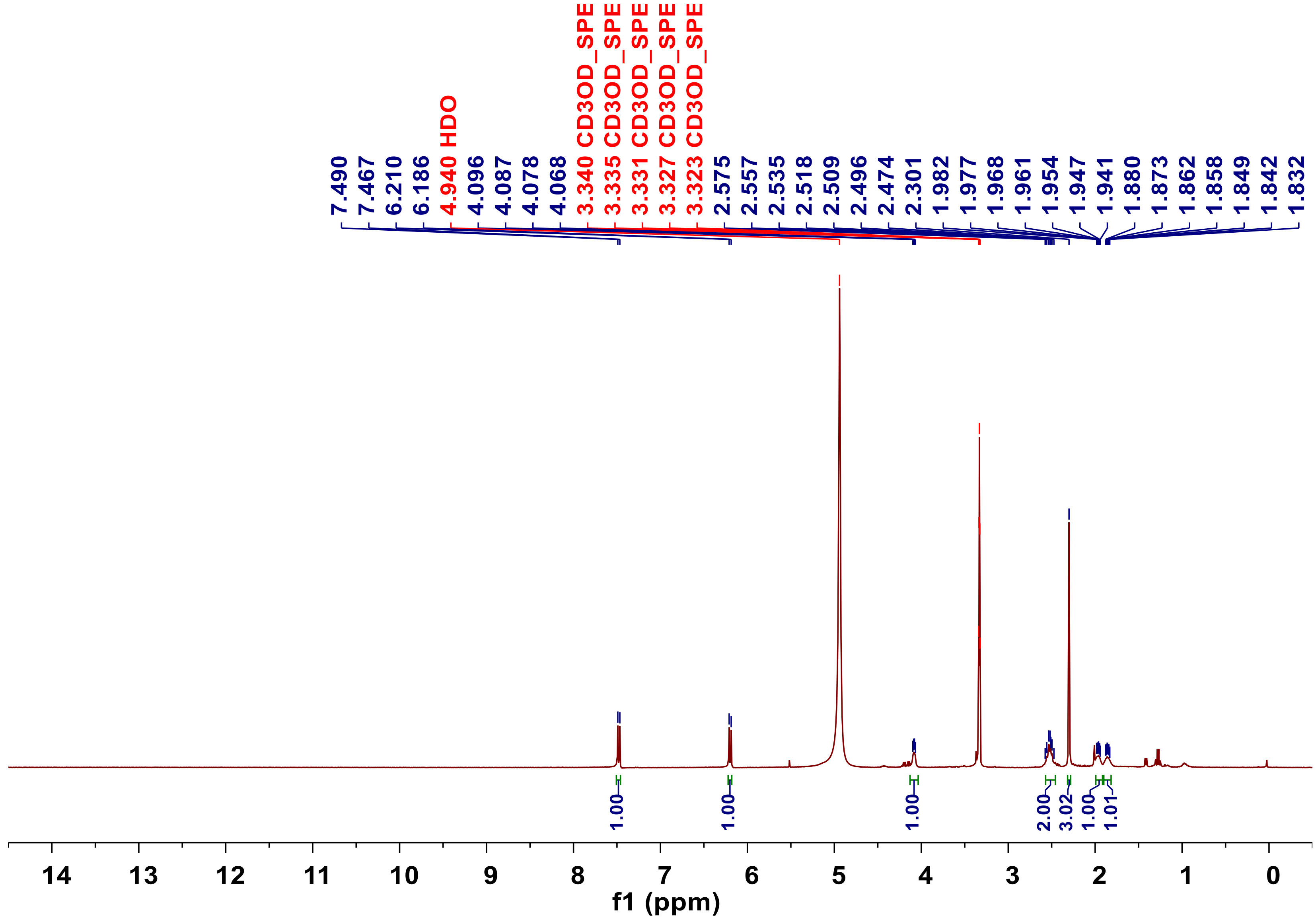

Figure S58 ^{13}C NMR spectrum of **11** in CD_3OD (100 MHz)

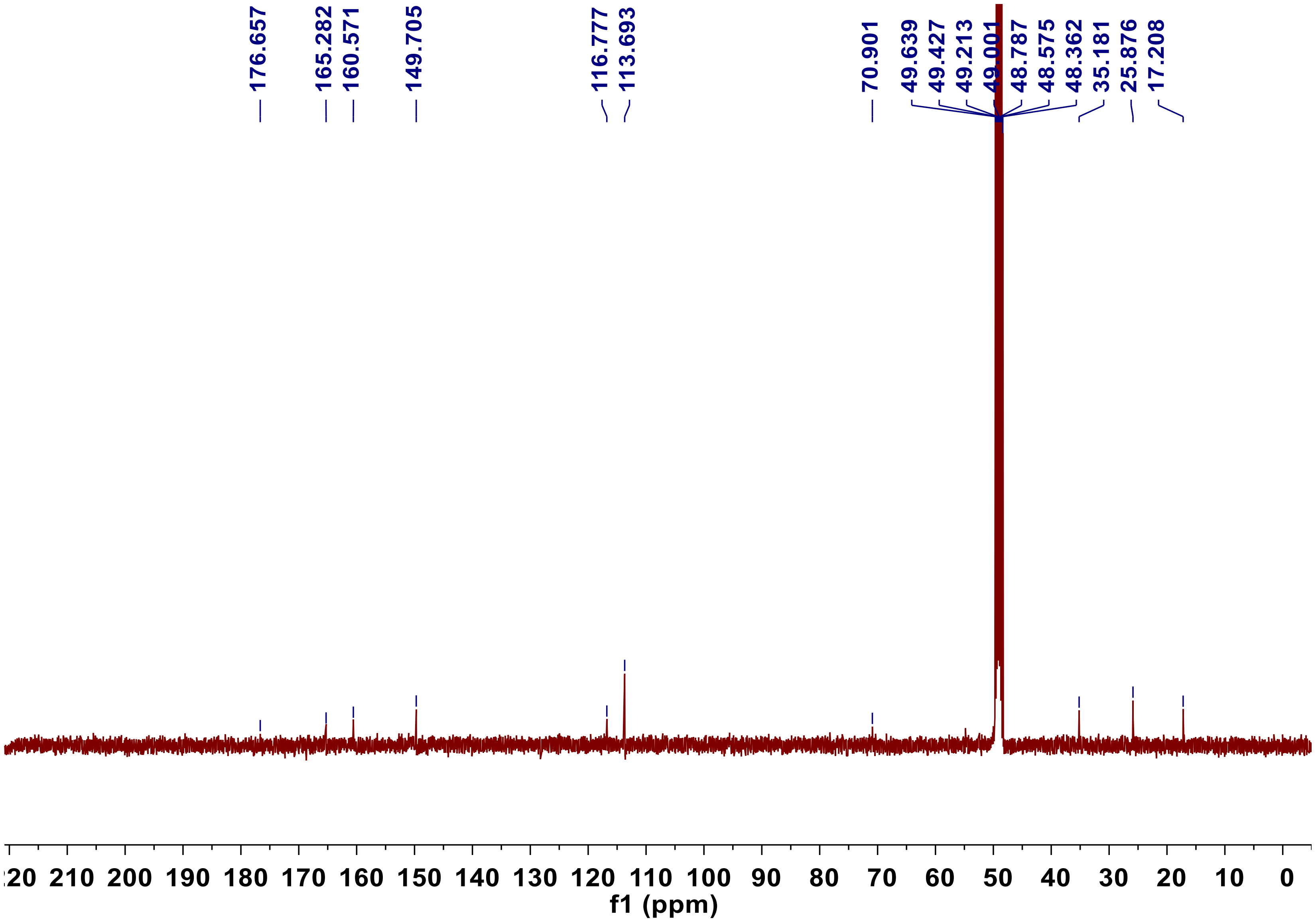

Figure S59 DEPT-90 spectrum of **11**

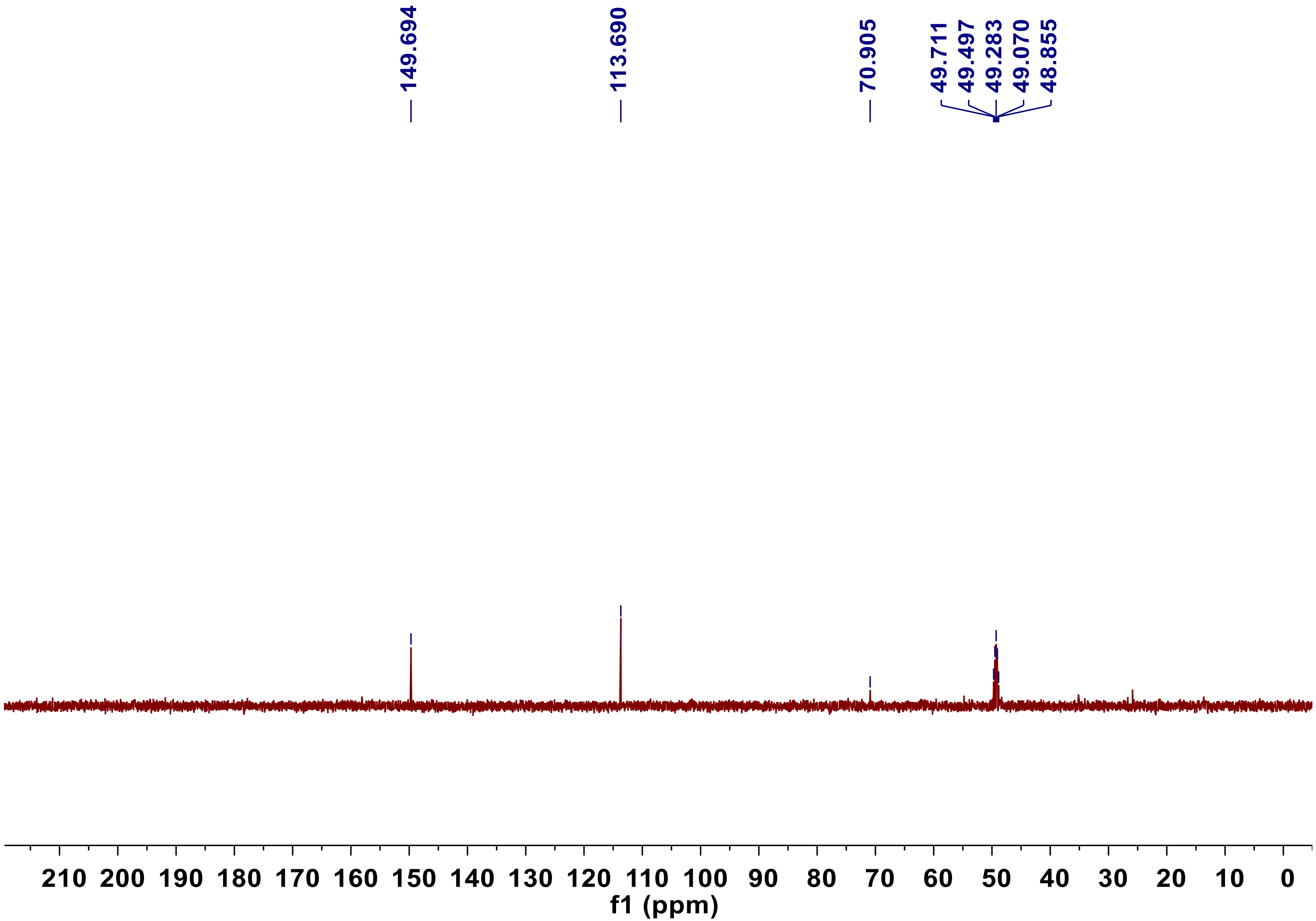

Figure S60 DEPT-135 spectrum of **11**

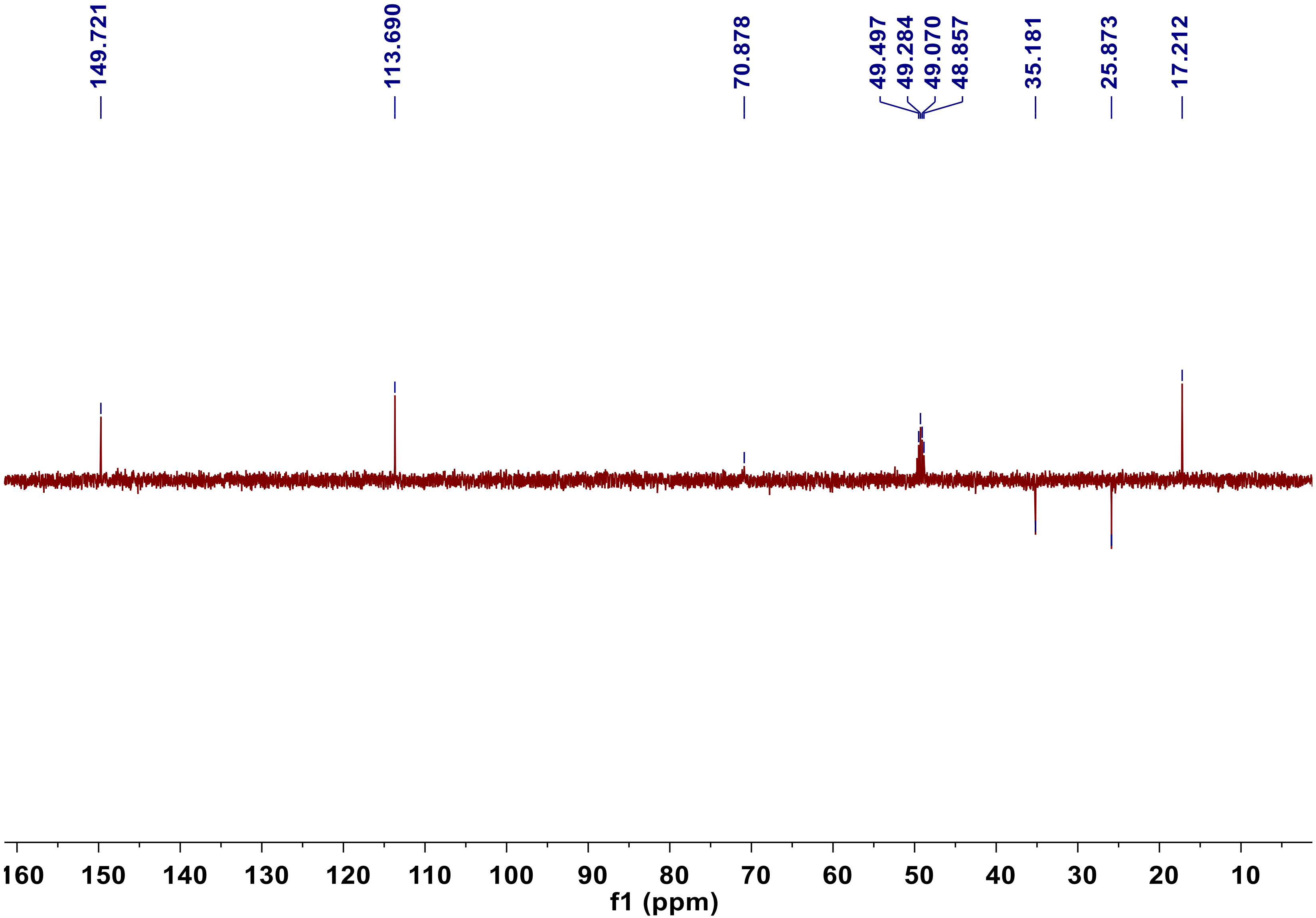

Figure S61 HSQC spectrum of **11**

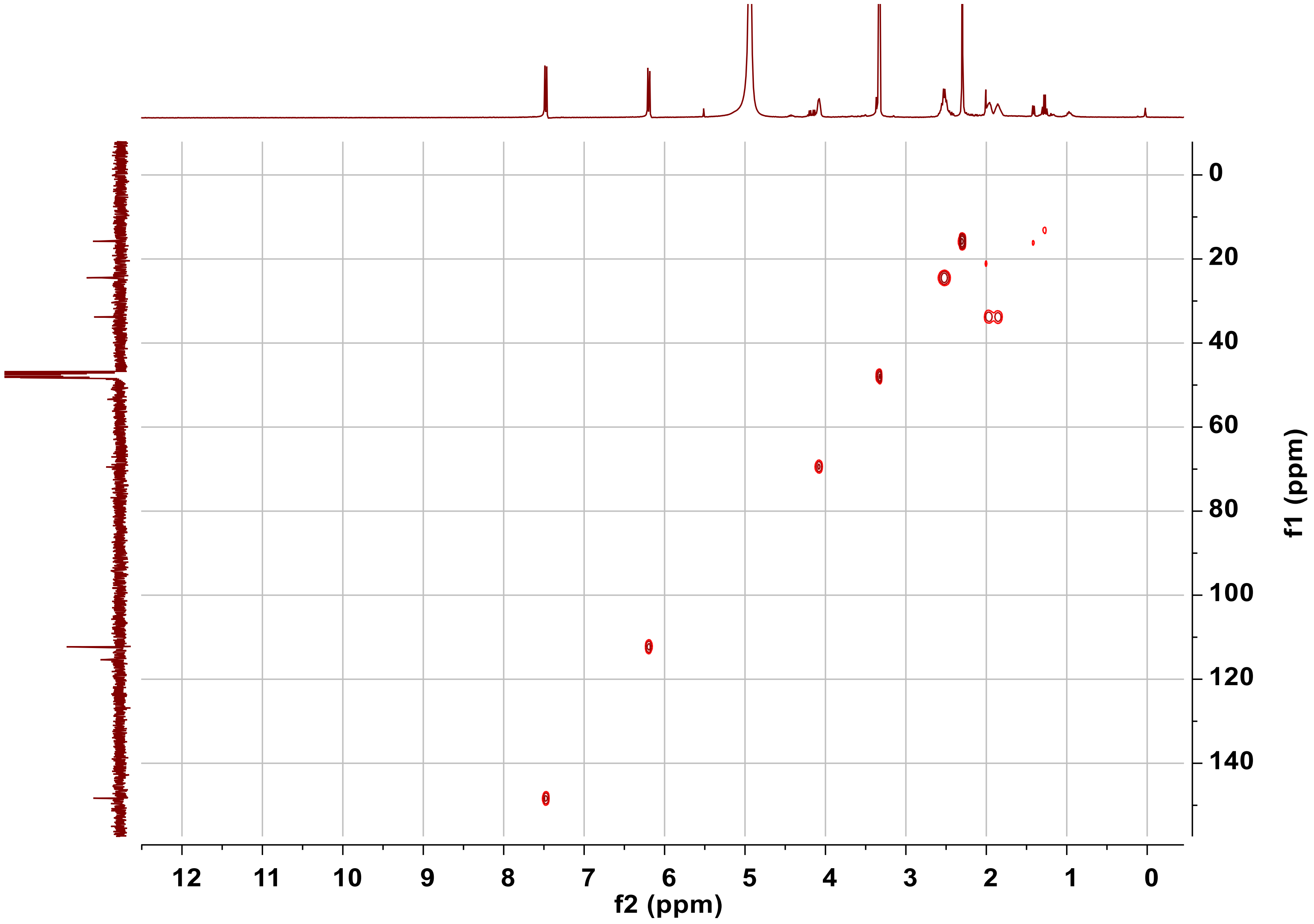

Figure S62 HMBC spectrum of **11**

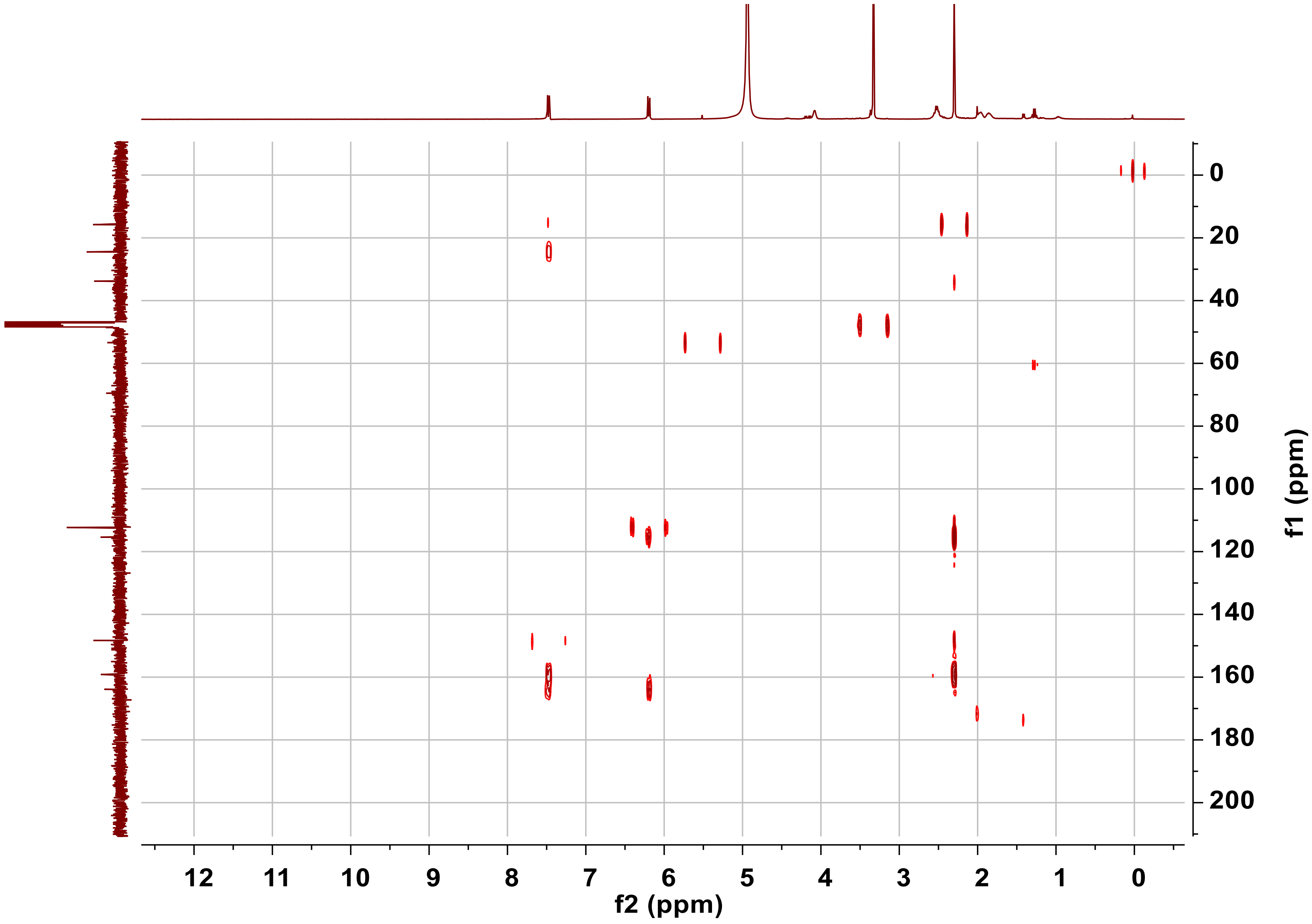

Figure S63 ^{1}H-^{1}H COSY spectrum of **11**

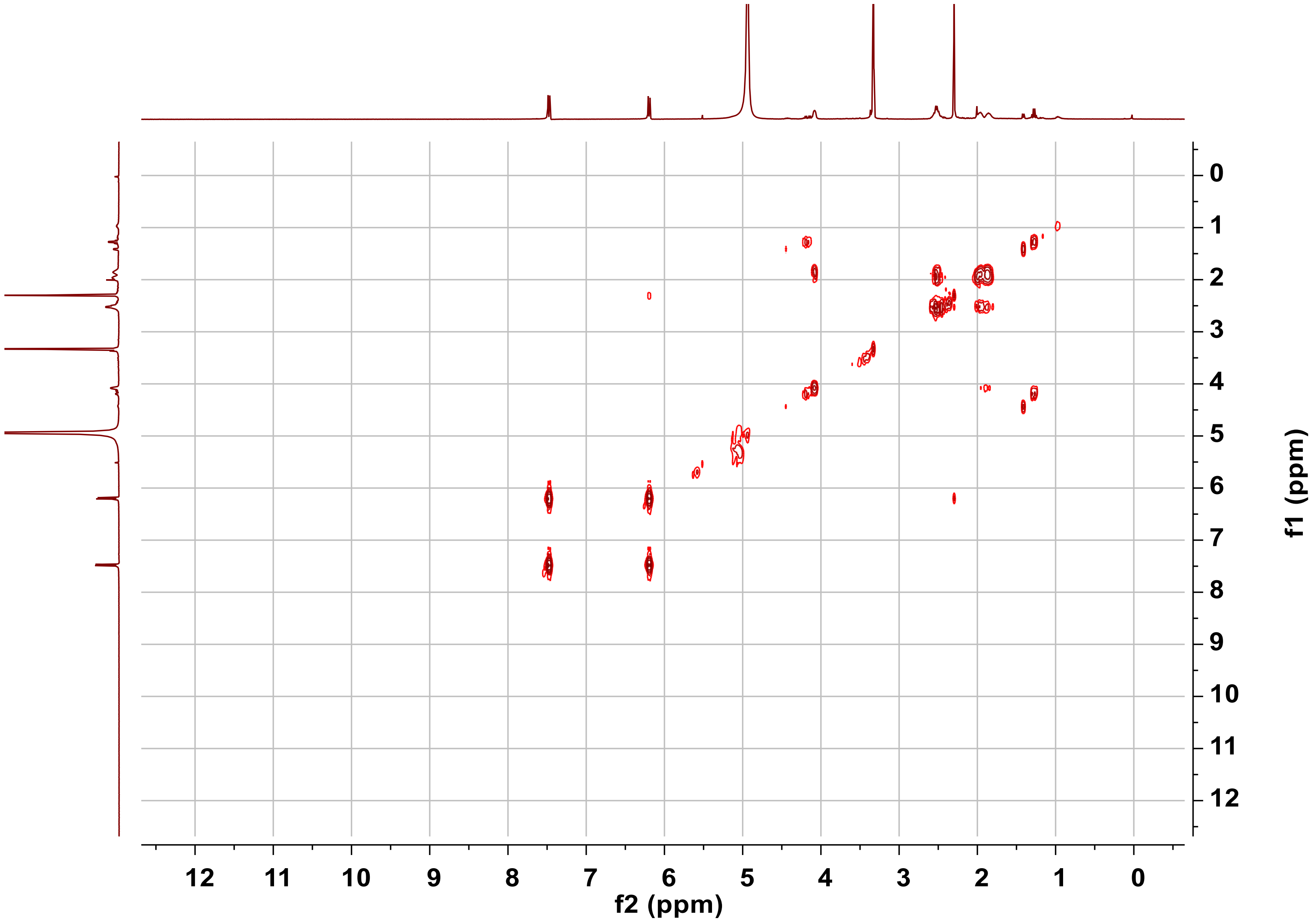

Figure S64 NOESY spectrum of **11**

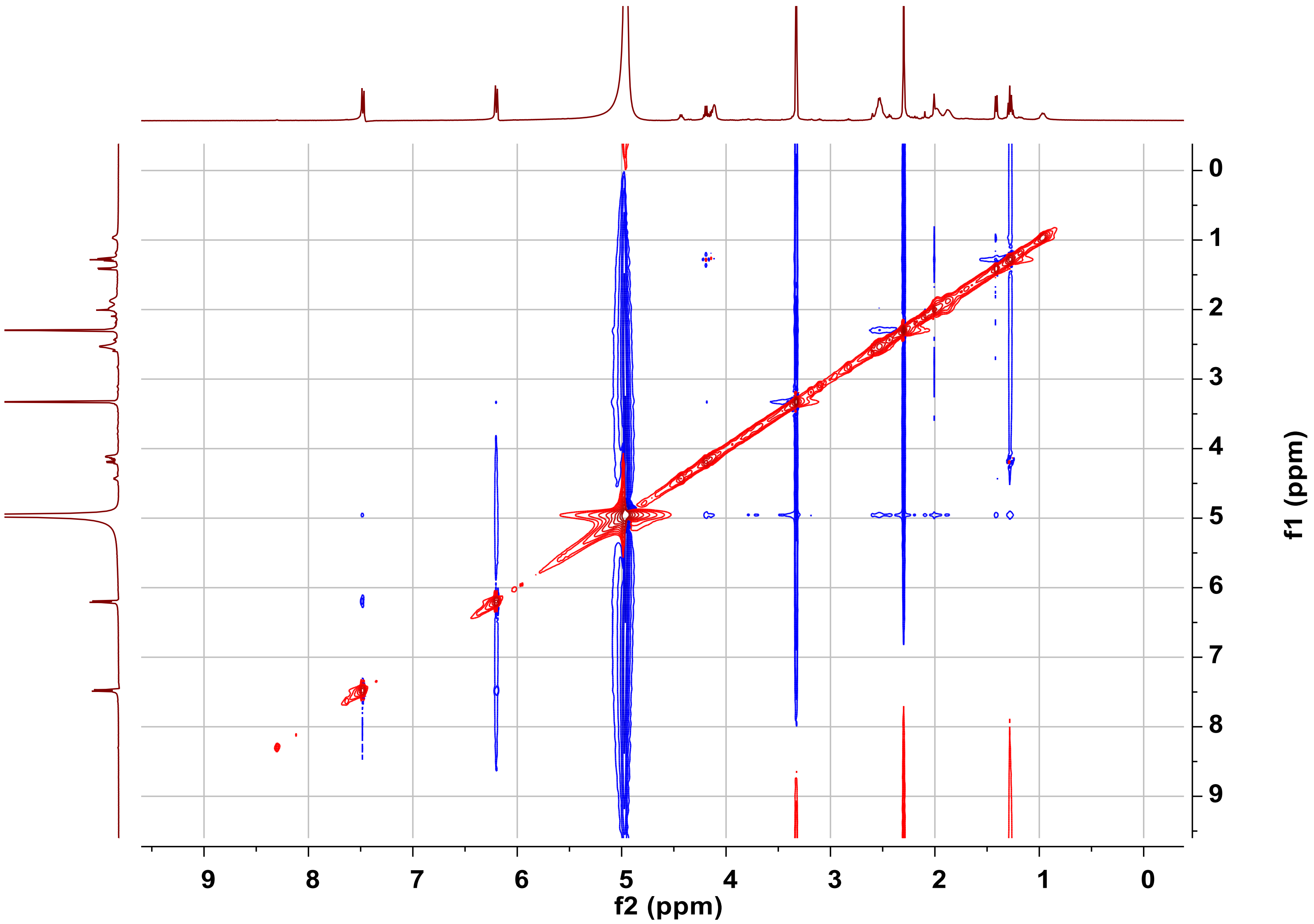

Figure S65 ^{1}H NMR spectrum of **12** in CD_3OD (500 MHz)

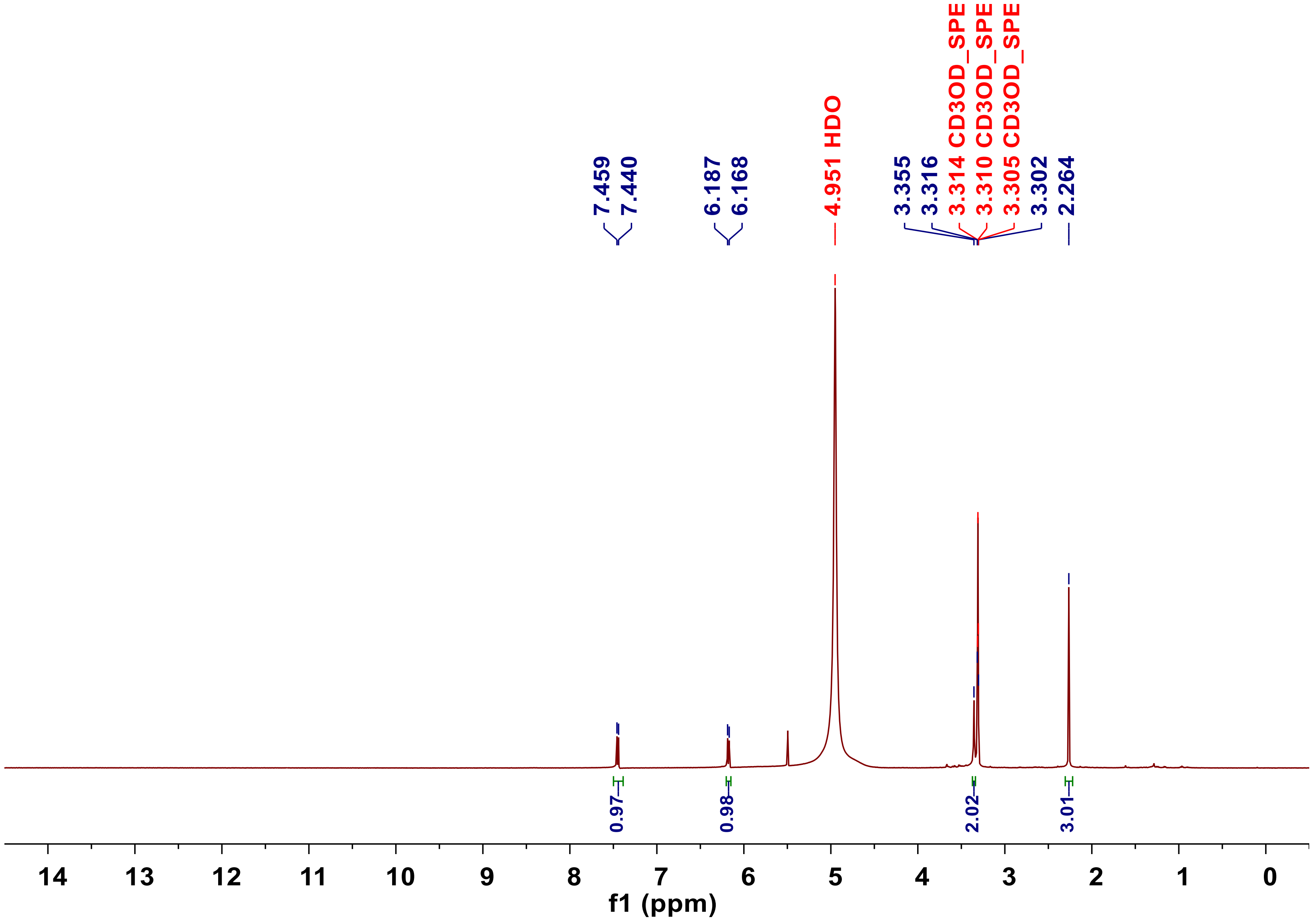

Figure S66 ^{13}C NMR spectrum of **12** in CD_3OD (125 MHz)

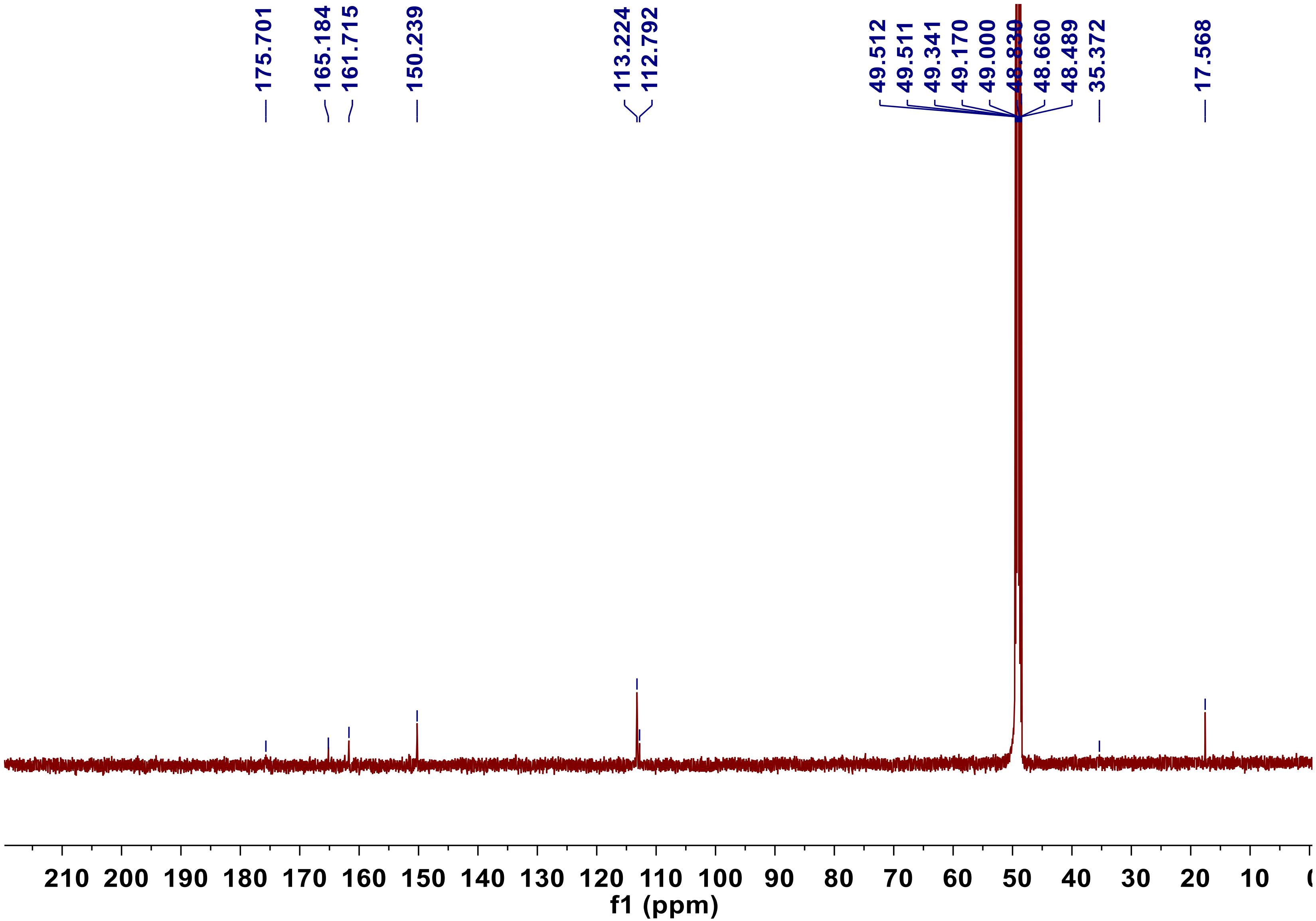

Figure S67 DEPT-90 spectrum of **12**

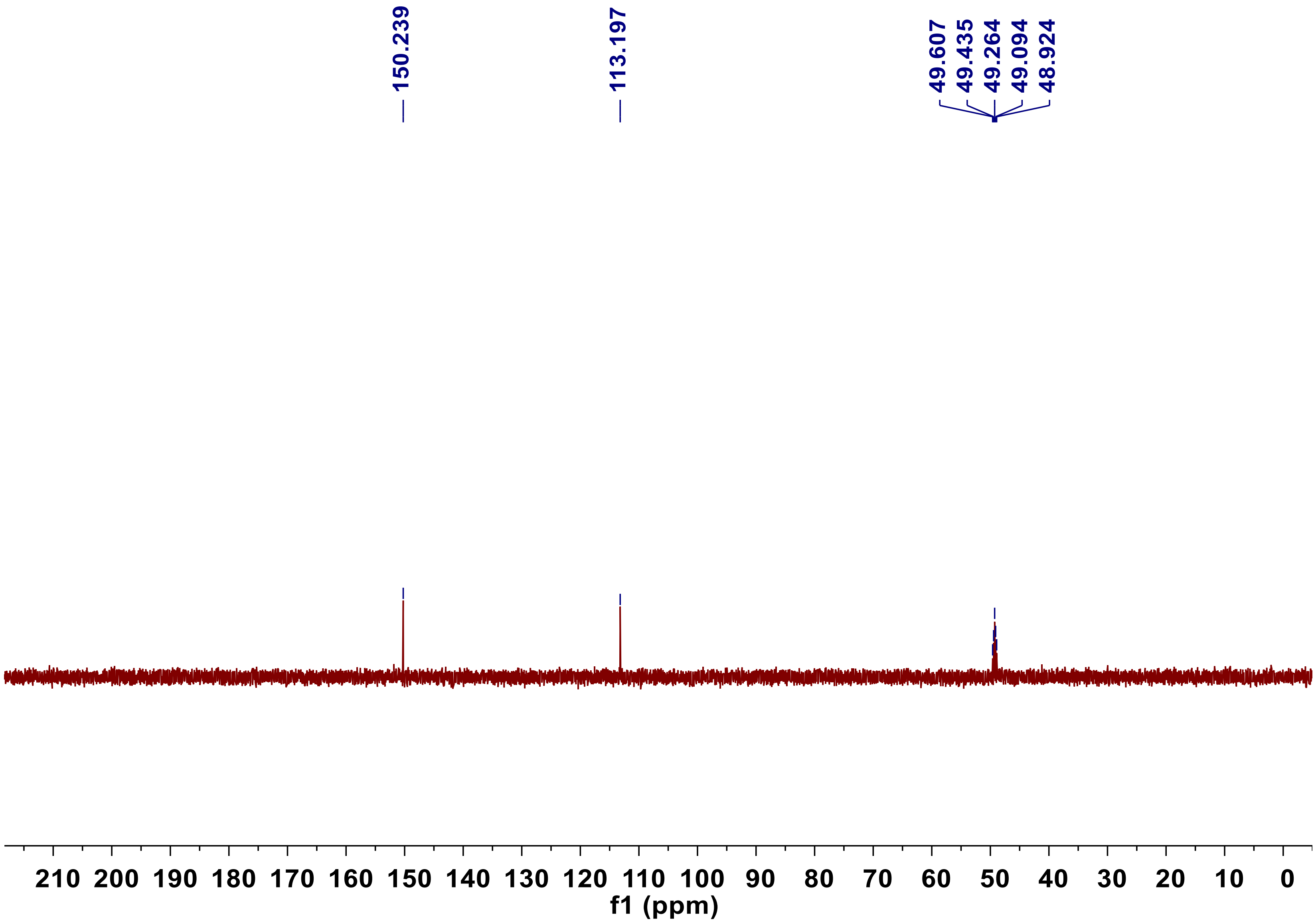

Figure S68 DEPT-135 spectrum of **12**

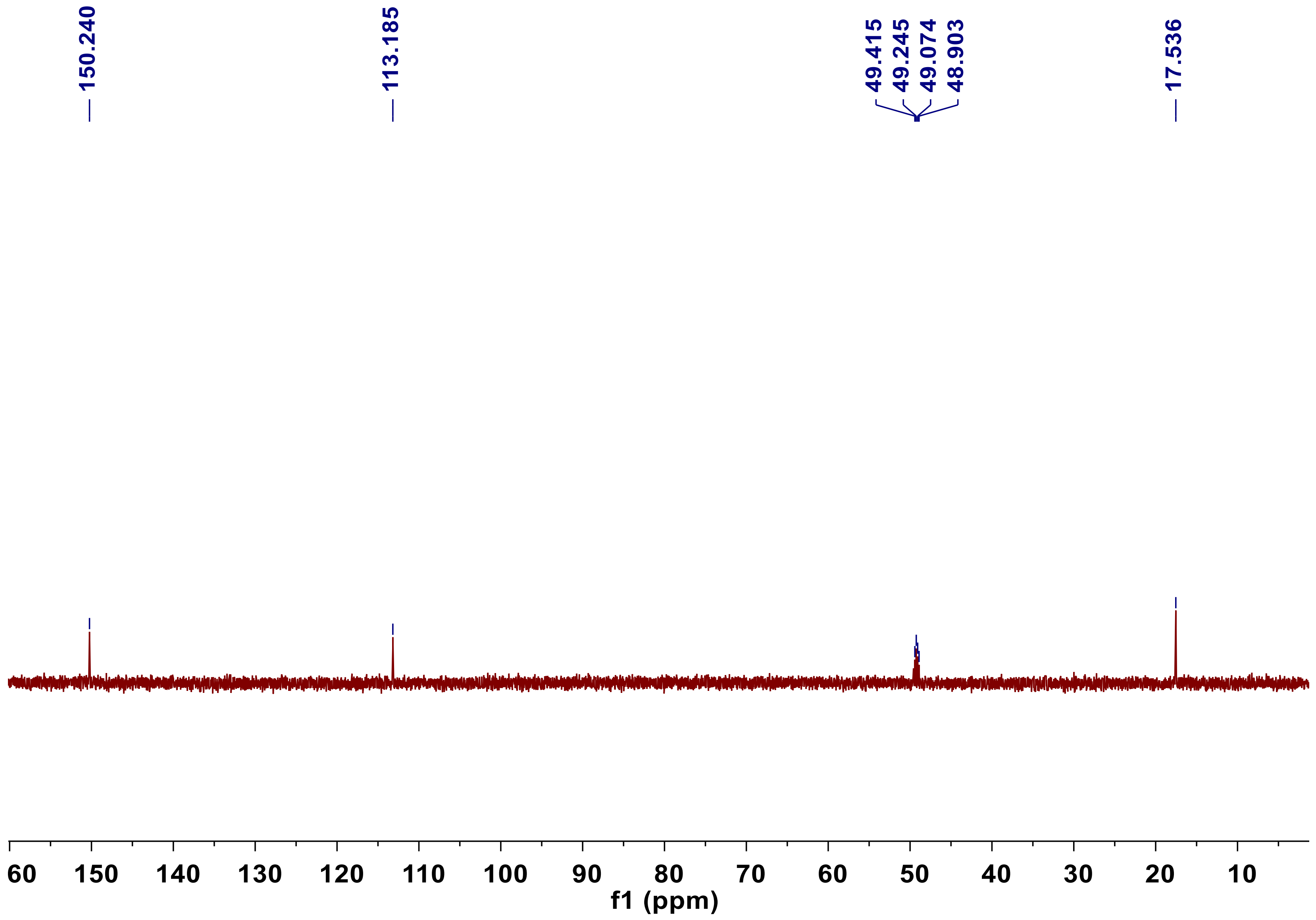

Figure S69 HSQC spectrum of **12**

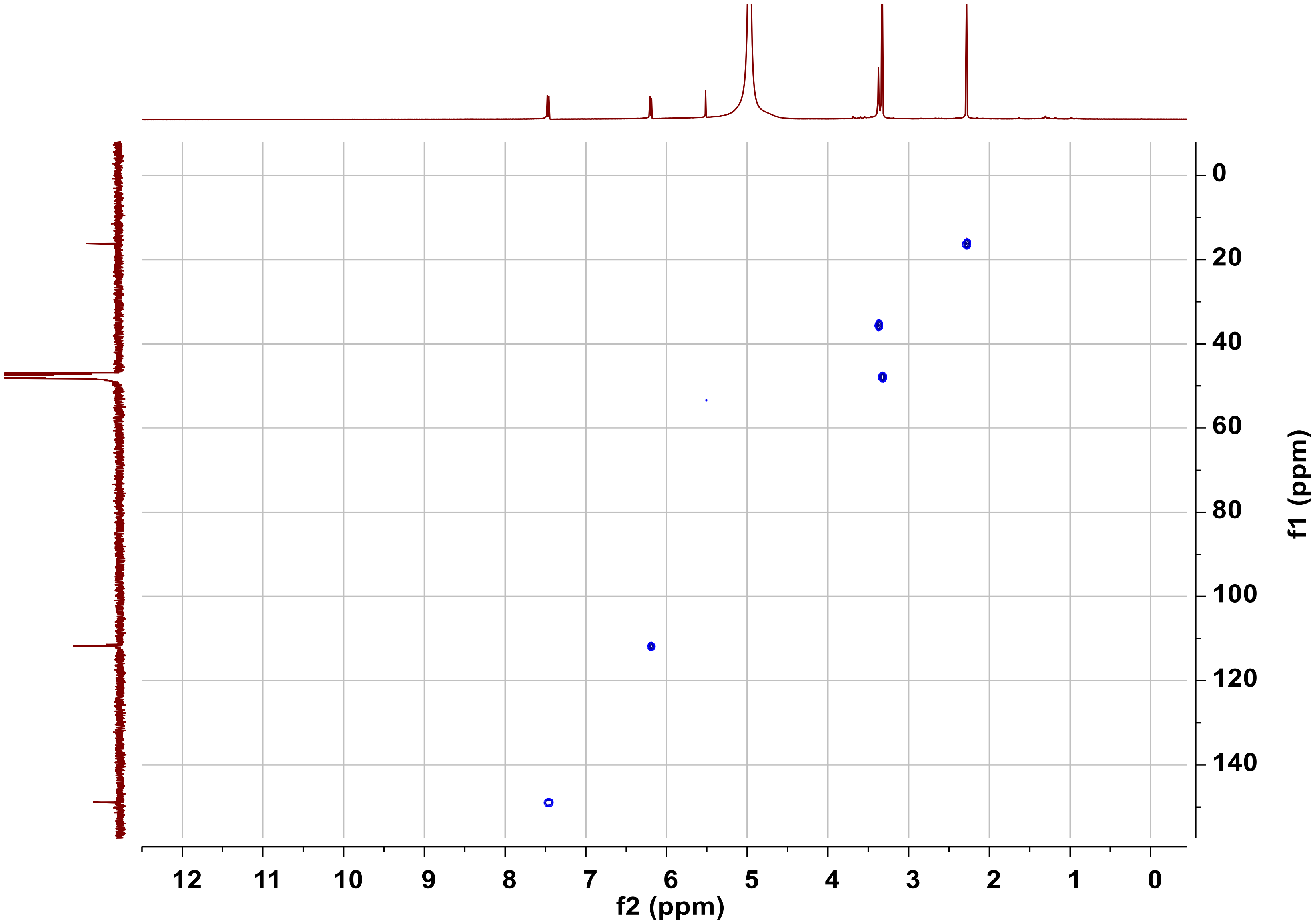

Figure S70 HMBC spectrum of **12**

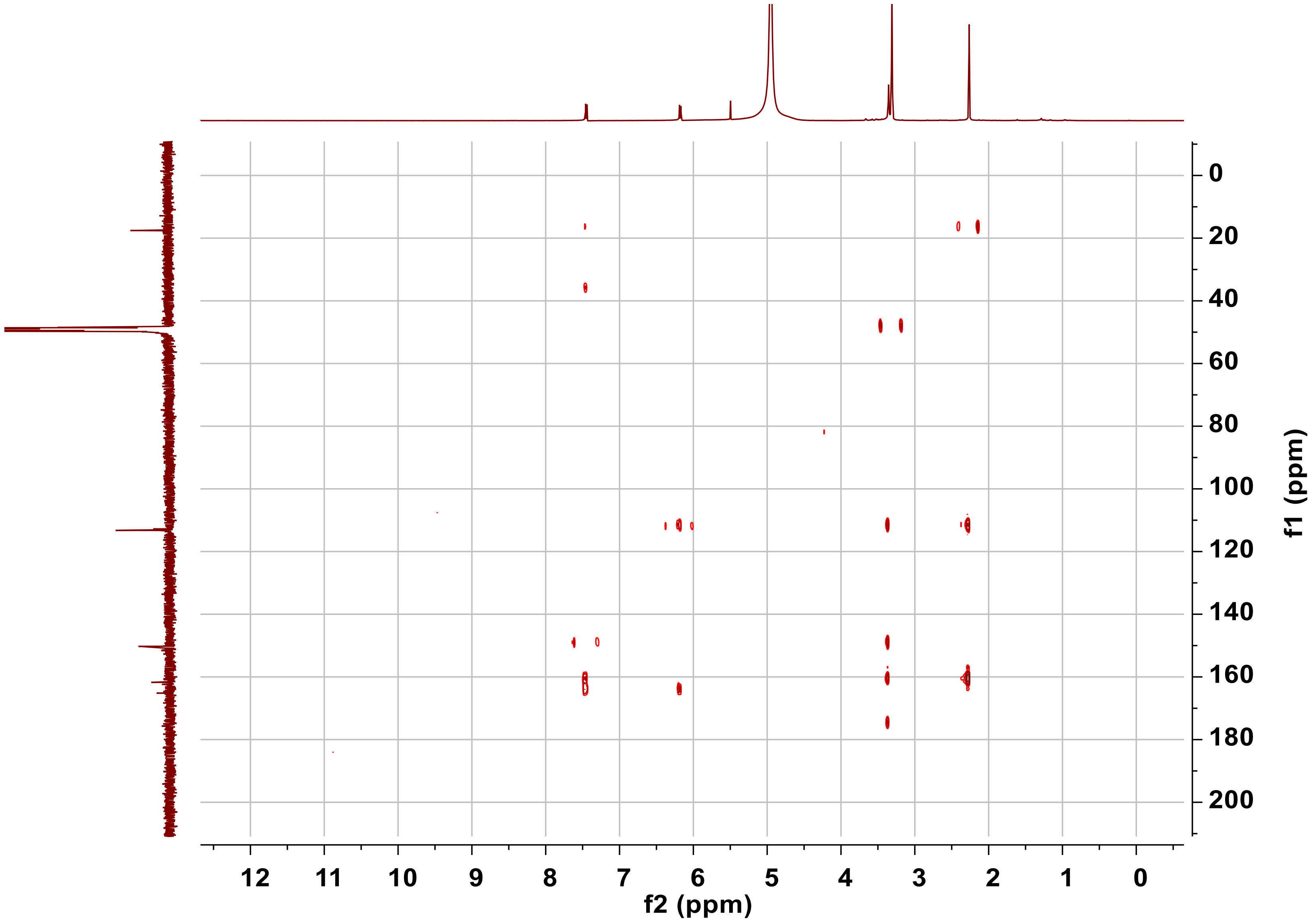

Figure S71 ^{1}H-^{1}H COSY spectrum of **12**

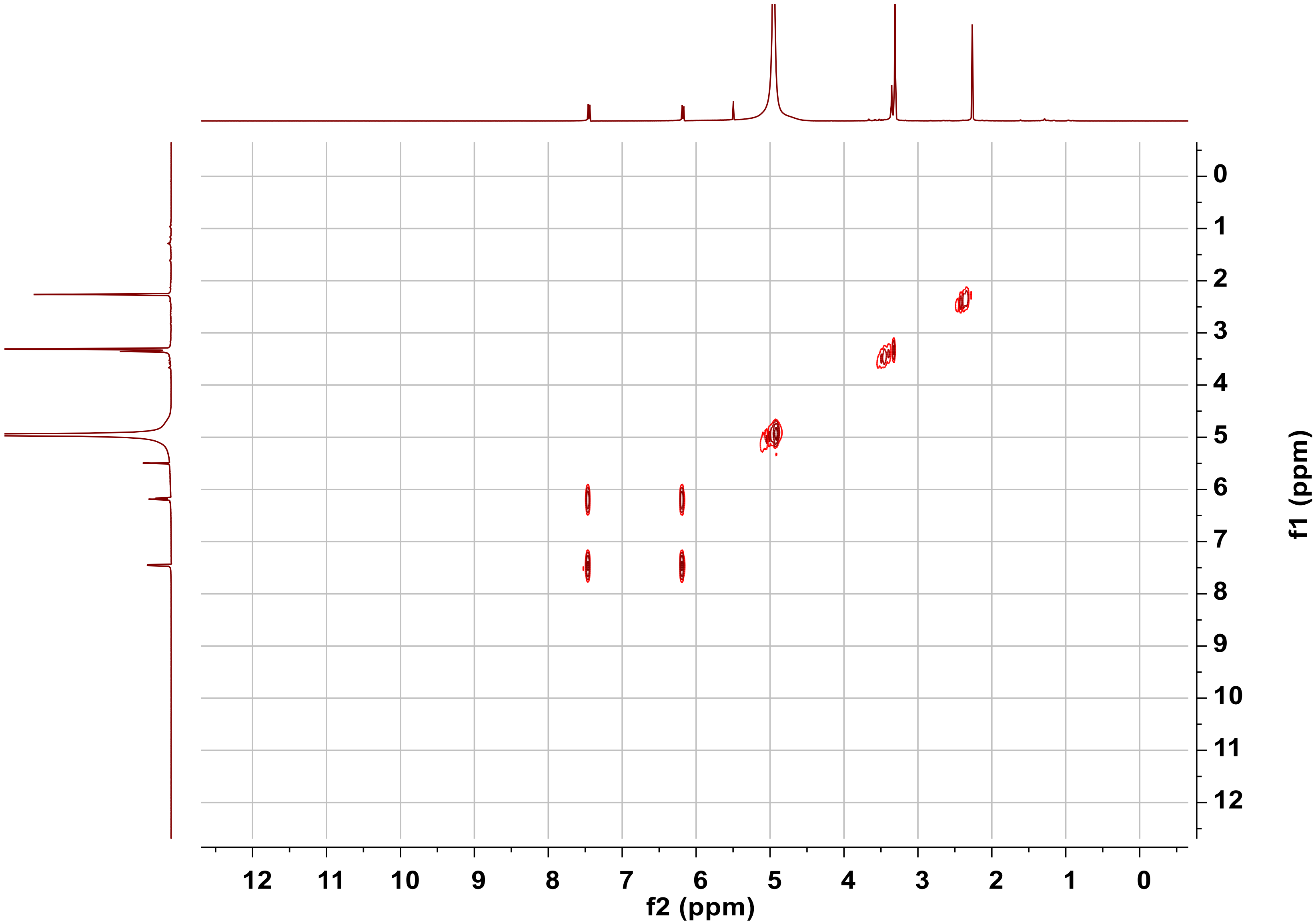

Figure S72 Energy lowest conformers and populations of Diaporpyrone B (**10**) R and optimized coordinates (method: B3LYP/6-31+G(d)).

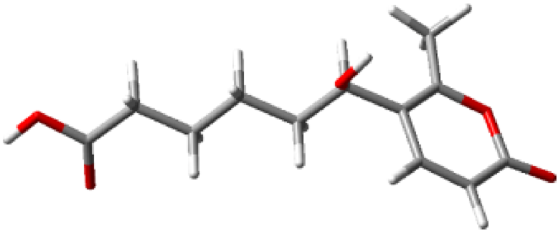

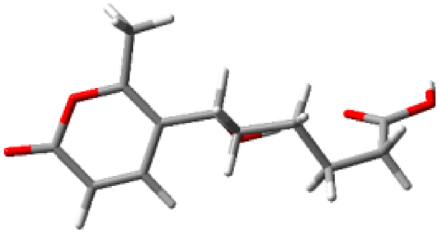

Diaporpyrone B (**10**) R _con1: 26.1%

Diaporpyrone B (**10**) R _con2: 28.06%

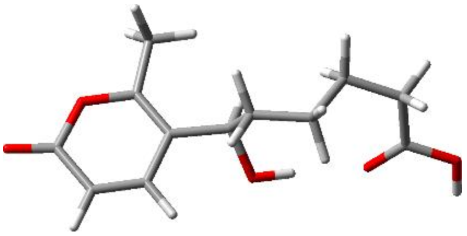

Diaporpyrone B (**10**) R _con3: 45.84

Standard orientation of Diaporpyrone B (**10**) R _con1:

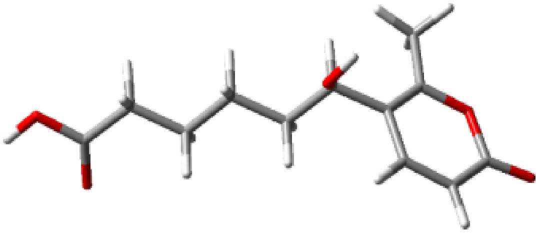

C	3.68605	-1.53535	0.65625
C	4.56128	-0.94507	-0.31719
O	4.09741	0.23965	-0.8871
C	2.90476	0.82989	-0.58459
C	2.06893	0.26945	0.3322
C	2.49999	-0.9463	0.95747
C	0.71522	0.84519	0.71095
C	-0.442	-0.00154	0.17485
O	5.64516	-1.34883	-0.6946
C	2.72111	2.07093	-1.39348
C	-1.82349	0.60735	0.4323
C	-2.95598	-0.25614	-0.13262
O	0.57793	0.90019	2.14206
C	-4.33072	0.36066	0.1183

C	-5.48068	-0.45883	-0.41108
O	-5.40073	-1.51355	-1.00078
O	-6.66992	0.12946	-0.15002
H	4.01508	-2.45216	1.12836
H	1.85495	-1.39627	1.70412
H	0.62023	1.85831	0.3112
H	-0.29073	-0.13295	-0.90212
H	-0.38414	-1.00145	0.62115
H	3.60898	2.70284	-1.31204
H	2.59845	1.81612	-2.45067
H	1.85331	2.64704	-1.08134
H	-1.96716	0.74552	1.50832
H	-1.86667	1.60778	-0.01701
H	-2.81183	-0.39939	-1.20876
H	-2.91772	-1.25395	0.31704
H	1.2315	1.51717	2.49596
H	-4.40475	1.35688	-0.33252
H	-4.50441	0.51518	1.1892
H	-7.38065	-0.42757	-0.50747

Standard orientation of Diaporpyrone B (**10**) R _con2:

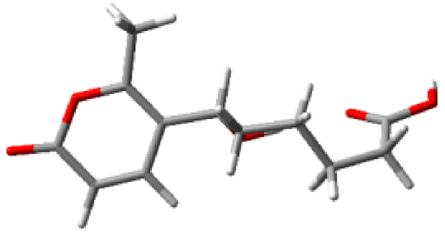

C	-3.34618	-1.56669	-0.43269
C	-4.34625	-0.55196	-0.2588
O	-3.85202	0.72596	-0.00492
C	-2.52666	1.04149	0.08042
C	-1.5759	0.08139	-0.07836
C	-2.02739	-1.25253	-0.34315
C	-0.08721	0.33748	0.03585
C	0.46253	-0.14387	1.39432
O	-5.55648	-0.67403	-0.30754
C	-2.3582	2.49554	0.37276
C	1.97685	0.07117	1.56728
C	2.84475	-1.10006	1.0633
O	0.52683	-0.31479	-1.08468
C	4.2568	-0.68338	0.65358
C	4.29472	0.11533	-0.62606

O	3.3525	0.32413	-1.36727
O	5.52377	0.58404	-0.89727
H	-3.68988	-2.57269	-0.63669
H	-1.28275	-2.02739	-0.48543
H	0.10691	1.41196	-0.03931
H	-0.07843	0.40929	2.16951
H	0.21127	-1.20127	1.53888
H	-2.734	2.72405	1.37484
H	-1.31926	2.81142	0.31943
H	-2.94155	3.0883	-0.33635
H	2.26085	1.00187	1.06176
H	2.19985	0.23934	2.62538
H	2.92056	-1.8558	1.85065
H	2.36444	-1.60031	0.21958
H	1.46771	-0.0714	-1.13345
H	4.89621	-1.55919	0.49518
H	4.75323	-0.09804	1.43469
H	5.50087	1.07398	-1.73602

Standard orientation of Diaporpyrone B (**10**) R _con3:

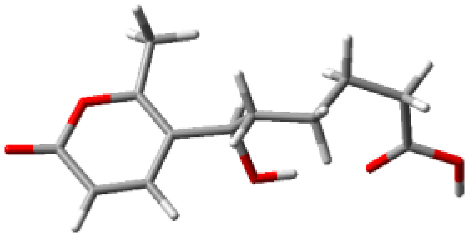

C	-3.50819	-1.49148	0.46129
C	-4.39248	-0.45666	0.00608
O	-3.7624	0.69842	-0.45204
C	-2.41007	0.88017	-0.48917
C	-1.56873	-0.09751	-0.05651
C	-2.16235	-1.30795	0.42863
C	-0.06017	0.0381	-0.03046
C	0.44481	0.37636	1.38902
O	-5.6094	-0.46956	-0.02667
C	-2.08305	2.23499	-1.02347
C	1.96856	0.30066	1.55491
C	2.76749	1.27706	0.67212
O	0.48094	-1.20028	-0.51274
C	4.20122	0.81719	0.40884
C	4.28972	-0.37191	-0.51732
O	3.35646	-0.92936	-1.06333

O	5.55899	-0.77096	-0.70522
H	-3.95945	-2.40671	0.82252
H	-1.50598	-2.10088	0.76841
H	0.23841	0.8345	-0.71581
H	-0.01994	-0.31383	2.10182
H	0.08205	1.3789	1.64604
H	-2.59177	2.39345	-1.97786
H	-1.01502	2.37629	-1.17005
H	-2.43759	3.00664	-0.33336
H	2.28802	-0.72809	1.36323
H	2.21468	0.48689	2.60564
H	2.79751	2.26163	1.14774
H	2.27549	1.4294	-0.2923
H	1.42932	-1.08242	-0.69191
H	4.79703	1.61677	-0.04437
H	4.72151	0.55805	1.33745
H	5.56837	-1.53067	-1.3109

Figure S73 Energy lowest conformers and populations of Diaporpyrone B (**10**) S and optimized coordinates (method: B3LYP/6-31+G(d)).

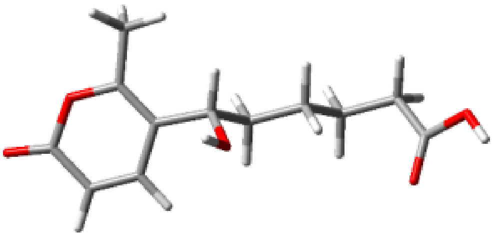

Diaporpyrone B (**10**) S _con1: 12.48%

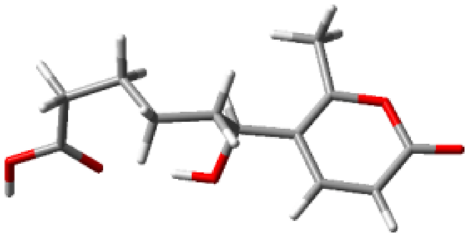

Diaporpyrone B (**10**) S _con2: 71.39%

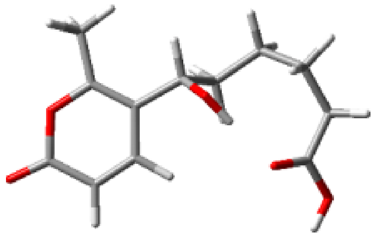

Diaporpyrone B (**10**) S _con3: 16.13%

Standard orientation of Diaporpyrone B (**10**) S _con1:

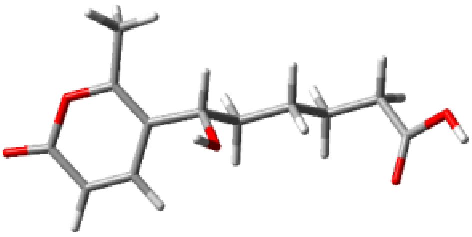

C	-3.56478	-1.65686	-0.24797
C	-4.549	-0.62789	-0.43332
O	-4.10006	0.66946	-0.19043
C	-2.82955	0.99074	0.19001
C	-1.8925	0.01845	0.36297
C	-2.30108	-1.336	0.13264
C	-0.45128	0.28923	0.76028
C	0.51847	0.06895	-0.40356
O	-5.71175	-0.75323	-0.7692
C	-2.69167	2.46942	0.34042
C	1.96798	0.44092	-0.07781
C	2.90165	0.25519	-1.27902
O	-0.05141	-0.59259	1.82396
C	4.35455	0.64055	-0.99132
C	5.08389	-0.31609	-0.07856
O	4.79227	-1.47404	0.12407
O	6.16617	0.26098	0.48937
H	-3.87671	-2.67898	-0.42027
H	-1.57221	-2.12503	0.28207

H	-0.35135	1.32181	1.10557
H	0.16443	0.66563	-1.25149
H	0.46251	-0.9803	-0.71707
H	-2.77015	2.95592	-0.63683
H	-1.74159	2.75524	0.78518
H	-3.50139	2.85746	0.96326
H	2.31751	-0.16377	0.7634
H	2.00727	1.48716	0.25192
H	2.54829	0.87045	-2.11334
H	2.86438	-0.78302	-1.6239
H	-0.58048	-0.39784	2.60831
H	4.93289	0.66464	-1.92328
H	4.42746	1.64831	-0.57336
H	6.6273	-0.40204	1.0291

Standard orientation of Diaporpyrone B (**10**) S _con2:

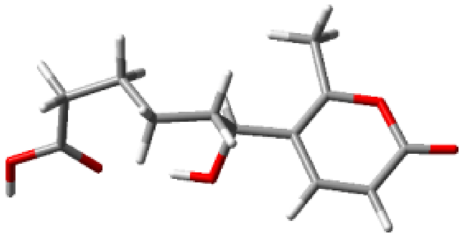

C	3.50812	-1.49149	0.46075
C	4.39259	-0.45665	0.00588
O	3.7625	0.69877	-0.45203
C	2.41015	0.88061	-0.489
C	1.56882	-0.09709	-0.05654
C	2.16235	-1.3078	0.42824
C	0.06021	0.03825	-0.03029
C	-0.44481	0.37448	1.38966
O	5.60942	-0.46957	-0.02694
C	2.08332	2.23566	-1.02288
C	-1.96855	0.2984	1.55541
C	-2.76751	1.27599	0.6739
O	-0.48064	-1.19945	-0.51437
C	-4.20132	0.81653	0.41013
C	-4.28998	-0.37134	-0.51765
O	-3.35658	-0.92915	-1.0631
O	-5.55939	-0.76879	-0.70746
H	3.95948	-2.40683	0.82161
H	1.50584	-2.10071	0.76778
H	-0.23857	0.83563	-0.71442

H	-0.08216	1.37674	1.64796
H	0.01992	-0.31656	2.10163
H	2.43773	3.00705	-0.3324
H	1.01532	2.37708	-1.16963
H	2.59224	2.39447	-1.97709
H	-2.21461	0.4832	2.6064
H	-2.288	-0.73008	1.36232
H	-2.27553	1.42961	-0.29032
H	-2.79765	2.2599	1.15085
H	-1.42932	-1.08195	-0.69206
H	-4.72127	0.55591	1.33852
H	-4.79717	1.61683	-0.04168
H	-5.569	-1.52794	-1.31382

Standard orientation of Diaporpyrone B (**10**) S _con3:

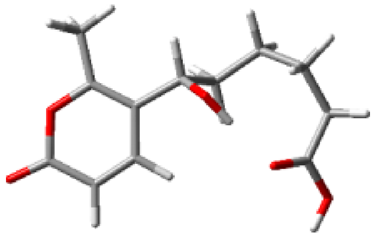

C	-2.46301	-1.83886	-0.02459
C	-3.72787	-1.18743	-0.21704
O	-3.68749	0.20343	-0.17175
C	-2.55422	0.93981	0.03064
C	-1.35235	0.32779	0.21088
C	-1.3386	-1.10483	0.18103
C	-0.04334	1.07449	0.43275
C	0.85344	1.00527	-0.81663
O	-4.81453	-1.70168	-0.40906
C	-2.87534	2.3974	-0.00324
C	2.12617	1.86974	-0.77609
C	3.31629	1.40577	0.08409
O	0.59523	0.59887	1.62432
C	4.02413	0.12929	-0.38976
C	3.41454	-1.18028	0.04046
O	2.50895	-1.33536	0.83924
O	4.01769	-2.22712	-0.54534
H	-2.45305	-2.92102	-0.04714
H	-0.3961	-1.62116	0.33312
H	-0.26028	2.12513	0.63028
H	0.24177	1.35119	-1.6567
H	1.09113	-0.03927	-1.04491

H	-3.70874	2.61135	0.67075
H	-3.18751	2.69074	-1.01028
H	-2.02894	3.01661	0.28294
H	1.84675	2.87885	-0.45077
H	2.48281	1.98151	-1.80688
H	3.02898	1.30649	1.13286
H	4.06131	2.20675	0.05634
H	1.24681	-0.08857	1.40147
H	4.13246	0.10969	-1.47916
H	5.05054	0.10719	-0.00456
H	3.61418	-3.04952	-0.22152
